# A systematic umbrella review of the association of prescription drug insurance and cost-sharing with drug use, health services use, and health

**DOI:** 10.1186/s12913-022-07554-w

**Published:** 2022-03-03

**Authors:** G. Emmanuel Guindon, Tooba Fatima, Sophiya Garasia, Kimia Khoee

**Affiliations:** 1 Centre for Health Economics and Policy Analysis, McMaster University, Room 229, 1280 Main Street West, Hamilton, ON L8S 4K1 Canada; 2 Department of Health Research Methods, Evidence, and Impact, McMaster University, Hamilton, ON Canada; 3 Department of Economics, McMaster University, Hamilton, ON Canada

**Keywords:** Insurance, Cost-sharing, Copayment, Prescription drug, Review

## Abstract

**Background:**

Increasing spending and use of prescription drugs pose an important challenge to governments that seek to expand health insurance coverage to improve population health while controlling public expenditures. Patient cost-sharing such as deductibles and coinsurance is widely used with aim to control healthcare expenditures without adversely affecting health.

**Methods:**

We conducted a systematic umbrella review with a quality assessment of included studies to examine the association of prescription drug insurance and cost-sharing with drug use, health services use, and health. We searched five electronic bibliographic databases, hand-searched eight specialty journals and two working paper repositories, and examined references of relevant reviews. At least two reviewers independently screened the articles, extracted the characteristics, methods, and main results, and assessed the quality of each included study.

**Results:**

We identified 38 reviews. We found consistent evidence that having drug insurance and lower cost-sharing among the insured were associated with increased drug use while the lack or loss of drug insurance and higher drug cost-sharing were associated with decreased drug use. We also found consistent evidence that the poor, the chronically ill, seniors and children were similarly responsive to changes in insurance and cost-sharing. We found that drug insurance and lower drug cost-sharing were associated with lower healthcare services utilization including emergency room visits, hospitalizations, and outpatient visits. We did not find consistent evidence of an association between drug insurance or cost-sharing and health. Lastly, we did not find any evidence that the association between drug insurance or cost-sharing and drug use, health services use or health differed by socioeconomic status, health status, age or sex.

**Conclusions:**

Given that the poor or near-poor often report substantially lower drug insurance coverage, universal pharmacare would likely increase drug use among lower-income populations relative to higher-income populations. On net, it is probable that health services use could decrease with universal pharmacare among those who gain drug insurance. Such cross-price effects of extending drug coverage should be included in costing simulations.

**Supplementary Information:**

The online version contains supplementary material available at 10.1186/s12913-022-07554-w.

## Background

As the US strives to reduce its uninsurance rate, it faces an intensifying challenge of increasing out-of-pocket costs in employer-sponsored health insurance [1, 2]. All the while Canada is debating how best to provide drug insurance to all its residents [3]. Canada is often cited as the only high-income country with universal health insurance coverage lacking universal coverage for prescription drugs [4]. Increasing spending and use of prescription drugs pose an important challenge to governments that seek to expand health insurance coverage to improve population health while controlling public expenditures. Patient cost-sharing such as deductibles and coinsurance is widely used with aim to control healthcare expenditures without adversely affecting health [5].

Since the seminal RAND Health Insurance Experiment [6], numerous studies have examined, at various times and across diverse settings, the impact of health insurance generally, and drug insurance in particular, on utilization and health outcomes. For example, in the US, the introduction of Medicare Part D in 2003 and the Affordable Care Act in 2010 have generated a wealth of new research [7, 8]. Likewise in Canada, the prospect of universal pharmacare and important changes to provincial drug programs such as the 1997 public/private prescription drug program that covered all Québec residents and British Columbia’s adoption of income-based Pharmacare in 2003 in place of an age-based drug benefits program have resulted in an abundance of new analyses [3, 9, 10]. Countless reviews have examined the impact of prescription drug insurance and drug cost-sharing on an array of outcomes such as drug use, health services use, and health, in varied settings and among heterogenous populations. To our knowledge, there has not been an attempt to assess the quality and synthesize evidence from existing reviews. In addition to identifying the strength/credibility of combined associations from reviews to present an objective and comprehensive synthesis of the evidence, such a review of reviews can identify knowledge gaps in the literature, provide useful guidance for future reviews, and have greater implications for policy and practice.

We conducted a systematic umbrella review in order to provide a closer examination of what policy introductions of prescription drug coverage (with and without cost-sharing) would mean for both individuals and governments financing this coverage. We examined reviews that studied the association between having prescription drug coverage (primary and supplementary), as well as varying types and levels of cost-sharing, and:

1. the utilization of prescription drugs (i.e., own-price effects on drug use);
2. the utilization of healthcare services (i.e., cross-price effects on the use of health services such as physician, emergency department, and inpatient services);
3. health outcomes (i.e., own-price effects on health outcomes);

We also examined the degree to which the associations identified in 1–3 above differed across levels of socioeconomic status (SES, e.g., income, education), populations of differing health status such as the chronically ill, age, and sex.

## Methods

A review protocol was prepared in advance and registered with PROSPERO (CRD42017052018). We searched five electronic bibliographic databases: MEDLINE, Embase, Scopus, EconLit, and Health Systems Evidence. Grey literature was searched via the New York Academy of Medicine Grey Literature Report, Open Grey, Google, and Google Scholar. Eight specialty journals (BMC Health Services Research, Health Affairs, Healthcare Policy, Health Economics, Journal of Health Economics, Health Economics, Policy and Law, Health Services Research, and Medical Care Research and Review) and two working paper repositories (RePEc, Research Papers in Economics and the National Bureau of Economic Research working paper series) were ‘hand-searched.’ We examined references of included reviews and of reviews that cited key studies using Web of Science and Google Scholar. The database search was last updated on September 15, 2020. At least two reviewers, using distillerSR, screened titles and abstracts of citations to determine relevance, then full text if relevance was unclear.

### Inclusion and exclusion criteria

Types of studies: all reviews (e.g., narrative, rapid, scoping, systematic, meta-analysis, meta-regression). Types of interventions: (1) insurance: all studies that examined the expansion of prescription drug insurance, irrespective of the insurance provider (e.g., government, employers, professional associations) and studies that examined partial or full-delisting of prescription drugs from insurance coverage; (2) cost-sharing: all studies that examined any form of direct patient payment for prescription drugs including, but not limited to, fixed copayment, coinsurance, ceilings, and caps. Types of outcomes: all reviews that included as an outcome any of drug utilization, health services utilization, or health outcomes. Time period: all reviews published since January 2000. Languages: we included only studies written in English and French. We excluded reviews that focused solely on low- and middle-income countries.

### Quality assessment and data extraction

We used the Assessment of Multiple Systematic Reviews (AMSTAR) measurement tool as a methodological guide [11]. Although AMSTAR’s focus is primarily on the reporting quality of reviews, we paid particular attention to the quality assessment conducted in each review. At least two reviewers independently extracted detailed study characteristics for each included review using a standardized form, including all AMSTAR 2 items (see Additional file 1). The following study characteristics were extracted, where possible: citation, type of review, population investigated, research question, outcomes studied, whether there was an ‘a priori design’ and duplicate study selection and data extraction, the comprehensiveness of the search including if grey literature was searched, year/month of last search, whether the keywords/search strategy were reported, total number of studies included, total number of studies included that focused on drug insurance and/or cost-sharing, whether a list of included and excluded studies were provided, whether the characteristics of the included studies were provided, whether the scientific quality of the included studies was assessed, documented, and used appropriately in formulating conclusions, whether the methods used to combine the findings of studies were appropriate, whether the likelihood of publication bias was assessed, whether funding and competing of interests were clearly reported, key results for each of drug use, healthcare services utilization, and health, and reviews’ conclusion (as stated by the authors). In assessing the quality of the included studies, we paid particular attention to the following components: ‘a priori’ design; duplicate study selection and data extraction; systematic search strategy; presentation of characteristics of included studies and list of excluded studies and reasons for exclusion; quality assessment of included studies; and the generalizability of the findings. We did not compute total scores as empirical evidence does not support their use [12–14]. We created summary tables, organized by outcome and subgroup, using our completed standardized forms. For each study, we highlighted the direction and magnitude of the associations. In our descriptive table, we present the study citations, research question, outcomes studied, study selection and extraction process, quality assessment, and limitations/risk of bias. Lastly, given the current policy debate surrounding universal pharmacare in Canada, we also reported the total number of Canadian studies included that focused on drug insurance and/or cost-sharing [3].

## Results

**Fig. 1:**
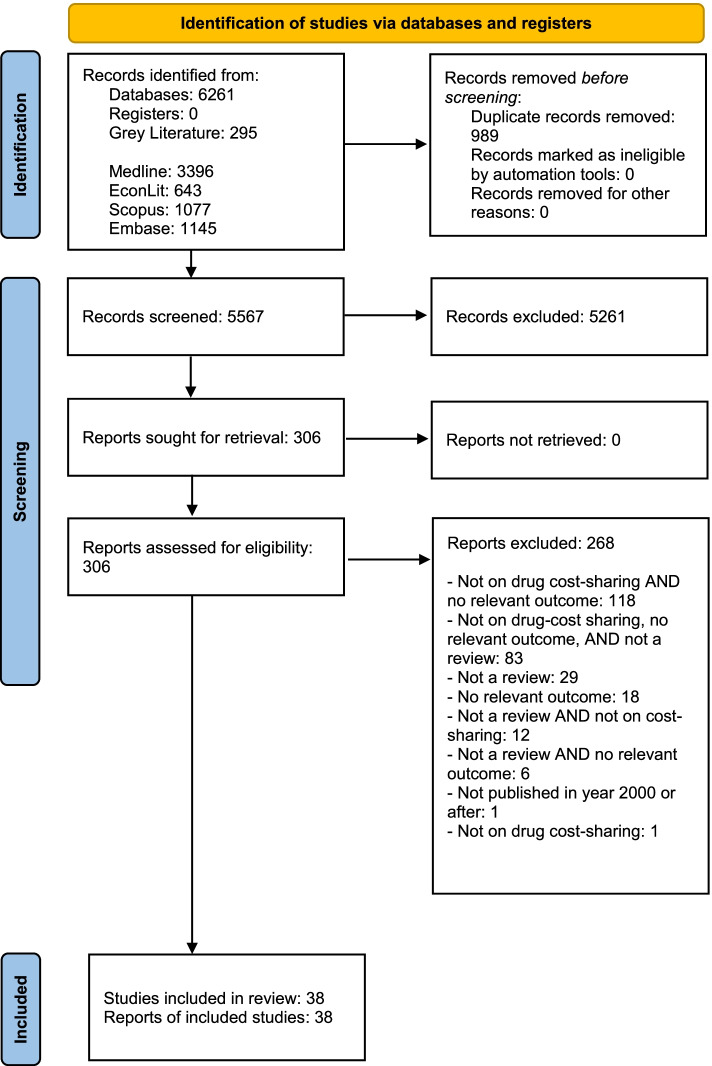
PRISMA 2020 flow diagram for new systematic reviews which included searches of databases and registers only

The database search produced 5567 records after the removal of duplicate citations, from which 5261 were excluded based on the title/abstract screen and 268 were subsequently removed after a full-text screen, yielding 38 reviews that met all inclusion criteria (Fig. 1). Selected study characteristics and our assessment of study’s limitations are presented in Table 1. Detailed characteristics of included studies are presented in the Additional file 1. Of 38 reviews, 16 focused on the general population of which eight also commented on subgroups (e.g., seniors, the poor, and chronically ill), nine focused on seniors (most often on the US Medicare population), and 11 focused on the poor and/or chronically ill. A further two reviews examined drug insurance and cost-sharing among Canadians and one review examined publicly insured populations. Most included reviews were narrative reviews. We included six meta-analyses and one meta-regression. A list of excluded studies and reasons for exclusion is provided in the Additional file 1. We present a synthesis of results in Table 2 and more detailed findings for each reviews in Tables 3, 4, 5, 6, 7, and 8.

**Table 1:** Characteristics of included studies

Authors/year, review type,^**a**^ population, journal	Research question, outcomes	A priori’ design; search; study selection and data extraction	Quality assessment	Limitations / risks of bias
Adams, Soumerai, Ross-Degnan, 2001 [15] - Narrative review (critical review) - US Medicare population (65+ years) - Annual Review of Public Health	The effect of drug coverage on drug utilization, health outcomes, and health care costs in the Medicare population. - drug use: yes - healthcare use: yes - health: yes	- a priori’ design: no - search comprehensive: no - grey literature: yes - year of last search: not reported - # of studies included: total, 37; drugs/cost-sharing/ins, 37; Canada, 0 - duplicate study selection and data extraction: unclear	No formal quality assessment conducted. Criteria were used to assess the validity of findings including study design, appropriateness of study population, data quality and availability, reliability of measures of association and adequacy of statistical analysis. The strengths and limitations of study designs were generally described.	- no ‘a priori’ design; - non-systematic search strategy; - no/unclear duplicate study selection and data extraction; - list of excluded studies not provided; - unclear screening and data extraction process; - study characteristics of studies not provided; - no formal quality assessment of included studies.
Harten, Ballantyne, 2004 [16] - Narrative review (review) - General population (Canadians) - Journal of Pharmaceutical Finance, Economics, and Policy	Canadian evidence of the effects of cost-sharing mechanisms of provincial drug benefit programs on program expenditures, drug utilization and patient health. - drug use: yes - healthcare use: no - health: yes	- a priori’ design: no - search comprehensive: no - grey literature: yes - year of last search: 2002 - # of studies included: total, 7; drugs/cost-sharing/ins, 7; Canada, 7 - duplicate study selection and data extraction: unclear	No formal quality assessment conducted. Limitations of included studies generally discussed.	- no ‘a priori’ design; - unclear duplicate study selection and data extraction; - non-systematic search strategy; - list of excluded studies not provided; - no formal quality assessment of included studies.
Lexchin, Grootendorst, 2004 [17] - Narrative review (systematic review) - The poor and chronically ill - International Journal of Health Services	The effect of drug user fees on drug use and related outcomes in vulnerable populations (the poor and chronically ill). - drug use: yes - healthcare use: yes - health: yes	- a priori’ design: no - search comprehensive: yes - grey literature: yes - year of last search: 2002 - # of studies included: total, 24; drugs/cost-sharing/ins, 24; Canada, 5 - duplicate study selection and data extraction: yes	None	- no ‘a priori’ design; - no quality assessment of included studies.
Rice, Matsuoka, 2004 [18] - Narrative review (review) - Seniors - Medical Care Research & Review	Impact of cost-sharing for medical services and prescription drugs on service use and health status of seniors. - drug use: yes - healthcare use: yes - health: yes	- a priori’ design: no - search comprehensive: no - grey literature: no - year of last search: not reported - # of studies included: total, 22; drugs/cost-sharing/ins, 16; Canada, 4 - duplicate study selection and data extraction: unclear	No formal quality assessment conducted. The limitations of included studies were generally discussed.	- no ‘a priori’ design; - grey literature not searched; - unclear screening and data extraction process (inclusion and exclusion criteria not stated); - list of excluded studies not provided; - no formal quality assessment of included studies; - narrow inclusion criteria (only studies from US and Canada were included).
Gibson, Ozminkowsky, Goetzel, 2005 [19] - Narrative review (review) - General population - American Journal of Managed Care	Do patients respond to increased cost-sharing by substituting less expensive alternatives for medications with higher levels of copayments or coinsurance? - drug use: yes - healthcare use: yes - health: yes	- a priori’ design: no - search comprehensive: no - grey literature: no - year of last search: 2005 - # of studies included: total, 30; drugs/cost-sharing/ins, 30; Canada, 4 - duplicate study selection and data extraction: unclear	None	- no ‘a priori’ design; - narrow inclusion criteria (only studies from US and Canada and that used claims-based data sources were included); - unclear inclusion/exclusion criteria; - list of excluded studies not provided; - study characteristics not clearly presented and/or synthesized; - no formal quality assessment of included studies.
Maio, Pizzi, Roumm, 2005 [20] - Narrative review (review) - Seniors - The Milbank Quarterly	Among seniors, the effects of cost-sharing mechanisms and administrative mechanisms on prescription drug utilization and/or expenditures, other health services, underuse of effective medications, clinical outcomes, adverse events; and on the subject’s behaviour, such as voluntary disenrollment. - drug use: yes - healthcare use: yes - health: yes	- a priori’ design: no - search comprehensive: unclear - grey literature: no - year of last search: 2003 - # of studies included: total, 16; drugs/cost-sharing/ins, 7; Canada, 3 - duplicate study selection and data extraction: unclear	No formal quality assessment. The quality of quasi-experimental studies was generally discussed but not of randomized studies.	- no ‘a priori’ design; - no/unclear duplicate study selection and data extraction; - non-systematic search strategy; - list of excluded studies not provided; - grey literature not searched; - no formal quality assessment of included studies.
Briesacher, Gurwitz, Soumerai, 2007 [21] - Narrative review (review) - General population - J Gen Intern Med	To identify patient-, medication-, and provider-level factors that influence the relationship between medication adherence and medication costs. - drug use: yes - healthcare use: no - health: no	- a priori’ design: no - search comprehensive: yes - grey literature: no - year of last search: 2006 - # of studies included: total, 19; drugs/cost-sharing/ins, 17; Canada, 0 - duplicate study selection and data extraction: no	No formal quality assessment of included studies. Limitations of included studies were generally discussed.	- no ‘a priori’ design; - no/unclear duplicate study selection and data extraction; - grey literature not searched; - study characteristics of included studies not provided; - no formal quality assessment of included studies.
Gemmil, Costa-Font, McGuire, 2007 [22] - Meta-regression (meta-regression) - General population - Health Economics	To determine an estimate for drug-price elasticity using meta-regression analysis. - drug use: yes - healthcare use: no - health: no	- a priori’ design: no - search comprehensive: no - grey literature: no - year of last search: not reported - # of studies included: total, 31; drugs/cost-sharing/ins, 31; Canada, 6 - duplicate study selection and data extraction: unclear	No formal quality assessment of included studies. Limitations of included studies were generally discussed.	- no ‘a priori’ design; - no/unclear duplicate study selection and data extraction; - search strategy poorly described; - grey literature not searched; - study characteristics of included studies not provided; - poorly justified or unclear exclusion criteria; - no formal quality assessment of included studies.
Goldman, Joyce, Zheng, 2007 [23] - Narrative review (review) - General population - JAMA	Associations among cost-sharing features of prescription drug benefits and use of prescription drugs, use of non-pharmaceutical services, and health outcomes. - drug use: yes - healthcare use: yes - health: yes	- a priori’ design: no - search comprehensive: no - grey literature: no - year of last search: not reported - # of studies included: total, 132; drugs/cost-sharing/ins, 132; Canada, 26 - duplicate study selection and data extraction: unclear	No formal quality assessment of included studies. Limitations of included studies were generally discussed.	- no ‘a priori’ design; - non-systematic search strategy; - grey literature not searched; - no formal quality assessment of included studies. - no/unclear duplicate study selection and data extraction; - list of excluded studies not provided.
Gemmil, Thomson, Mossialos, 2008 [24] - Narrative review (review) - General population - International Journal for Equity in Health	The impact of prescription drug charges on efficiency and equity. - drug use: yes - healthcare use: yes - health: yes	- a priori’ design: no - search comprehensive: yes - grey literature: yes - year of last search: 2006 - # of studies included: total, 173; drugs/cost-sharing/ins, 173; Canada, 28 - duplicate study selection and data extraction: unclear	None; ‘quality’ was assessed by looking at study design, type of data analyzed, and techniques used for analysis but quality was not assessed beyond that.	- no ‘a priori’ design; - no/unclear duplicate study selection and data extraction; - study characteristics of included studies not provided. - list of excluded studies not provided; - no formal quality assessment of included studies.
Remler, Greene, 2009 [25] - Narrative review (review) - General population - Annual Review of Public Health	To determine the effects of cap and co-payment policies on rational use of medicines, healthcare utilization, health outcomes, and costs. - drug use: yes - healthcare use: yes - health: yes	- a priori’ design: no - search comprehensive: unclear - grey literature: yes - year of last search: not reported - # of studies included: unclear - duplicate study selection and data extraction: unclear	None	- no ‘a priori’ design; - no/unclear duplicate study selection and data extraction; - non-systematic search strategy; search strategy poorly described; - unclear screening and data extraction process; - list of included and excluded studies not provided; - study characteristics of included studies not provided; - no formal quality assessment of included studies.
Green, Maclure, et al., 2010 [26] - Narrative review (systematic) - General population - Report: The Cochrane Library	The effects of a pharmaceutical policy restricting the reimbursement of selected medications on drug use, health care utilization, health outcomes, and costs. - drug use: yes - healthcare use: yes - health: yes	- a priori’ design: yes - search comprehensive: yes - grey literature: yes - year of last search: 2009 - # of studies included: total, 29; drugs/cost-sharing/ins, 29; Canada, 11 - duplicate study selection and data extraction: yes	Included studies were appraised using Cochrane EPOC criteria for interrupted time series.(1)	- restrictive inclusion criteria limits the usefulness of the review.
Holst, 2010 [27] - Narrative review (systematic in-depth review) - General population - Working paper: Wissenschaftszentrum Berlin für Sozialforschung (WZB)	Does direct patient cost-sharing improve the efficiency of use of resources in health care? What effects does it have on social inequality of health opportunities in the population, and the political goal of reducing this? - drug use: yes - healthcare use: yes - health: no	- a priori’ design: no - search comprehensive: unclear - grey literature: yes - year of last search: not reported - # of studies included: not clearly reported - duplicate study selection and data extraction: unclear	None	- no ‘a priori’ design; - search strategy poorly described; - unclear screening and data extraction process (inclusion and exclusion criteria not stated); - list of included and excluded studies not provided; - study characteristics of included studies not provided; - no formal quality assessment of included studies.
Polinski, Kilabuk, et al., 2010 [28] - Narrative review (systematic) - US Medicare population (65+ years) - Journal of the American Geriatrics Society	To assess the extent to which Medicare Part D’s cost-sharing provisions and drug coverage rules affected the under- and overuse of specific drugs and classes. - drug use: yes - healthcare use: no - health: no	- a priori’ design: no - search comprehensive: no - grey literature: no - year of last search: 2009 - # of studies included: total, 26; drugs/cost-sharing/ins, 26; Canada, 0 - duplicate study selection and data extraction: yes	The Newcastle-Ottawa Scale for cohort studies was used.(2) Unclear how domains were operationalized and assessed.	- no ‘a priori’ design; - non-systematic search strategy; - grey literature not searched; - unclear exclusion criteria; - list of excluded studies not provided; - formal quality assessment of included studies poorly described and discussed; only global ratings provided; unclear how any of the domains were operationalized and assessed.
Swartz, 2010 [29] - Narrative review (synthesis) - General population - The Synthesis Project (The Robert Wood Johnson Foundation)	What is known and unknown about the effects of consumer cost sharing? - drug use: yes - healthcare use: yes - health: yes	- a priori’ design: no - search comprehensive: no - grey literature: unclear - year of last search: unclear - # of studies included: total, unclear; drugs/cost-sharing/ins, unclear; Canada, unclear - duplicate study selection and data extraction: no	No formal quality assessment conducted. However, in general, greater weight was given to studies that used data from natural experiments with credible comparison groups, as well as studies that relied on data from larger numbers of people and from people who were representative of subgroups of people.	- no ‘a priori’ design; - list of excluded studies not provided; - no/unclear duplicate study selection and data extraction; - search strategy poorly described; - poorly justified or unclear exclusion criteria; - study characteristics of included studies not provided; - no formal quality assessment of included studies;
Baicker, Goldman, 2011 [30] - Narrative review (review) - General population - Journal of Economic Perspectives	To determine the relationship between patient cost-sharing and healthcare spending growth. - drug use: yes - healthcare use: yes - health: yes	- a priori’ design: no - search comprehensive: unclear - grey literature: not reported - year of last search: not reported - # of studies included: total, not reported; drugs/cost-sharing/ins, not reported; Canada, 1 - duplicate study selection and data extraction: unclear	None	- no ‘a priori’ design; - no/unclear duplicate study selection and data extraction; - search strategy not described; - list of included and excluded studies not provided; - no quality assessment of included studies; - study characteristics of included studies not provided; - focus on US studies limits the usefulness of the review.
Polinski, Donohue, et al., 2011 [31] - Narrative review (systematic) - US Medicare population (65+ years) - Journal of the American Geriatrics Society	The extent to which Medicare Part D’s cost-sharing provisions and drug coverage rules affected the under- and overuse of specific drugs and classes. - drug use: yes - healthcare use: no - health: no	- a priori’ design: no - search comprehensive: no - grey literature: no - year of last search: 2010 - # of studies included: total, 19; drugs/cost-sharing/ins, 19; Canada, 0 - duplicate study selection and data extraction: yes	The Newcastle-Ottawa Scale for cohort studies was used.(2) Unclear how domains were operationalized and assessed.	- no ‘a priori’ design; - unclear inclusion/exclusion criteria; - non-systematic search strategy; - search strategy poorly described; - grey literature not searched; - formal quality assessment of included studies poorly described and discussed; only global ratings provided; unclear how any of the domains were operationalized and assessed.
Eaddy, Cook, et al., 2012 [32] - Narrative review (review) - General population - Pharmacy and Therapeutics	To assess the relationship between patient cost-sharing, medication adherence, clinical, utilization, and economic outcomes. - drug use: yes - healthcare use: yes - health: yes	- a priori’ design: no - search comprehensive: yes - grey literature: no - year of last search: 2008 - # of studies included: total, 160; drugs/cost-sharing/ins, 160; Canada, 17 - duplicate study selection and data extraction: unclear	No formal quality assessment of included studies. Limitations of included studies were generally discussed.	- no ‘a priori’ design; - unclear duplicate study selection and data extraction; - search strategy poorly described; - grey literature not searched; - unclear screening and data extraction process (inclusion and exclusion criteria not clearly stated); - study characteristics of included studies not provided; - list of excluded studies not provided; - no formal quality assessment of included studies.
Lemstra, Blackburn et al., 2012 [33] - Meta-analysis (meta-analysis) - Statin users - Canadian Journal of Cardiology	To provide estimates of risk indicators associated with nonadherence to statin medications. - drug use: yes - healthcare use: no - health: no	- a priori’ design: no - search comprehensive: yes - grey literature: no - year of last search: 2011 - # of studies included: total, 67; drugs/cost-sharing/ins, 6; Canada, 1 - duplicate study selection and data extraction: unclear	RCTs: Used the Delphi list to evaluate quality; Observational studies: assessed patient selection process, criteria for inclusion and exclusion, patient identification process, comparative information for the patients who were not enrolled in the study, attrition, lost-to follow-up, statistical analysis, and controlling for confounding variables. A score of 5/9 and 5/8 was required for an RCT and an observational study, respectively, to be included. Unclear how domains were operationalized and assessed.	- no ‘a priori’ design; - no/unclear duplicate study selection and data extraction; - grey literature not searched; - list of excluded studies not provided; - arbitrary threshold used to categorize the quality of included studies.
Maimaris, Paty, et al., 2013 [34] - Narrative review (systematic) - Individuals with hypertension - PLOS One	The influence of national or regional health systems on hypertension awareness, treatment, and control? - drug use: yes - healthcare use: no - health: yes	- a priori’ design: yes - search comprehensive: yes - grey literature: no - year of last search: 2013 - # of studies included: total, 53; drugs/cost-sharing/ins, 35; Canada, 0 - duplicate study selection and data extraction: yes	Risk of bias for observational study designs were assessed using three domains: selection bias, information bias, and confounding. For RCTs, the Cochrane risk of bias tool was used;(5) Cochrane tool was not described or discussed. Only global ratings provided for both tools. Unclear how domains were operationalized and assessed.	- grey literature not searched; - list of excluded studies not provided; - preponderance of US studies limits the generalizability of the findings. - formal quality assessment of included studies poorly described and discussed; only global ratings provided; unclear how any of the domains were operationalized and assessed.
Pimentel, Lapane, Briesacher, 2013 [35] - Narrative review (systematic) - US Medicare population (65+ years) in long-term care - Drugs Aging	The impact of US Medicare Part D on the long-term care context, specifically costs to long-term care residents, providers and payers; prescription drug coverage and utilization; and clinical and administrative outcomes. - drug use: yes - healthcare use: no - health: yes	- a priori’ design: no - search comprehensive: yes - grey literature: yes - year of last search: 2013 - # of studies included: total, 19; drugs/cost-sharing/ins, 11; Canada, 0 - duplicate study selection and data extraction: no	Quality rating scale developed by Downs and Black (1998) to assess study quality.(6) Unclear which items were removed and which were kept. Some global ratings reported. Full assessment not provided. Unclear how any of the domains were operationalized and assessed.	- no ‘a priori’ design; - list of excluded studies not provided; - no duplicate study selection and data extraction; - formal quality assessment of included studies poorly described and discussed; only global ratings provided; unclear how any of the domains were operationalized and assessed.
Sinnott, Buckley, et al., 2013 [36] - Meta-analysis (meta-analysis) - Publicly insured populations - PLOS One	The effect of copayments for prescriptions on adherence to prescription medicines in publicly insured populations. - drug use: yes - healthcare use: no - health: no	- a priori’ design: no - search comprehensive: yes - grey literature: yes - year of last search: 2012 - # of studies included: total, 7; drugs/cost-sharing/ins, 7; Canada, 0 - duplicate study selection and data extraction: yes	Controlled before-and-after studies and interrupted time series designs were assessed using a modified version of the Cochrane EPOC criteria;(1) Cohort studies were assessed using the Effective Public Health Practice Project component rating scale.(4) Only global ratings provided. Unclear how domains were operationalized and assessed; 6/7 studies rated as weak, 1 study rated as weak/moderate.	- no ‘a priori’ design; - quality assessment: only global ratings provided; unclear how any of the domains were operationalized and assessed; - arbitrary threshold used to categorize the quality of included studies; - small number of included studies limits the generalizability of the findings.
Kiil, Houlberg, 2014 [37] - Narrative review (systematic) - General population - European Journal of Health Economics	What is the extent to which copayment reduces individual demand for services on which it is imposed, has adverse health effects, and give rise to distributional consequences. - drug use: yes - healthcare use: yes - health: yes	- a priori’ design: no - search comprehensive: no - grey literature: yes - year of last search: 2011 - # of studies included: total, 47; drugs/cost-sharing/ins, 18; Canada, 9 - duplicate study selection and data extraction: unclear	None	- no ‘a priori’ design; - list of excluded studies not provided; - unclear duplicate study selection and data extraction; - non-systematic search strategy; - no formal quality assessment of included studies.
Mann, Barnieh, et al., 2014 [38] - Narrative review (systematic) - Individuals with cardiovascular-related chronic disease - PLOS One	The impact of drug insurance and varying levels of patient cost-sharing on medication adherence, clinical and economic outcomes in patients with cardiovascular-related chronic disease. - drug use: yes - healthcare use: no - health: yes	- a priori’ design: no - search comprehensive: yes - grey literature: no - year of last search: 2013 - # of studies included: total, 11; drugs/cost-sharing/ins, 11; Canada, 3 - duplicate study selection and data extraction: yes	Cochrane risk of bias tool for RCT (5), and Cochrane EPOC (1) taxonomy for controlled before-after studies and interrupted time series designs were used to assess the quality of the studies. Seven components were rated as low, mid, high risk. Unclear how domains were operationalized and assessed.	- no ‘a priori’ design; - list of excluded studies not provided; - grey literature not searched; - unclear how any of the quality criteria were operationalized and assessed; - quality assessment not explicitly taken into account.
Kesselheim, Huybrechts et al., 2015 [39] - Narrative review (systematic) - General population - American Journal of Public Health	To determine how expansions or restrictions in prescription drug insurance have affected patients’ health outcomes or their use of health care services. - drug use: yes - healthcare use: yes - health: yes	- a priori’ design: no - search comprehensive: yes - grey literature: no - year of last search: 2014 - # of studies included: total, 23; drugs/cost-sharing/ins, 23; Canada, 0 - duplicate study selection and data extraction: yes	Used guidelines outlined in Cochrane Handbook for Systematic Reviews of Interventions (Cochrane risk of bias tool). Summary scores presented for each component and overall (low, unclear, high), 22/23 scored low/unclear. Unclear how domains were operationalized and assessed.	- no ‘a priori’ design; - grey literature not searched; - surprisingly low number of studies identified; - list of excluded studies not provided; - only summary scores presented; unclear what led to low quality scores; - 22 of 23 included studied were conducted in the United States which limits the generalizability of the findings.
Luiza, Chavez et al., 2015 [40] - Narrative review (systematic) - General population - Report: The Cochrane Library	The effects of cap and co-payment policies on rational use of medicines, healthcare utilization, health outcomes and costs. - drug use: yes - healthcare use: yes - health: yes	- a priori’ design: yes - search comprehensive: yes - grey literature: yes - year of last search: 2013 - # of studies included: total, 32; drugs/cost-sharing/ins, 32; Canada, 9 - duplicate study selection and data extraction: yes	For RCTs, and Interrupted time series (ITS)/Repeated Measures (RM), assessed risk of bias using the Cochrane EPOC tool criteria that provides nine standard domains for RCTs and seven domains for ITS/RM. Summary ratings and justifications were provided for each study.(1) Confidence in overall estimates graded using GRADE.	- restrictive inclusion criteria limits the usefulness of the review.
Aziz, Hatah, et al., 2016 [41] - Narrative review (systematic) - General population - Patient Prefer Adherence	How do payment scheme affect patients’ adherence to medications. - drug use: yes - healthcare use: no - health: no	- a priori’ design: no - search comprehensive: yes - grey literature: no - year of last search: 2015 - # of studies included: total, 21; drugs/cost-sharing/ins, 21; Canada, 2 - duplicate study selection and data extraction: no	27 items from Downs and Black’s checklist for measuring study quality was used.(6) Only global ratings provided. Yes/No provided for all items, without any supporting information. Quality assessment not otherwise used.	- no ‘a priori’ design; - no duplicate study selection; - grey literature not searched; - concerning low number of studies included; - quality of the included studies not used in interpreting findings or formulating conclusions.
Banerjee, Khandelwal, et al., 2016 [42] - Narrative review (systematic) - Individuals with cardiovascular diseases - Open Heart	What are the barriers and facilitators to adherence to secondary cardiovascular disease prevention medications at health system level. - drug use: yes - healthcare use: no - health: no	- a priori’ design: yes - search comprehensive: yes - grey literature: yes - year of last search: 2015 - # of studies included: total, 25; drugs/cost-sharing/ins, 4; Canada, 0 - duplicate study selection and data extraction: yes	Observational studies: three domains were assessed: selection bias, information bias and confounding. RCTs: the Cochrane risk of bias tool was used. Assessment not provided (only presence or absence of bias for each component provided along with generic total assessment [low, unclear, high]). 3/4 relevant studies assessed as low risk of bias but high risk of confounding and 1 study assessed as unclear risk of bias. Unclear how domains were operationalized and assessed.	- quality assessment: only summary scores presented; unclear what led to low quality scores; - small number of relevant included studies limits the usefulness and generalizability of the findings. - list of excluded studies was not provided.
Doshi, Li et al., 2016 [43] - Narrative review (systematic) - Individuals using specialty drugs - Am J Manag Care	What is the impact of cost-sharing on utilization of speciality drugs indicated for rheumatoid arthritis, multiple sclerosis, and cancer, and on use of nondrug medical services, health outcomes and spending? - drug use: yes - healthcare use: yes - health: yes	- a priori’ design: no - search comprehensive: unclear - grey literature: no - year of last search: 2014 - # of studies included: total, 19; drugs/cost-sharing/ins, 19; Canada, 0 - duplicate study selection and data extraction: yes	No formal quality assessment conducted. Limitations of included studies generally discussed.	- no ‘a priori’ design; - non-systematic search strategy; - grey literature not searched; - list of excluded studies not provided; - no formal quality assessment of included studies.
Powell, Saloner, Sabik, 2016 [44] - Narrative review (systematic) - Medicaid beneficiaries - Medical Care Research and Review	What are the effects of cost-sharing, focusing on low-income populations in US, on health care utilization, and spending - drug use: yes - healthcare use: yes - health: no	- a priori’ design: no - search comprehensive: yes - grey literature: yes - year of last search: 2014 - # of studies included: total, unclear; drugs/cost-sharing/ins, unclear; Canada, 0 - duplicate study selection and data extraction: unclear	No formal quality assessment conducted. Natural experiments, studies with larger sample sizes (*n* > 1000), and studies that used representative samples from multiple states, considered of higher quality.	- no ‘a priori’ design; - no/unclear duplicate study selection and data extraction; - list of included and excluded studies not provided; - results not clearly synthesized - US focus limits the generalizability of the findings.
Gourzoulidis, Kourlaba, et al., 2017 [45] - Narrative review (systematic) - Individuals with heart failure or diabetes mellitus - Health Policy	To determine the association between copayment, medication adherence and outcomes in patients with heart failure and diabetes mellitus. - drug use: yes - healthcare use: yes - health: yes	- a priori’ design: no - search comprehensive: unclear - grey literature: no - year of last search: not reported - # of studies included: total, 38; drugs/cost-sharing/ins, 11; Canada, 0 - duplicate study selection and data extraction: yes	A modified version of the EPHPP tool was used where the selection bias domain was replaced with allocation bias.(4) Only global ratings provided. Unclear how any of these criteria were operationalized and assessed.	- no ‘a priori’ design; - no duplicate study selection and data extraction; - grey literature not searched; - list of excluded studies not provided; - unclear how any of the quality criteria were operationalized and assessed; - arbitrary threshold used to categorize the quality of included studies; - limited generalizability of the findings (10/11 relevant studies used US data).
Park, Martin, 2017 - Narrative review (systematic) [7] - Medicare Part D enrolees - Health Services Research	To update a a review on whether Medicare Part D changed drug utilization and out-of-pocket (OOP) costs overall and within subpopulations, and to identify evidence gaps. - drug use: yes - healthcare use: no - health: no	- a priori’ design: no - search comprehensive: yes - grey literature: yes - year of last search: 2015 - # of studies included: total, 65; drugs/cost-sharing/ins, 62; Canada, 0 - duplicate study selection and data extraction: no	Yes; the authors used a predefined criteria and a fixed coding guide to systematically assess the risk of bias in each study. Rubric included bias common to observational studies: comparability between intervention and control groups, attrition, data collection and quality, measurement error, missing data, and reliability and validity of the outcome measures.	- no ‘a priori’ design; - list of excluded studies not provided; - no duplicate study selection and data extraction; - US focus limits the generalizability of the findings.
Gupta, McColl et al., 2018 [46] - Narrative review (scoping) - General population (Canadians) - Patient Prefer Adherence	The extent, determinants, and consequences of cost-related nonadherence to prescription medications in Canada. - drug use: yes - healthcare use: yes - health: yes	- a priori’ design: no - search comprehensive: yes - grey literature: no - year of last search: 2018 - # of studies included: total, 37; drugs/cost-sharing/ins, 20; Canada, 20 - duplicate study selection and data extraction: unclear	No formal quality assessment conducted. Limitations of included studies generally discussed.	- no ‘a priori’ design; - no/unclear duplicate study selection and data extraction; - grey literature not searched; - list of excluded studies not provided; - no formal quality assessment of included studies.
Ofori-Asenso, Jakhu et al., 2018 [47] - Meta-analysis (meta-analysis) - 65+ years statins users - Journals of Gerontology: Medical Sciences	What factors are associated with non-adherence and/or discontinuation of statins among older persons (65+)? - drug use: yes - healthcare use: no - health: no	- a priori’ design: yes - search comprehensive: yes - grey literature: yes - year of last search: 2016 - # of studies included: total, 45; drugs/cost-sharing/ins, 6; Canada, 0 - duplicate study selection and data extraction: yes	Observational studies were assessed using a set of questions from the the National Institute of Health (NIH) Quality Assessment Tool for Observational Cohort and Cross-Sectional Studies (unclear which questions were used).(7) RCTs were assessed using the Joanna Briggs Institute’s critical appraisal checklist for RCTs.(8) Tools not described or discussed. Only global ratings provided. Unclear how any of the domains were operationalized and assessed.	- grey literature not searched; - list of excluded studies not provided; - quality assessment: only global ratings provided; unclear how any of the domains were operationalized and assessed; - arbitrary threshold used to categorize the quality of included studies.
Schneider, Gaedke et al., 2018 [48] - Meta-analysis (meta-analysis) - Individuals with chronic cardiovascular disease - Int J Clin Pract	Effect of characteristics of pharmacotherapy on non-adherence in chronic cardiovascular disease. - drug use: yes - healthcare use: no - health: no	- a priori’ design: no - search comprehensive: yes - grey literature: unclear - year of last search: 2016 - # of studies included: total, 31; drugs/cost-sharing/ins, 17; Canada, 1 - duplicate study selection and data extraction: no	A reporting checklist, Strengthening the Reporting of Observational Studies in Epidemiology (STROBE) was used.(9) Each of the 22 criteria received scores of 0 to 1, total scores then transformed into percentage. Full assessment not provided. Only total scores provided. Unclear how any of the criteria were operationalized and assessed.	- no ‘a priori’ design; - list of excluded studies not provided; - no duplicate study extraction; - unclear if grey literature was searched; - formal quality assessment of included studies conducted using a reporting checklist, poorly described and discussed; only global scored provided; unclear how any of the domains were operationalized and assessed.
Cheen, Tan et al., 2019 [49] - Meta-analysis (meta-analysis) - Individuals with any of 6 common chronic diseases - Int J Clin Pract	To assess prevalence of primary medication non-adherence in six common chronic diseases (asthma, chronic obstructive pulmonary disease, depression, diabetes mellitus, hyperlipidaemia, hypertension and osteoporosis), to identify and categorize factors associated with primary medication nonadherence; and to explore characteristics that contributed to heterogeneity between studies. - drug use: yes - healthcare use: no - health: no	- a priori’ design: no - search comprehensive: yes - grey literature: no - year of last search: 2018 - # of studies included: total, 33; drugs/cost-sharing/ins, 8; Canada, 0 - duplicate study selection and data extraction: yes	The Cochrane risk of bias tool was used to assess clinical trials,(5) the Newcastle-Ottawa Scale for assessing cohort studies,(2) and the National Heart, Lung and Blood Institute Quality Assessment Tool for assessing cross-sectional studies (NIH).(7) Unclear how domains were operationalized and assessed for Newcastle-Ottawa scale and the National Heart, Lung and Blood Institute Quality Assessment Tool.	- no ‘a priori’ design; - grey literature not searched; - list of excluded studies not provided; - formal quality assessment of included studies poorly described and discussed; - results may not be generalizable to other chronic conditions not included.
Kolasa, Kowalcyzk, 2019 [50] - Narrative review (systematic) - General population - Health Economics, Policy and Law	Association between prescription drug cost-sharing and health care consumption and health outcomes. - drug use: no - healthcare use: yes - health: yes	- a priori’ design: yes - search comprehensive: yes - grey literature: no - year of last search: 2016 - # of studies included: total, 18; drugs/cost-sharing/ins, 18; Canada, 2 - duplicate study selection and data extraction: yes	Used a checklist, adapted from Gardner, Machin, Campbell (1986), for the assessment of the statistical content of medical studies. Tool not adequately described. Assessment not provided (only presence or absence of each component provided). Unclear how any of the domains were operationalized and assessed.(10)	- grey literature not searched; - list of excluded studies not provided; - arbitrary threshold used to categorize the quality of included studies; - unclear how any of the quality criteria were operationalized and assessed; - arbitrary threshold used to categorize the quality of included studies.
Mishuk, Fasina, Qian, 2019 [51] - Narrative review (systematic) - Individuals affected by US federal and state generic drug policies - Research in Social and Administrative Pharmacy	To evaluate the impact of US federal and state generic drug policies on drug use, spending, and patient outcomes. - drug use: yes - healthcare use: yes - health: no	- a priori’ design: no - search comprehensive: yes - grey literature: no - year of last search: 2017 - # of studies included: total, 34; drugs/cost-sharing/ins, 24; Canada, 0 - duplicate study selection and data extraction: yes	The EPHPP tool was used to assess all included quantitative studies, however it is unclear if all eight domains were assessed or if a modified version of the tool was used. Only global ratings provided. Unclear how any of the domains were operationalized and assessed.(4)	- no ‘a priori’ design; - grey literature not searched; - list of excluded studies not provided; - results not clearly synthesized; - quality assessment: only global ratings provided; unclear how any of the domains were operationalized and assessed; - US focus limits the generalizability of the findings.

^a^We categorized reviews using three types: 1) narrative review, 2) meta-analysis, and 3) meta-regression; in brackets, we indicated the terminology used by authors. Notes (1) to (9), See Additional file 1: Appendix B which provides a short description of the quality assessment and/or risk of bias tools utilized

**Table 2:** Summary of results: association between prescription drug insurance/cost-sharing and drug use, health services use, and health

**Drug use: general population**	
Lack of insurance and higher cost-sharing were associated with lower drug use [19, 22–24, 27, 29, 30, 37, 39, 40, 51];	
– Own-price η ≈ − 0.1 to − 0.6 [29];	
– Own-price η ≈ − 0.6 to − 0.8; based on aggregate data [24];	
– Own-price η ≈ − 0.2 to − 0.6; based on individual/household data [24];	
– Own-price η ≈ − 0.2 to − 0.6 [23, 30];	
– Own-price η ≈ − 0.2 [22];	
– Own-price η ≈ − 0.1 to − 0.4 [19];	
Restriction to reimbursement was associated with decreased drug use, either immediately after policy change or long-term [26];	
Among Canadians, the introduction of or increases in drug cost-sharing was associated with either no change or lower use (essential and non-essential) [16];	
The magnitude of association between cost-sharing and drug use depended on drug class, [23, 27, 30, 51] condition of patients, [23] and patient population [37];	
Lack of drug insurance and higher cost-sharing were associated with lower medication adherence and a higher risk of cost-related nonadherence [19, 21, 32, 36, 41, 46, 51];	
– Overall, a $10 increase was associated with a 3.8% decrease in adherence [32];	
– Publicly insured patients with copayments had higher odds of reporting nonadherence relative to those without copayments [36];	
Duration of coverage and type of coverage modified the magnitude of the association between cost-sharing and adherence [21];	
Drug insurance restrictions were associated with lower drug use and adherence [39];	
*Essential drugs:*	
– With increased cost-sharing, both essential and non-essential drug use was decreased but the decrease was larger for nonessential drugs [29];	
– Mixed evidence that higher cost-sharing was associated with lower use of essential drugs, relative to nonessential; reductions in the use of non-essential drugs were usually slightly larger [23, 25];	
*Drug type: generic drugs, preferred brand-name drugs, over-the-counter drugs:*	
– Limited evidence that increased generic-brand cost-sharing differential was associated with changes in patterns of drug use [19];	
– Limited evidence that increased cost-sharing for prescription drugs was associated with higher use of over-the-counter drugs [19];	
– Increases in drug cost-sharing for non-preferred brand-name drugs was associated with lower use of non-preferred brand-name drugs and higher use of preferred brand-name drugs [19];	
– Statin users required to make a copayment were more likely than others to be nonadherent [17];	
– Among statin users ≥65 years, higher copayment/cost (not necessarily solely drug cost-sharing) increased the likelihood of nonadherence and discontinuation [47].	
**Drug use: older adults, seniors**	
Among older adults, lack of insurance and higher cost-sharing were associated with lower drug use, increased nonadherence and discontinuation [24, 27, 29, 47];	
Older people were not found to be more sensitive to price than the general population [24];	
– Own-price η, older adults ≈ − 0.1 to − 0.6 [24];	
In the US Medicare population, drug insurance was associated with higher drug use and decreased a risk of cost-related nonadherence [15, 21];	
– The inception of Medicare Part D was associated with an increase in drug use (6 to 13%) [23, 31];	
– Entry into Medicare Part D coverage gaps was associated with lower drug use (9 to 16%) [31];	
– Among US Medicare population in long-term care, drug insurance was associated with lower use of drugs that carry safety concerns, but overall drug utilization may have been unaffected [35];	
Among seniors, there was mixed evidence that higher cost-sharing was associated with lower drug use [20];	
The magnitude of association between cost-sharing and drug use depended on drug class, [15, 28] condition of patients, [15] and, patient population [31];	
*Essential drugs:* Among seniors, higher cost-sharing was associated with lower use of essential drugs [15, 18, 28];	
*Generic drugs:* Entry into Medicare Part D coverage gaps was associated with increased use of generic drugs (20%) [7, 28, 51].	
**Drug use: socioeconomic status, chronically ill**	
Among the poor and chronically ill, higher cost-sharing was associated with lower drug use [17, 23, 27];	
– Own-price η, poor ≈ − 0.05 to − 0.4; based on aggregate data [24];	
– Own-price η, poor ≈ − 0.03 to − 0.2; based on individual/household data [24];	
– Own-price η, poor/chronically ill ≈ − 0.3 to − 0.5 [17];	
Vulnerable populations were more responsive to cost-sharing than non-vulnerable population [37];	
Among individuals with cardiovascular-related chronic disease, drug insurance was associated with increased adherence and persistence to medications [38, 42];	
Among individual with hypertension lower drug cost-sharing was associated with hypertension treatment [34];	
Statin users required to make a copayment were more likely than others to be nonadherent [33];	
Higher cost-sharing was associated with lower use of specialty drugs indicated for rheumatoid arthritis (RA), multiple sclerosis (MS), and cancer [43].	
**Health services use: general population**	
Limiting (expanding) drug insurance was associated with an increase (decrease) in the use of health services (emergency department visits, emergency mental health service, hospitalizations, psychiatric hospitalizations, nursing home admissions [39];	
Higher levels of prescription drug cost-sharing were associated with lower use of health services:	
– Outpatient visits [24, 32, 37, 50];	
– Preventative services [32];	
– Emergency department visits [24, 25, 32, 37, 50];	
– Emergency mental health services [24];	
– Hospitalizations [24, 25, 50];	
– Nursing home admissions [24, 37];	
Higher levels of prescription drug cost-sharing were not associated (or the association was unclear) with lower use of health services:	
– Outpatient visits [19, 23, 25, 26];	
– Home health visits [19];	
– Emergency department visits [19, 23, 26, 40];	
– Hospitalizations [19, 23];	
Among Canadians, it was unclear if cost-related nonadherence was associated with lower health services use (hospitalizations, emergency department visits) [46].	
**Health services use: socioeconomic status, chronically ill, children**	
Higher levels of prescription drug cost-sharing were associated with lower use of health services:	
– Outpatient visits [27];	
– Emergency department visits [17, 23, 25];	
– Hospitalizations [17, 23, 25, 27];	
– Nursing home admissions [17];	
Among individuals with heart failure or diabetes mellitus, higher levels of prescription drug cost-sharing were generally not associated with lower use of health services (outpatient visits, emergency department visits, hospitalizations, or laboratory/diagnostic tests) [45];	
Among individuals affected by US federal and state generic drug policies, government insurance plans with high-cost sharing on generic drugs were associated with less use of health services among children [51].	
**Health: general population**	
Evidence on the association between prescription drug cost-sharing and health suggested that higher drug cost-sharing generally lowered health status [24, 32, 50];	
Evidence on the association between prescription drug cost-sharing and health was limited and/or unclear [16, 19, 23, 25, 26, 29, 30, 46];	
Evidence on the association between prescription drug insurance and health was limited, but generally indicated a positive association [39].	
**Health: older adults, seniors**	
Among seniors, evidence on the association between prescription drug cost-sharing and health was limited and/or unclear [20, 35].	
**Health: socioeconomic status, chronically ill**	
Some evidence that higher cost-sharing was associated with poorer health among the poor and chronically ill [17, 23, 29, 30].

**Table 3:** Results — association between prescription drug insurance/cost-sharing and drug use, general population

Authors, year	Results — drug use, general population
Harten, Ballantyne, 2004 [16]; – Canadians	Found either no change in utilization or a decrease in essential and nonessential medications following introduction of or increases in drug cost-sharing. *Magnitude***:** unclear.
Gibson, Ozminkowsky, Goetzel, 2005 [19];	– Demand for prescription drugs Higher levels of drug cost-sharing resulted in reductions in prescription drug use. *Magnitude:* most estimates of own-price elasticity suggested that a 10% increase in price decreased use by 1 to 4%. – Medication adherence: Patients facing cost-sharing were less likely to adhere to prescribed medications. *Magnitude:* unclear. – Non-preferred vs. preferred brand-name drugs: All studies reviewed showed that increasing drug cost-sharing for non-preferred brand-name drugs decreased use of non-preferred brand-name drugs and increased use of preferred brand-name drugs. *Magnitude:* unclear. – Generic substitution: Little evidence of generic substitution in plans introducing or increasing a generic vs brand cost-sharing differential. *Magnitude:* unclear. – Substitution of over-the-counter drugs for prescription drugs: Limited and inconclusive findings. *Magnitude:* unclear. – Essential medications: Higher levels of prescription drug cost-sharing were associated with a reduction in the consumption of essential medications. *Magnitude:* unclear.
Briesacher, Gurwitz, Soumerai, 2007 [21];	Not having prescription drug coverage was a significant and robust risk factor for cost-related nonadherence in all reviewed studies. *Magnitude***:** unclear; duration of coverage and type of coverage affected the magnitude of associations.
Gemmil, Costa-Font, McGuire, 2007 [23];	Higher cost-sharing was negatively associated with the demand for prescription drugs. *Magnitude*: the demand for prescription drugs was relatively inelastic. The estimated corrected own-price elasticity was − 0.21 (mean standard error 0.026); a 10% increase in cost-sharing was associated with a 2% decrease in pharmaceutical spending.
Goldman, Joyce, Zheng, 2007 [23];	– Demand for prescription drugs Higher cost-sharing was negatively associated with the demand for prescription drugs. *Magnitude*: the demand for prescription drugs was relatively inelastic. Own-price elasticities ranged from − 0.2 to − 0.6; cost-sharing increases of 10% (through either higher copayments or coinsurance) were associated with a 2 to 6% decline in prescription drug use. The magnitude of association depended on class of drug and condition of patients. – Essential and nonessential drug use Mixed effects of the impact of copayments on essential drug use. *Magnitude***:** unclear.
Gemmil, Thomson, Mossialos, 2008 [24];	– Demand for prescription drugs Individuals who faced prescription drug charges were less likely to use prescription drugs while those with insurance coverage were more likely to use them. *Magnitude*: overall, the demand for prescription drugs was almost always inelastic. Studies that used aggregate data generally found that a 10% increase in price resulted in a 0.6 to 8% decrease in use while studies that used individual- or household-level data generally found that a 10% increase in price resulted in a 0.2 to 6% decrease in use. – Volume of drug use: Most studies included found a negative relationship between prescription cost-sharing and levels of prescription drug use while insurance coverage had a positive effect on the volume of drug used. *Magnitude*: unclear. – Brand-name vs generic drugs: The demand for brand-name drugs was more price-elastic than that of generic drugs. *Magnitude*: The demand for brand-name and the demand for generic drugs were both relatively inelastic. – Essential and nonessential drug use: Most studies found that prescription drug charges lowered the use of essential and nonessential drugs, although reductions in the use of nonessential drugs were usually slightly larger. *Magnitude*: unclear.
Remler, Greene, 2009 [25];	There was an inverse association between pharmaceutical cost-sharing and pharmaceutical spending/use. There was mixed evidence that pharmaceutical cost-sharing affected essential drugs differently. *Magnitude*: on average, a 10% increase in pharmaceutical cost-sharing (measured as equivalent coinsurance) resulted in decreases of 2 to 6% in pharmaceutical spending/use.
Green, Maclure, et al., 2010 [26];	Restriction to reimbursement decreased drug use, either immediately after policy implemented or long-term. Impact varied by drug class and whether restrictions were implemented or relaxed. *Magnitude*: unclear.
Holst, 2010 [27]	Consistent findings that increasing prescription cost-sharing reduced drug use and patient compliance to drug therapies. Effect varied depending on class of substance. *Magnitude*: unclear.
Swartz, 2010 [29];	Increased cost-sharing for prescription drugs was associated with declines in use and spending on drugs. The evidence was unclear whether people responded to increased cost-sharing by switching to less expensive, close drug substitutes. With increased cost-sharing, both essential and nonessential drug use was decreased but the decrease was larger for nonessential drugs. M*agnitude*: increased cost-sharing of about 10% was associated with a decline of between 1 and 6% in spending on prescription drugs.
Baicker, Goldman, 2011 [30];	Higher cost-sharing was negatively associated with the demand for prescription drugs. *Magnitude*: The evidence suggested a price elasticity for drug expenditures of − 0.2 to − 0.6. The range reflected differences in responsiveness by drug class and its importance.
Eaddy, Cook, et al., 2012 [32];	Most studies found a statistically significant relationship between increased patient drug cost-sharing and decreased medication adherence. The effect depended on the population and intervention. *Magnitude*: overall, a $10 increase was associated with a 3.8% decrease in adherence.
Sinnott, Buckley, et al., 2013 [36]; – Publicly insured populations	There was a positive association between copayments and nonadherence. *Magnitude*: summary odds ratio for nonadherence was 1.11 (95%CI 1.09, 1.14); publicly insured patients who were required to pay copays for their prescription medicines had 11% higher odds of reporting nonadherence relative to those who faced no copayments.
Kiil, Houlberg, 2014 [37];	Overall, pharmaceutical copayments had negative effects on the use of prescription medicine. The extent to which copayment affected the use of prescription medicine depended on the type of medicine as well as the patient population. *Magnitude*: unclear.
Kesselheim, Huybrechts et al., 2015 [39];	– Prescription drug insurance coverage Three studies examined the impact of drug insurance on patients’ use of drugs and adherence by comparing cohorts of patients with and without coverage. Two of three studies found that those with insurance used more drugs. *Magnitude*: unclear. – Extending drug insurance *Magnitude:* one study examined the effects of extending drug coverage to patients on their drug use and found that the number of prescription fills increased non-significantly by 2 per patient-year. – Drug insurance restriction *S*ix studies evaluated the effects of drug insurance restrictions on drug utilization and adherence. All studies found that drug insurance restrictions led to lower drug utilization and/or adherence. *Magnitude*: unclear.
Luiza, Chavez et al., 2015 [40];	Raising direct patient payments for medicines was found to reduce the use of both important and unimportant drugs. The impact was sometimes uncertain and varied from small to moderate relative reductions. *Magnitude*: unclear.
Aziz, Hatah, et al., 2016 [41];	Lower cost-sharing, higher prescription caps, subsidies, and insurance were associated with higher medication adherence. *Magnitude*: unclear.
Gupta, McColl et al., 2018 [46]; – Canadians	Having prescription drug insurance was significantly associated with having access to prescription medication without financial barriers. High drug costs (> 5% of annual household income or > $20 a month out-of-pocket) was a major determinant of cost-related nonadherence. *Magnitude*: unclear.
Mishuk, Fasina, Qian, 2019 [51]; – Individuals affected by US federal and state generic drug policies	Seven studies found that policies lowering prescription cost-sharing were associated with increased patient’s medication use and adherence, but the impact varied by therapeutic classes. *Magnitude*: unclear

**Table 4:** Results — association between prescription drug insurance and cost-sharing and drug use, for/between specific populations

Authors, year / population^**a**^	Results — drug use, for/between specific populations
***Older adults, seniors***	
Adams, Soumerai, Ross-Degnan, 2001 [15]; – US Medicare population (65+ years)	In the US Medicare population, drug coverage was associated with greater use of all drugs and clinically essential medications. *Magnitude, seniors*: reductions in drug use ranged between 21 and 46% depending on the drug class and condition of patients. *Magnitude, seniors* vs. *non-seniors*: unclear.
Rice, Matsuoka, 2004 [18]; – Seniors	Among seniors, cost-sharing (not necessarily for drugs) was found to reduce the appropriate use of prescription drugs (medications that were thought to improve health status). *Magnitude, seniors*: unclear. *Magnitude, seniors* vs. *non-seniors*: unclear.
Maio, Pizzi, Roumm, 2005 [20]; – Seniors	There was mixed evidence that prescription cost-sharing mechanisms (copayment, coinsurance, and deductible) reduced seniors’ drug use. There was some evidence that for low-income populations, even small copayments, may have led them to reduce their use of effective medications. *Magnitude, seniors*: unclear. *Magnitude, seniors* vs. *non-seniors*: unclear.
Briesacher, Gurwitz, Soumerai, 2007 [21]; – General population	There was strong evidence that among medicare beneficiaries, drug coverage decreased the risk of cost-related medication nonadherence; strong evidence that among medicare beneficiaries and adults 50+, higher cost-sharing increased the risk of cost-related medication nonadherence. *Magnitude, older adults, seniors*: unclear. *Magnitude, older adults, seniors*: vs. *non-older adults, non-seniors*: unclear.
Gemmil, Thomson, Mossialos, 2008 [24] – General population	Older people were not found to be more sensitive to price than the general population. *Magnitude, older adults*: a 10% increase in price led to changes in use for older people ranging from a 5.6% reduction to a 0.9% increase based on non-aggregate data, and one study using aggregate data found a reduction of 5.1%. *Magnitude, older adults* vs. *non-older adults*: among the general population, price elasticity estimates suggested that a 10% increase in price led to a 0.2 to 4.6% decrease in use based on non-aggregate data and a 0.9 to 8.0% decrease in use based on aggregate data.
Holst, 2010 [27]; – General population	Older people responded especially sensitively to cost-sharing. *Magnitude, older adults*: unclear. *Magnitude, older adults* vs. *non-older adults*: unclear.
Polinski, Kilabuk, et al., 2010 [28]; – US Medicare population (65+ years)	The inception of Medicare Part D was associated with a consistent overall increase in drug use. There was little variation in effect estimates between studies evaluating the effect of Part D implementation. Across all studies, entry of Part D beneficiaries into the coverage gap was associated with reduced drug use. *Magnitude, seniors*: the inception of Part D was associated with a 6 to 13% increase in drug use. Changes in use varied according to drug, disease, and population studied. There was little indication that Part D selectively led to greater use of essential, underused drugs than of overused medications. Across all studies, entry of Part D beneficiaries into the coverage gap was associated with 9 to 16% less drug use. Patients who entered the coverage gap were 5 to 11% more likely to report discontinuing, switching, or failing to initiate a medication than were patients who did not enter the coverage gap. Use of generic drugs increased 20% during the coverage gap. *Magnitude, seniors* vs. *non-seniors*: unclear.
Swartz, 2010 [29]; – General population	Cost-sharing reduced use of essential drugs in people with chronic conditions and the elderly. Studies that looked at the Medicare doughnut hole found that elderly reduced drug use when they had to pay full price. M*agnitude, elderly:* one study in the elderly found cost-sharing reduced essential drugs by 9% for essential drugs and 15% for nonessential drugs. M*agnitude, elderly* vs *non-elderly:* unclear.
Baicker, Goldman, 2011 [30]; – General population	One study that examined Medicare Part D found that providing insurance to the elderly led to increased prescription drug use. *Magnitude, seniors*: providing insurance to the elderly led to a 13% increase in prescription drug use. Further interpretation not provided *Magnitude, seniors* vs. *non-seniors*: unclear.
Polinski, Donohue, et al., 2011 [31]; – US Medicare population (65+ years)	In the period after Medicare Part D implementation there was an increase in the use of essential medicines especially in beneficiaries who had been previously uninsured, and of nonessential medicines. During the transition period, dually eligible beneficiaries’ drug use remained largely unchanged. In the coverage gap, when cost-sharing increased, the use of essential and overused medications declined. *Magnitude, seniors*: unclear *Magnitude, seniors* vs. *non-seniors*: unclear.
Pimentel, Lapane, Briesacher, 2013 [35]; – US Medicare population (65+ years) in long-term care	Findings of prescription drug utilization were mixed. Prescription drug benefit was associated with decreased use of drugs that carry safety concerns, but overall drug utilization may have been unaffected. A shift in drug utilization within drug classes was seen (i.e., from non-covered to covered drugs and utilization of new drugs to treat side effects). *Magnitude, seniors*: unclear. *Magnitude, seniors* vs. *non-seniors*: unclear.
Park, Martin, 2017 [7]; – US Medicare population (65+ years)	Studies consistently found that Medicare Part D increased drug utilization across numerous outcomes, including medication persistence, number of days with possession of at least 1 drug within a class, annual prescription fills per person, drug access, and cost-related behaviour changes such as medication cessation, applying to pharmaceutical assistance programs, and receiving free prescription samples. Similarly, Medicare Part D coverage gaps negatively impacted drug utilization. The coverage gap prompted some substitution of generic for brand-name drugs. *Magnitude, seniors:* the strongest effect sizes were for medication use and increases were highest among beneficiaries receiving low-income subsidies. *Magnitude, seniors* vs. *non-seniors*: unclear.
Ofori-Asenso, Jakhu et al., 2018 [47]; – 65+ years statins users	Higher copayment/cost (not necessarily drug cost-sharing) increased the likelihood of nonadherence and of discontinuation. *Magnitude, seniors*: the association between higher copayment and nonadherence and discontinuation was positive (OR 1.4, 95%CI 1.3, 1.5; OR 1.6, 95%CI 1.5, 1.7). Further interpretation not provided. *Magnitude, seniors* vs. *non-seniors*: unclear.
Mishuk, Fasina, Qian, 2019 [51]; – Individuals affected by US federal and state generic drug policies	Existing evidence evaluating Medicare Part D suggested decreased prescription spending for beneficiaries and increased use of generics. Policies lowering cost-sharing were associated with increased patient’s medication use and adherence, but the impact varied by therapeutic classes while government insurance plans with higher cost-sharing were associated with reduced generic utilization. Evidence suggested that lower cost-sharing increased generic drug use which further enhanced medication adherence. *Magnitude, seniors*: unclear. *Magnitude, seniors* vs. *non-seniors*: unclear.
***Socioeconomic status, chronically ill***	
Lexchin, Grootendorst, 2004 [17]; – The poor and chronically ill	Cost-sharing through the use of copayments or deductibles decreased the use of prescription drugs by the poor and the chronically ill. *Magnitude, poor/chronically ill*: drug price elasticities among vulnerable groups — those with low income and/or chronic illnesses — generally ranged from −0.34 to − 0.50. Some evidence that cost-sharing led to patients foregoing essential medications. *Magnitude, poor/chronically ill*: vs. *non-poor/chronically ill*: unclear.
Goldman, Joyce, Zheng, 2007 [23]; – General population	– Low-income Although studies suggested that low-income beneficiaries reduced drug use with higher copayments, there was little evidence that individuals of lower-income were more sensitive to increased cost-sharing than the general population. *Magnitude, low-income*: same as the general population. *Magnitude, low-income* vs. *non-low-income*: same as the general population. – Chronically ill The evidence suggested that even chronically ill patients were responsive to cost-sharing. *Magnitude, chronically ill*: unclear. *Magnitude, chronically ill* vs. *non-chronically ill*: unclear.
Gemmil, Thomson, Mossialos, 2008 [24]; – General population	Poorer people were not found to be more sensitive to price than the general population. *Magnitude, poor*: among the poor, a 10% increase in price led to reductions in use ranged from 0.3 to 2.0% based on non-aggregate data and 0.5 to 4.0% based on aggregate data. *Magnitude, poor* vs. *non-poor*: among the general population, a 10% increase in price led to a 0.2 to 4.6% decrease in use based on non-aggregate data and a 0.9 to 8.0% decrease in use based on aggregate data.
Remler, Greene, 2009 [25]; – General population	– Low-income Evidence has not consistently shown a relationship between income and cost-sharing effects; the findings were mixed and not conclusive, and the work was limited by the relatively homogenous populations and proxy measures of income. *Magnitude, low-income*: unclear. *Magnitude, low-income* vs. *mid-, high-income*: unclear. – Chronically ill Only a few studies compared the impact of cost-sharing on different health status groups; pharmaceutical cost-sharing among those with chronic disease sometimes reduced the use of valuable drugs; several studies conducted on chronically ill populations (including those with rheumatoid arthritis, heart failure, diabetes, schizophrenia, and lipid disorders) found unambiguous reductions in the use of drugs regarded as important for maintaining the health of the chronically ill. *Magnitude, chronically ill*: unclear. *Magnitude, chronically ill* vs. *non-chronically ill*: unclear.
Holst, 2010 [27]; – General population	– Low-income Some evidence that lower-income individuals were sensitive to increased cost-sharing. *Magnitude, subgroup*: unclear. *Magnitude, low-income* vs. *mid-, high-income*: unclear. – Chronically ill Cost-induced nonadherence to medical recommendations was observed more often among people who needed treatment than among healthy citizens. *Magnitude, chronically ill*: unclear. *Magnitude, chronically ill* vs. *non-chronically ill*: unclear.
Swartz, 2010 [29]; – General population	One study that examined changes in prescription drug copayments imposed on privately insured people indicated that for each medication class examined, individuals living in high-income areas were consistently more likely to continue taking their medications than people in low-income areas after copayments increased. M*agnitude, poor:* unclear. M*agnitude, poor* vs *non-poor:* unclear.
Lemstra, Blackburn et al., 2012 [33]; – Statin users	Statin users required to make a copayment were more likely than others to be nonadherent. *Magnitude, statin users*: among 6 studies with a total sample size of 884,643, patients required to make a copayment when their statin medications were dispensed were 28% more likely than others to be nonadherent (rate ratio 1.3; 95%CI 1.1, 1.5). *Magnitude, statin users* vs. *non-statin users*: unclear.
Maimaris, Paty, et al., 2013 [34]; – Individuals with hypertension	Health insurance and lower cost-sharing were associated with hypertension treatment (defined as the use of at least one antihypertensive medication in an individual with known hypertension) and antihypertensive medication adherence. *Magnitude, individuals with hypertension*: unclear. *Magnitude, individuals with hypertension* vs. *individuals without hypertension*: unclear.
Kiil, Houlberg, 2014 [37]; – General population	The majority of studies found that copayments led to a larger reduction in the use of prescription medicine for vulnerable population groups than for the non-vulnerable general population. *Magnitude, vulnerable population*: unclear. *Magnitude, vulnerable population* vs. *non-vulnerable general population*: unclear.
Mann, Barnieh, et al., 2014 [38]; – Individuals with cardiovascular-related chronic disease	The addition of drug insurance for those without previous drug insurance appear to have consistently increased adherence to medications. In general, studies evaluating drug insurance cost-sharing strategies had conflicting results with some studies showing significant differences in some outcomes while other studies demonstrated no discernible difference in outcomes. The use of deductibles (up to $350 per year) did not appear to have a significant impact on medication adherence. The impact of a maximum out-of-pocket limits was uncertain. *Magnitude, chronically ill*: unclear. *Magnitude, chronically ill* vs. *non-chronically ill*: unclear.
Banerjee, Khandelwal, et al., 2016 [42]; – Individuals with cardiovascular diseases	Reduced copayments and full prescription coverage were associated with increased adherence and persistence. *Magnitude, individuals with cardiovascular diseases*: two retrospective cohort studies investigated the impact of copayments on adherence; 1) among 4105 patients with acute myocardial infarction in Austria, those with waived copayments had higher persistence at 120 days for drug therapy with aspirin, statins, angiotensin-converting enzyme inhibitors (ACEI) or angiotensin-receptor blockers (ARB) than those with copayments (OR 1.4, 95%CI 1.1, 1.7), but β blocker (OR 1.1, 95%CI 0.9, 1.4) or statin use (OR 1.1, 95%CI 0.9, 1.3) did not significantly differ between these groups; 2) a US study of coronary heart disease patients found that compared with copayments <US$10, copayments ≥US$20 were associated with lower persistence at 1 year for statins (OR 0.42; 95%CI 0.36 to 0.49). A US-based RCT included 5855 individuals post-myocardial infarction, randomized to full or usual prescription coverage. Full adherence was higher with full prescription coverage for all medication classes (OR 1.4, 1.2, 1.7). Increased adherence to all three medications for the patient subgroup undergoing coronary artery bypass graft was found, post hoc (OR 1.7, 95% CI 1.04 to 2.7). *Magnitude, individuals with cardiovascular diseases* vs. *individuals without cardiovascular diseases:* unclear.
Doshi, Li et al., 2016 [43]; – Individuals using specialty drugs	– Prescription abandonment (prescription submitted and approved by the insurer but not obtained by the patient): all studies (*n* = 3) reported a strong association of higher cost-sharing with abandonment (vs initiation) of specialty drug prescriptions, for all indications examined. *Magnitude, individuals using specialty drugs*: unclear. – Initiation (first time use of specialty drug within a study period): all studies (*n* = 8) examining initiation in patients with rheumatoid arthritis and multiple sclerosis reported a negative association with higher cost-sharing. Initiation of specialty drugs for cancer was largely reported to be insensitive to cost-sharing in the 3 studies examining this outcome. *Magnitude, individuals using specialty drugs*: the demand elasticity ranged from − 0.03 to − 0.33 for patients with rheumatoid arthritis or multiple sclerosis. – Adherence: evidence on relationship between cost-sharing and adherence was mixed. The majority of studies reported a statistically significant increase in discontinuation associated with increased cost-sharing. *Magnitude, individuals using specialty drugs*: unclear. – Discontinuation/persistence (having a continuous gap of time between prescription fills): 6 of the 7 studies reported a statistically significant increase in discontinuation (or decrease in persistence) associated with increased cost-sharing for at least 1 of the indications examined. *Magnitude, individuals using specialty drugs*: the magnitude of the effects appeared small.
Powell, Saloner, Sabik, 2016 [44]; – Medicaid beneficiaries	Increasing copayments resulted in decreased utilization of drugs and higher rates of non-adherence. However, the magnitude of these associations varied across subgroups. In patients with high need for prescription drugs, studies found that increased copayments resulted in decreased adherence. This was found in Medicaid patients with schizophrenia and privately insured adults with diabetes and congestive heart failure who were living in lowest median income areas. M*agnitude, chronically ill:* unclear. M*agnitude, chronically ill* vs *non-chronically-ill:* unclear. M*agnitude, poor:* unclear. M*agnitude, poor* vs *non-poor:* unclear.
Gourzoulidis, Kourlaba, et al., 2017 [45]; – Individuals with heart failure or diabetes mellitus	7 of 8 studies evaluating the relationship between drug copayment and medication adherence in diabetes mellitus population and 1 of 3 in heart failure population, found a statistically significant inverse association between increases in copayments and medication adherence. *Magnitude, individuals with heart failure or diabetes mellitus*: unclear. *Magnitude, individuals with heart failure or diabetes mellitus* vs. *individuals without heart failure or diabetes mellitus*: unclear.
Gupta, McColl et al., 2018 [46]; – Canadians	Three studies including people with cardiovascular conditions found that those spending ≥5% costs of medications out of their pocket were more likely to report cost-related non-adherence than those spending < 5%. *Magnitude, chronically ill*: unclear. *Magnitude, chronically ill* vs. *non-chronically ill*: unclear.
Schneider, Gaedke et al., 2018 [48]; – Individuals with chronic cardiovascular diseases	Among individuals with chronic cardiovascular diseases, access to insurance or other programs that assisted with medication costs was a protective factor for nonadherence. M*agnitude, chronically ill: i*nsurance or programs that assisted with medication cost was correlated with a 24% decrease in the risk of nonadherence (OR 0.76; 95%CI 0.60, 0.95). *Magnitude, chronically ill* vs. *non-chronically ill*: unclear.
Cheen, Tan et al., 2019 [49]; – Individuals with any of six common chronic diseases.	On the whole, among individuals with chronic diseases (asthma, chronic obstructive pulmonary disease, depression, diabetes mellitus, hyperlipidaemia, hypertension and osteoporosis), higher copayments were associated with primary medication nonadherence. *Magnitude, chronically ill*: a high copayment amount had the strongest association with primary medication nonadherence, with ORs ranging from 1.01 to 33 (compared to lower copayments); *Magnitude, chronically ill* vs. *non-chronically ill*: unclear.

^a^Population examined by each included review

**Table 5:** Results — association between prescription drug insurance/cost-sharing and healthcare services utilization, general population

Authors/year/population	Results — healthcare services utilization, general population
Gibson, Ozminkowsky, Goetzel, 2005 [19];	In most studies, higher levels of prescription drug cost-sharing were not associated with changes in the utilization of low-intensity outpatient medical services, such as physician office visits, outpatient visits, and home health visits. However, these studies assessed small changes in prescription drug cost sharing. Two studies reported an increase in high-intensity health services (such as inpatient visits, emergency department visits, readmissions among older patients hospitalized with complications after acute myocardial infarction) as cost-sharing rose in some diagnostic groups (congestive heart failure or coronary artery disease) while not in others (diabetes mellitus). Four studies reported no association between higher levels of cost-sharing and high-intensity services. *Magnitude:* n/a
Goldman, Joyce, Zheng, 2007 [23];	Increased drug copayments were not associated with more outpatient visits, hospitalizations, or emergency department visits among a broader population (not restricted to the elderly or those with chronic conditions). *Magnitude:* n/a
Gemmil, Thomson, Mossialos, 2008 [24];	There was generally a positive relationship between prescription drug cost-sharing and outpatient, inpatient, and emergency care. Studies also found that prescription limits increased the frequency of partial hospitalization and nursing home admissions and the use of emergency mental health service. Two studies that found no effect were based on chronically ill patients. *Magnitude:* unclear.
Remler, Greene, 2009 [25];	Some evidence suggested that pharmaceutical cost-sharing increased emergency department use and hospitalizations; limited evidence about resulting increases in outpatient care. *Magnitude:* unclear.
Green, Maclure, et al., 2010 [26];	The effects of pharmaceutical reimbursement on health care access were uncertain. Some studies reported an immediate increase in utilization while another found no significant difference in office visits, hospitalization, or length of stay. Very few studies looked at the long-term impact on utilization. One study reported an increase in outpatient services but no change in inpatient and long-term services. *Magnitude:* unclear.
Eaddy, Cook, et al., 2012 [32];	Most studies indicated that increased patient drug cost-sharing adversely affected health services utilization (emergency department visits, outpatient visits, preventative services, hospitalizations and nursing-home admissions). Fewer studies indicated that an increase in cost-sharing did not affect medical utilization or number of medical visits. *Magnitude:* unclear.
Kiil, Houlberg, 2014 [37];	Overall, pharmaceutical copayments had positive effects on the substitution to other types of health care services (such as hospitalization, accident emergency departments, long-term care, general practise consultation); *Magnitude:* unclear
Kesselheim, Huybrechts et al., 2015 [39];	Multiple studies found that limiting drug insurance was associated with an increase in the use of health services including emergency department use, hospitalizations, nursing home admissions, psychiatric hospitalizations, outpatient mental health visits, and emergency mental health services; other studies found that the expansion of drug insurance led to reductions in hospitalizations. *Magnitude:* one study reported the effect of reaching the coverage limit in Medicare Part D on emergency department use and hospitalizations (RR 1.6; 95%CI 1.4, 1.8; RR 1.9, 95%CI 1.6, 2.1). Another study reported positive associations between reaching the Part D coverage gap and worse outcomes among patients in psychiatric institutions with schizophrenia and bipolar disorder, including hospitalizations (schizophrenia: HR 1.3; 99.5%CI 1.1, 1.7; bipolar disorder: HR 1.3; 99.5%CI 1.0, 1.6).
Luiza, Chavez et al., 2015 [40];	The effects of pharmaceutical cost-sharing on emergency department use, hospitalization or use of outpatient care were uncertain. *Magnitude:* unclear.
Gupta, McColl et al., 2018 [46]; – Canadians	Evidence regarding the impact of cost-related nonadherence on individual health outcomes such as disease exacerbation, poor self-reported health, increase in symptoms leading to increasing hospitalizations, emergency department visits, or mortality was limited and mixed. Two studies found that relative to those with no drug insurance, the insured made more use of physician services. *Magnitude:* unclear.
Kolasa, Kowalcyzk, 2019 [50];	All 11 included studies found positive associations between increases in out-of-pocket expenses for drugs and the use of health care services (9 of 11 found associations that were statistically significant). Health care services included physician visits, hospitalization, and emergency room visits. *Magnitude:* unclear.

**Table 6:** Results — association between prescription drug insurance/cost-sharing and healthcare services utilization, for/between specific populations

Authors/year/population^**a**^	Results — healthcare services utilization, for/between specific populations
***Older adults, seniors***	
Adams, Soumerai, Ross-Degnan, 2001 [15]; – US Medicare population (65+ years)	The association between prescription drug insurance/cost-sharing and healthcare services utilization were not explicitly discussed. In the New Hampshire drug cap studies, an increase in nursing home admissions for chronically ill elderly persons was affected by the cap. Hospitalizations during the period of the cap also increased but the difference was not statistically significant. For patients with schizophrenia, use of emergency mental health services and partial hospitalization during the time of the cap increased, and then decreased to near pre-cap levels after the cap was repealed; *Magnitude, elderly: e*lderly Medicaid enrolees in New Hampshire were almost twice as likely to be admitted to nursing homes during the period of the cap as those in New Jersey (RR 1.8; 95%CI 1.2, 2.6). In addition, there was a slight trend toward higher rates of hospitalization in the New Hampshire cohort during the period of the cap, but this difference was not statistically significant (RR 1.2; 95%CI 0.8, 1.6). For patients with schizophrenia, use of emergency mental health services and partial hospitalization during the time of the cap increased by 57%. *Magnitude, elderly* vs. *non-elderly:* unclear.
Rice, Matsuoka, 2004 [18]; – Seniors	Results were contradictory and not conclusive for hospitalization and long-term care admission rates in response to cost-sharing or prescription drug payment limits. However, having some form of supplemental insurance was associated with more appropriate health care use, particularly when such supplemental insurance provided coverage for prescription medication. *Magnitude, elderly:* unclear. *Magnitude, elderly* vs. *non-elderly:* unclear.
Maio, Pizzi, Roumm, 2005 [20]; – Seniors	For seniors, prescription drug cost-sharing and the use of caps may have led to greater risk of hospitalization or admittance to nursing home facilities. *Magnitude, elderly:* unclear. *Magnitude, elderly* vs. *non-elderly:* unclear.
Swartz, 2010 [29]; – General population	Increased cost-sharing for prescription drugs appeared to cause increased expenditures on emergency department services and inpatient hospitalizations by elderly and welfare beneficiaries. M*agnitude, elderly:* unclear. M*agnitude elderly* vs *non-elderly*: unclear.
***Socioeconomic status, chronically ill***	
Lexchin, Grootendorst, 2004 [17]; – The poor and chronically ill	Some evidence that prescription drug cost-sharing led to increases in use of emergency services (acute care hospitalization, emergency room admission, long-term care admission), and nursing home admissions. *Magnitude, poor/chronically ill:* unclear. *Magnitude, poor/chronically ill* vs. *non-poor/chronically ill:* unclear.
Goldman, Joyce, Zheng, 2007 [23]; – General population	The findings from studies focusing solely on chronically ill patients were unambiguous: for patients with congestive heart failure, lipid disorders, diabetes, and schizophrenia, greater use of inpatient and emergency medical services was associated with higher cost-sharing for prescription drugs. For certain conditions, the evidence clearly indicated that more cost-sharing was associated with increased use of other medical services, such as hospitalizations and emergency department visits. *Magnitude, chronically ill:* unclear *Magnitude, chronically ill* vs. *non-chronically ill:* unclear
Remler, Greene, 2009 [25]; – General population	Some studies examined a selective reduction in cost-sharing for selected important chronic medications and found significant increases in their use that might be associated with significant reductions in emergency room and hospital usage. Some evidence suggests that pharmaceutical cost-sharing increased emergency department use and hospitalizations. There was less evidence about increases in outpatient care. *Magnitude, chronically ill:* unclear. *Magnitude, chronically ill* vs. *non-chronically ill:* unclear.
Holst, 2010 [27]; – General population	With certain chronic conditions, an increase in drug copayments led to increased use of other medical services such as consulting practitioners and hospital admissions. *Magnitude, chronically ill:* unclear. *Magnitude, chronically ill* vs. *non-chronically ill:* unclear.
Swartz, 2010 [29]; – General population	One study argued that the evidence was unambiguous for people with chronic illnesses that higher cost-sharing led to greater use of hospital inpatient and emergency department services. Low-income people in poor health were more likely to suffer adverse outcomes, such as increased rates of emergency department use, hospitalizations, admission to nursing homes when increased cost-sharing caused them to reduce their use of health care, particularly prescription drugs. M*agnitude, chronically ill:* unclear. M*agnitude, chronically ill* vs *non-chronically ill:* unclear. M*agnitude, low income:* unclear. M*agnitude, low income* vs *high income:* unclear.
Powell, Saloner, Sabik, 2016 [44]; – Medicaid beneficiaries	Reduced use of prescription drugs from nonadherence has been linked to adverse consequences. A study of Medicaid beneficiaries with cancer found that after relatively small copayments were imposed ($0.50–$3.00) in Georgia in 2002, days supply of medication decreased and odds of an ED visit increased. Outside Medicaid, there is strong evidence from a natural experiment in Québec where increased copayments for prescription drugs led to a spike in hospitalizations. M*agnitude, chronically ill:* unclear. M*agnitude, chronically ill* vs *non-chronically-ill:* unclear.
Gourzoulidis, Kourlaba, et al., 2017 [45]; – Individuals with heart failure or diabetes mellitus	Studies showed no significant association between copayment change and emergency department visits, office visits, hospitalizations or laboratory/diagnostic tests among patients with diabetes mellitus. One study found that higher drug copayments were associated with an increase in emergency department visits among patients with heart failure. *Magnitude, chronically ill:* unclear. *Magnitude, chronically ill* vs. *non-chronically ill:* unclear.
Gupta, McColl et al., 2018 [46]; – Canadians (elderly, chronically ill, poor)	A study conducted with elderly and social assistance recipients in Québec found that the introduction of cost-sharing was associated with increased rates of emergency department visits. Another study found that among elderly patients with rheumatoid arthritis, higher cost-sharing was associated with more physician visits and among those were admitted to the hospital at least once, there were more admissions. *Magnitude, poor/chronically ill:* unclear. *Magnitude, poor/chronically ill* vs. *non-poor/chronically ill:* unclear.
***Children***	
Mishuk, Fasina, Qian, 2019 [51]; – individuals affected by US federal and state generic drug policies	Government insurance plans with high-cost sharing on generic drugs were associated with less use of health services among children. *Magnitude, children:* unclear. *Magnitude, children* vs. *adults:* unclear.

^a^Population examined by each included review

**Table 7:** Results — association between prescription drug insurance/cost-sharing and health outcomes, general population

Authors/year/population	Results –– health outcomes, general population
Harten, Ballantyne, 2004 [16]; – Canadians	Only one included study examined health outcomes. It found that drug cost-sharing was associated with a decrease in essential drugs, which was associated with an increase in adverse events as measured by hospitalization, nursing home admissions and mortality (in seniors and welfare recipients); *Magnitude:* unclear.
Gibson, Ozminkowsky, Goetzel, 2005 [19];	No studies were identified that measured the effects of prescription drug cost-sharing on direct measures of health status, such as self-reported health status and empirical measures of clinical health status (e.g., laboratory readings). One study found that higher levels of cost-sharing had no effect on mortality rates while another reported an indirect decline in claims-based score of health status because of a copayment increase from $1 to $3 but not when there there was a copayment increase from 50% with a $25 maximum to 70% with a $30 maximum. *Magnitude:* unclear.
Goldman, Joyce, Zheng, 2007 [23];	The direct evidence on the link between prescription drug cost-sharing and health was limited. Most studies found that when the population was not limited to those with certain chronic illnesses, the outcomes associated with prescription drug cost-sharing were mostly benign. Studies that looked at cost-sharing effects more broadly were ambiguous in their findings. *Magnitude:* unclear.
Gemmil, Thomson, Mossialos, 2008 [24]	Overall, most studies that directly or indirectly considered the impact of prescription drug charges on health concluded that they lowered or were likely to lower health status because they led patients to forego the use of essential drugs, reduced adherence to treatment, and increased the likelihood of needing more intensive care and of dying. *Magnitude of effect:* unclear.
Remler, Greene, 2009 [25];	There was a lack of direct evidence about pharmaceutical cost-sharing’s effect on health. *Magnitude:* n/a
Green, Maclure, et al., 2010 [26];	Only two of the studies included reported health outcome data, precluding any conclusions about the impact of prior authorization policies on patient outcomes. *Magnitude:* n/a
Swartz, 2010 [29];	Very few studies looked at the effect of cost-sharing on health. As such, long-term health effects of reduced use of essential drugs especially people with chronic health conditions is unknown. M*agnitude:* n/a
Baicker, Goldman, 2011 [30];	Evidence on the ultimate effect of cost-sharing on health outcomes was sparse. Most studies did not examine the effect of coinsurance on health directly. Existing evidence suggested that increased out-of-pocket costs led to lower compliance of drug use, which may indirectly have led to poorer health. *Magnitude:* unclear
Eaddy, Cook, et al., 2012 [32];	Most studies indicated that increased patient drug cost-sharing adversely affected health outcomes (outcomes included adverse events, self-reported health status, and symptoms). A few studies found no effect on outcomes and no effect of adherence, supporting the hypothesis that the effect of cost-sharing on outcomes is mediated through adherence; *Magnitude:* unclear.
Kiil, Houlberg, 2014 [37];	Overall, the effects of copayments on mortality was unclear. The health effects of copayment have only been analyzed empirically in a limited number of studies, of which half did not find any significant effects in the short-term. Some studies observed a drop in the use of essential medicines following an increase in copayment which led to an increase in mortality, while increased drug compliance because of a drop in copayment reduced rate of mortality. . *Magnitude:* unclear.
Kesselheim, Huybrechts et al., 2015 [39];	- 6 studies evaluated the impact of drug insurance on patients’ health by comparing cohorts of patients with and without coverage. 4 of 6 studies found that those with insurance had better treatment adherence and/or health outcomes (self-reported health, mortality, functional disability, hospitalizations); - 5 studies examined the effects of extending drug coverage to patients on their health outcomes; findings were mixed; - 5 studies evaluated the effects of drug insurance restrictions on health outcomes; 4 of 5 studies found that drug insurance restrictions led to worse treatment adherence and health outcomes (emergency department use, hospitalizations, health outcomes, rates of death). *Magnitude:* unclear.
Gupta, McColl et al., 2018 [46]; – Canadians	Few studies reported that cost-sharing for drugs in the form of copayments led patients to forego essential medications and a decline in health care status. *Magnitude:* unclear.
Kolasa, Kowalcyzk, 2019 [50];	Association between drug cost-sharing and health outcomes was reported in 7 studies, of which 5 found statistically significant results of an inverse relationship. 6 studies studied a direct relationship while 1 studied an indirect relationship through adherence. Health outcomes included self-assessed health, major vascular events, cardiovascular-related mortality and all-cause mortality. *Magnitude:* unclear

**Table 8:** Results — association between prescription drug insurance/cost-sharing and health outcomes, for/between specific population

Authors/year/population^**a**^	Results — health outcomes, for/between specific population
***Older adults, seniors***	
Adams, Soumerai, Ross-Degnan, 2001 [15]; – US Medicare population (65+ years)	Some evidence that cost-sharing and limits on the the number of reimbursable prescriptions led to serious adverse health outcomes for sick and low-income Medicare beneficiaries (nursing home admissions, use of clinic emergency mental health services by schizophrenic patients). *Magnitude, seniors:* unclear. *Magnitude, seniors* vs. *non-seniors:* unclear.
Rice, Matsuoka, 2004 [18]; – Seniors	Among seniors, cost-sharing (not necessarily drug) resulted in lower health status (either higher mortality or various measures of morbidity), with the following two notable exceptions: 1) when generous provisions were in place to protect vulnerable populations from incurring undue financial risk as a result of cost sharing, 2) the case of patients experiencing serious medical events because they realize the necessity of receiving recommended medical care irrespective of cost-sharing requirements. *Magnitude, seniors:* unclear. *Magnitude, seniors* vs. *non-seniors:* unclear.
Maio, Pizzi, Roumm, 2005 [20]; – Seniors	There was mixed evidence that prescription drug cost-sharing mechanisms (copayment, coinsurance, and deductible) had negative effects on seniors’ health. *Magnitude, seniors:* unclear. *Magnitude, seniors* vs. *non-seniors:* unclear.
Pimentel, Lapane, Briesacher, 2013 [35]; – US Medicare population (65+ years) in long-term care	Results were overall inconsistent. Clinician reports suggested a high incidence of adverse events (e.g., psychiatric hospital admissions, emergency department visits) immediately following medicare prescription drug plan and adverse effects of prescription drug substitutions for formulary-related reasons, however, some long-term care providers did not perceive adverse health effects of Part D among residents. *Magnitude, seniors:* unclear. *Magnitude, seniors* vs. *non-seniors:* unclear.
***Socioeconomic status, chronically ill***	
Lexchin, Grootendorst, 2004 [17]; – The poor and chronically ill	Some evidence that drug cost-sharing led to increases in serious adverse events (defined as the first occurrence of acute care hospitalization, long-term care admission, or death; nursing home admission, use of emergency mental health services among those with schizophrenia). *Magnitude, poor/chronically ill:* unclear. *Magnitude, poor/chronically ill* vs. *non-poor/chronically ill:* unclear.
Goldman, Joyce, Zheng, 2007 [23]; – General population	Some studies found that higher cost-sharing was associated with adverse outcomes especially among vulnerable populations such as the elderly and poor. *Magnitude, poor/chronically ill:* unclear *Magnitude, poor/chronically ill* vs. *non-poor/chronically ill:* unclear.
Swartz, 2010 [29]; – General population	Low-income people were at greater risk than higher income people in terms of poor health outcomes due to increased cost-sharing. *magnitude, low income:* unclear. *magnitude, low income* vs *high income:* unclear.
Baicker, Goldman, 2011 [30]; – General population	Adverse health consequences of cost-sharing (unclear if drug cost-sharing only) have been found for patients with congestive heart failure, lipid disorders, diabetes, and schizophrenia. *Magnitude, chronically ill:* unclear *Magnitude, chronically ill* vs. *non-chronically ill:* unclear.
Maimaris, Paty, et al., 2013 [34]; – Individuals with hypertension	Health insurance and lower cost-sharing were associated with hypertension awareness and hypertension control in individuals being treated for hypertension, or, alternatively, measured by the mean blood pressure amongst individuals with hypertension. *Magnitude, individuals with hypertension:* unclear. *Magnitude, individuals with hypertension* vs. *individuals without hypertension:* unclear.
Mann, Barnieh, et al., 2014 [38]; – Individuals with cardiovascular-related chronic diseases	Results for clinical outcomes were scarce and mixed (only 2 studies were identified). *Magnitude, individuals with cardiovascular-related chronic disease: u*nclear. *Magnitude, individuals with cardiovascular-related chronic diseases* vs. *individuals without cardiovascular-related chronic diseases:* unclear.
Gourzoulidis, Kourlaba, et al., 2017 [45]; – Individuals with heart failure or diabetes mellitus	Only 1 included study examined the association between changes in copayments and health outcomes; higher copayments were associated with poorer glycemic control. *Magnitude, individuals with heart failure or diabetes mellitus:* each $5 increase in patient drug cost share resulted in a 0.1% point increase in glycosylated hemoglobinA(1c). *Magnitude, individuals with heart failure or diabetes mellitus* vs. *individuals without heart failure or diabetes mellitus:* unclear.

^a^Population examined by each included review

### Associations of prescription drug insurance and cost-sharing with drug utilization

Examining the 20 reviews that investigated, with a focus on the general population, the association between having prescription drug coverage or varying levels of cost-sharing on drug use, there was a clear inverse association, but the magnitude of the association was unclear (Tables 2 and 3). Across the literature, the outcomes were reported in elasticities, changes in drug use, and changes in medication adherence, with reviews published between 2004 and 2019. Reviews assessing medication adherence generally found that the absence of prescription drug insurance, or having copayments, reduced medication adherence [21, 32, 36, 41, 46] with specific estimates ranging as low as a 0.4% decrease in adherence for each dollar increase in copays, and an average reduction of 3% after 1 year of copayment reductions [32]. Another review reported that publicly insured patients who were required to pay copays for their prescription medicines had 11% higher odds of reporting nonadherence relative to those who faced no copayments [36]. However, not all associations were statistically significant, with variations in adherence reported depending on the drug class. Reviews reporting on drug use also generally found consistent results, reporting that increasing cost-sharing or not having drug insurance decreased drug use, but with varying impacts by drug class or type, and some not reporting clear effect sizes or very small to moderate impacts [16, 26, 27, 37, 39, 40, 51]. Own-price elasticities reported in seven older reviews, published in 2011 or earlier, generally found that the demand for prescription drugs was inelastic, with most estimates ranging from − 0.2 to − 0.6 depending on the drug class and essentiality, suggesting that a 10% increase in price resulted in a 2 to 6% decrease in use [19, 22–25, 29, 30].

#### Differences between subgroups: SES, health status, age, and sex/gender

These results varied when assessing vulnerable population subgroups including the elderly, children, the poor, and the chronically ill (Tables 2 and 4). We identified two reviews that focused specifically on low-income groups [17, 44] and six that generally commented on low-SES populations [23–25, 27, 29, 37]. One older review focusing on low-income populations reported price elasticities ranging from − 0.3 and − 0.5 and argued there was unequivocal evidence that increasing cost-sharing decreased drug use among the poor [17]. This conclusion was supported by all other reviews except one [25]. However, when comparing price responsiveness between the poor and non-poor, four reviews provided mixed or no evidence that individuals with lower income were more price sensitive than those with higher income [23–25, 27]. Although one review concluded that higher copayments led to a greater reduction in drug use in vulnerable populations (low socioeconomic status measured by income, education, or social status) than the non-vulnerable population) [37].

With respect to the chronically ill, reviews generally concluded that higher copayments or the absence of drug insurance were associated with increased medication nonadherence for a range of illnesses and drug classes [17, 23, 25, 27, 33, 34, 38, 42–49]. However, the magnitude of effects was often unclear and difficult to synthesize given the diverse outcome measures employed in each review. A recent meta-analysis focusing on individuals with chronic cardiovascular diseases found that access to insurance or other programs that assisted with medication costs resulted in a 37% decrease in the risk of medication nonadherence [48]. Another recent review examined the association between cost-sharing on specialty drugs for rheumatoid arthritis, multiple sclerosis, and cancer, and the use of specialty drugs and nondrug medical services, and health outcomes [43]. Although no research was found that pertained to the use of nondrug medical services and health outcomes, the review found that higher cost-sharing was associated with higher prescription abandonment and discontinuation/persistence, and lower initiation and adherence. Findings varied by diseases but generally indicated stronger effects for noninitiation or abandonment of a prescription at the pharmacy and somewhat smaller effects for refill behaviour once patients initiated therapy [43]. Lastly, we are unable to comment on how the impact of cost-sharing or drug insurance on drug use compared to drug use by the healthy population as such comparisons were not drawn in the literature.

The elderly was the most studied group apart from the chronically ill, with 14 reviews focusing on this particular population. Of the 14 reviews, 11 concluded that seniors were sensitive to price changes [7, 15, 18, 21, 27–31, 47, 51]; drug use decreased with increasing cost-sharing or the lack of drug insurance, with the others finding mixed or no evidence for price responsiveness among older adults [20, 24, 35]. Again, the magnitude of effect was difficult to evaluate, and we are unable to comment on age differences (elderly vs non-elderly) in price responsiveness.

Only two reviews mentioned potential sex/gender differences in responsiveness to changes in cost-sharing or insurance. One review reported that one study had found that a drug policy change had not reduced the use of essential cardiac medications among Québec elderly who experienced acute myocardial infarction and that this finding did not vary by sex [23]. Another review reported that one study had found that low-income single elderly women were much less price responsive to drug fees than low-income single elderly men in British Columbia [17].

### Associations of prescription drug insurance and cost-sharing with health services use

On the whole, most reviews concluded that increasing prescription drug cost-sharing or limiting drug insurance were associated with higher healthcare services utilization, such as emergency room visits, hospitalizations, and outpatient visits in the general population, although the magnitudes of associations were unclear (Tables 2 and 5) [24, 25, 30, 32, 37, 39, 50]. Two older reviews (published in 2005 and 2007) found no evidence of associations between prescription drug cost-sharing and changes in the use of healthcare services such as outpatient visits or hospitalizations [19, 23] while three relatively more recent reviews from 2010, 2015, and 2018 concluded the evidence was mixed or uncertain [26, 40, 46].

#### Differences between subgroups: SES, health status, age, and sex/gender

When assessing results by subgroups, the findings were generally the same as those reported in the general population (Tables 2 and 6). Three reviews that focused on both the poor and chronically ill found that, in most studies reviewed, drug cost-sharing was associated with increased emergency department visits, hospitalizations, and nursing home admissions [17, 29, 44]. The magnitude of these associations was, however, unclear. A more recent review of cost-related nonadherence to prescription medications in Canada provides further support and reported that, among the elderly and individuals on social assistance, the introduction of cost-sharing was associated with increased rates of emergency department and physician visits [46]. It was unclear, however, if any of these associations differed in magnitude when compared to healthier or higher-income populations.

Five reviews specifically discussed the association between prescription drug cost-sharing and healthcare services utilization in the chronically ill [23, 25, 27, 45, 46]. Four of these reviews found evidence that prescription drug cost-sharing was associated with increased use of health services including greater hospitalizations, emergency room visits, and nursing home admissions [23, 25, 27, 46]. The magnitude of these associations was, however, unclear. One review concluded that there was ‘no strong’ evidence showing a direct association between drug cost-sharing and healthcare services use among patients with diabetes mellitus, although there was limited evidence that higher drug copayments were associated with an increased risk of hospitalization among patients with heart failure [45]. Nonetheless, it was unclear how chronically ill patients compared to the healthier population in terms of the association between drug-cost sharing and healthcare services use.

Five reviews examined the association between drug cost-sharing or drug insurance and healthcare services utilization in older adults [15, 18, 20, 29, 46]. Four of these reviews concluded that there was some evidence that higher drug cost-sharing and lack of insurance were associated with greater hospitalizations or nursing home admissions in seniors, although the magnitude was unclear, whereas one older review reported inconclusive findings [18]. It was also unclear how seniors compared to non-seniors with respect to healthcare service utilization when faced with drug cost-sharing. Lastly, one recent review reporting on the association between drug cost-sharing and health services use found that government insurance plans with high-cost sharing on generic drugs were associated with lower use of health services among children. Again, the magnitude of effect was unclear and no comparison was drawn with older individuals [51].

Only one review mentioned potential sex/gender differences in responsiveness to changes in cost-sharing or insurance. One review reported that one study had found that a drug policy change had not reduced the use of medical services among Québec elderly who experienced acute myocardial infarction and that this finding did not vary by sex [23].

### Associations of prescription drug insurance and cost-sharing with health

A total of 21 reviews reported on the association between prescription drug insurance or cost-sharing and health outcomes (Tables 2 and 7). Eleven of these reviews explored the association in the general population [19, 23–26, 29, 30, 32, 37, 39, 50] of which two focused specifically on the Canadian population [16, 46]. Six reviews examined health generally [23, 25, 26, 29, 30, 46], five all-cause mortality [16, 24, 37, 39, 50], four self-reported health [19, 32, 39, 50] and one review investigated cardiovascular-related mortality [50], adverse events [32] and vascular events [50].

Overall, there was limited evidence of a clear relationship between prescription drug insurance or cost-sharing and health outcomes. With one exception [32], several older reviews reported that very few empirical studies had examined the association between drug insurance/cost-sharing and health, and concluded that, on the whole, existing studies provided mixed or unclear evidence [19, 23, 25, 26, 29, 30, 37]. More recent reviews (published in 2015 and 2019) tended to conclude that drug insurance and lower cost-sharing were associated with better health. One review found that individuals with drug insurance had better health outcomes than those without, that drug insurance restrictions led to a decline in health status, and that extending drug coverage yielded mixed results [39]. Another review found that in all included studies, there was an inverse association between higher drug cost-sharing and health outcomes such as self-assessed health, major vascular events, cardiovascular-related mortality and all-cause mortality [50]. The above conclusions were, however, all based on very few primary studies.

#### Differences between subgroups: SES, health status, age and sex/gender

Reviews highlighted a paucity of studies that examined the associations of prescription drug insurance and cost-sharing with health among the poor and the chronically ill (Tables 2 and 8). Two older reviews found some evidence that drug cost-sharing was associated with adverse health outcomes in lower-income populations and another suggested that low-income individuals were at greater risk of poor health outcomes due to increased cost-sharing than higher-income individuals [17, 23, 29]. Three of four reviews that specifically discussed the chronically ill found that cost-sharing was associated with adverse health outcomes in patients with heart disease, hypertension, lipid disorders, and diabetes [30, 34, 45]. Two of the three reviews, however, discussed the association between health insurance generally (i.e., including but not limited to drug insurance) and health outcomes [30, 34]. One review found no evidence of an association between drug cost-sharing and clinical outcomes among patients with cardiovascular-related chronic disease [38]. Four older reviews specifically focused on the association between insurance and cost-sharing and health among seniors [15, 18, 20, 35]. Two reviews reported mixed findings [20, 35] while two reviews reported that higher cost-sharing was associated with worse health outcomes, including higher mortality and morbidity among seniors [15, 18]. One review pointed out that this association did not remain when there were generous provisions in place to protect vulnerable populations from incurring undue financial risk as a result of cost-sharing [18]. However, similar to previous reported outcomes, no comparisons were drawn between the poor and non-poor, the chronically ill and non-chronically ill, and the elderly and non-elderly and how health outcomes may have differed between them. We did not identify a single review that discussed potential differences between sex/gender in the association of prescription drug insurance and cost-sharing with health.

### Risk of bias assessme﻿nt

In our umbrella review, we found that the most common limitations were the lack of an a priori study design and issues with clarity in reporting search strategies and results. Reviews often did not clearly report data screening and extraction procedures including exclusion and inclusion criteria, had poorly described search strategies or non-systematic search strategies, failed to provide or clearly synthesize study characteristics and, most often than not, did not provide a list of excluded studies. The most important limitation was, however, the lack of attention given to quality assessments. About half of the included reviews did not conduct any formal quality assessments and many that did often failed to appropriately describe and justify their quality assessment.

## Discussion

### Main findings

We found consistent evidence that changes in drug cost-sharing and/or drug insurance were associated with drug use. Lower cost-sharing and having drug insurance were associated with increased drug use while higher drug cost-sharing and the lack or loss of drug insurance were associated with decreased drug use. We also found consistent evidence that the poor, the chronically ill, seniors and children were similarly responsive to changes in insurance and cost-sharing. Although the direction of the associations between changes in drug insurance and cost-sharing was clear, the magnitude of these associations was difficult to ascertain. The demand for prescription drugs is most certainly inelastic (i.e., a percentage change in price is associated with a smaller percentage change in demand) with an own-price elasticity ranging from about − 0.2 to − 0.6, depending on drug class, intervention, disease, and population studied. We found that lower drug cost-sharing and drug insurance were associated with lower healthcare services utilization including emergency room visits, hospitalizations, and outpatient visits. Similar results were found in all population subgroups aside from children, although the literature on the poor and children was very limited. We did not find consistent evidence of an association between cost-sharing and insurance and health. While several reviews reported mixed or no evidence, more recent reviews tended to conclude that there was some evidence that increased cost-sharing led to poorer health outcomes because of reduced drug adherence. Again, the magnitude of effect was unclear and evidence on the elderly, chronically, ill, and poor was limited and mixed. Lastly, we did not find any evidence that the association between drug insurance or cost-sharing and drug use, health services use, or health differed by SES, health status, age or sex.

We found two reviews that specifically studied the Canadian population. An older review examined Canadian evidence of the effects of cost-sharing mechanisms of provincial drug benefit programs on drug utilization and health [16]. A more recent scoping review examined the extent, determinants, and consequences of cost-related nonadherence to prescription medications in Canada [46]. The two reviews generally found that higher drug cost-sharing reduced drug use. There was, however, little discussion of the magnitude of associations or subgroup differences in price responsiveness [16, 46]. The review of cost-related nonadherence to prescription medications found limited and mixed evidence that cost-sharing increased health services use [46]. A more recent review examined the prevalence, predictors, and clinical impact of cost-related medication nonadherence in Canada [52]. Along with lower income, younger age, and poorer health, high out-of-pocket spending and drug insurance were found to be associated with medication cost-related nonadherence [52].

### Limitations

Our review has some inherent limitations. Although we identified 38 relevant reviews, this does not equate to 38 independent reviews because there was considerable overlap between the studies that were included in the reviews. Although we are confident about the direction of the associations we examined, we had difficulties commenting on the precise magnitude of associations as these were often not clearly identified and reported in the reviews themselves, and could not be easily extracted and synthesized. Lastly, our review did not examine reviews that focused specifically on an alternative cost-sharing design called “value-based cost-sharing” or more generally “value-based insurance design.” The key feature of value-based insurance design is to link the amount of cost-sharing across services with the documented effectiveness and cost-effectiveness of a service, drug or device. A list of reviews that focused specifically on value-based designs is provided in the Additional file 1.

### Implications for research

Our umbrella review highlights a paucity of research focused on children and youth. We identified no reviews that specifically focused on children and youth. The reviews we included generally sparingly discussed the potential impact of drug insurance and cost-sharing among youth. In our search, we identified a single review that focused specifically on children, which we excluded because it focused primarily on access and not on drug use. Unger and Ariely, identified two studies that compared insured and uninsured paediatric populations which showed increased access to healthcare services and medications for insured children [53]. The review noted that access to prescription drugs frequently differed by the type of health insurance provider and the type of cost-sharing arrangement and that more research was needed. The lack of discussion of potential sex/gender differences in the associations of prescription drug insurance and cost-sharing with drug use, health services use, and health is of concern. Only two reviews discussed this issue and reported on just two primary studies. It is unclear if the lack of discussion of potential sex/gender differences is due to reviews or primary studies not investigating it.

Future reviews need to give more consideration to appropriately synthesizing and discussing magnitudes of effect for given associations as solely presenting the direction or significance of a relationship provides minimal information. A stronger emphasis also needs to be placed on improving the methodological rigour of reviews by employing systematic and transparent methods to develop and execute search strategies as well as conducting quality assessment that is applicable to the literature being reviewed and ensuring that it is adequately discussed. Lastly, our umbrella review highlights the importance of searching systematically both peer-reviewed and grey literature, and not to overly rely on a single repository of research evidence. For example, only 11 reviews are included in Health Systems Evidence, which is perhaps the most comprehensive repository of reviews relevant to health systems.

## Conclusions

### Implications for health equity

Socioeconomic, racial and ethnic inequities in health care and drug coverage are well documented in the US and Canada [1, 54, 55]. For example, in 2015–16 in Canada, relative to adults in the lowest income decile, those in the 10th decile had odds of reporting drug insurance coverage that were more than five times higher [54]. In the US, Black and Latinx/Hispanic adults have historically reported substantially higher uninsured rates than white adults. In 2019, while the uninsured rate among white adults was only 9%, the uninsured rates among Black and Latinx/Hispanic adults stood at 14 and 26%, respectively. Consequently, universal pharmacare would likely increase drug use among lower-income populations relative to higher-income populations, and potentially reduce health inequities.

### Implications for policy

Although cost-sharing can be used as a mechanism to reduce pharmaceutical expenditures, the associated impacts on health service use may offset those benefits. These cross-price effects of extending drug coverage are, however, often ignored in costing simulation, [56, 57] and need to be taken into consideration by policymakers. Lastly, current Canadian universal pharmacare proposed designs most often include cost-sharing for all but the most vulnerable despite evidence that cost-sharing reduces drug use and treatment adherence, and likely results in increases in health services use [3, 58].

## Supplementary Information

**Additional file 1: Appendix A.** Search strategy. **Appendix B.** Quality Assessment / Risk of Bias tools. **Appendix C.** Characteristics of included studies. **Appendix D.** Excluded studies. **Appendix E.** List of Canadian studies included in reviews. **Appendix F.** List of reviews that focused specifically on value-based cost-sharing/insurance design.

## Data Availability

All data generated or analyzed during this study are included in this published article [and its supplementary information files].

## Abbreviations

ACEI: Angiotensin-converting enzyme inhibitors
AMSTAR: Assessment of Multiple Systematic Reviews
ARB: Angiotensin-receptor blockers
CI: Confidence Intervals
ED: Emergency Department
EPHPP: Effective Public Health Practice Project
EPOC: Effective Practice and Organisation of Care
GRADE: Grading of Recommendations, Assessment, Development and Evaluations
HR: Hazard Ratio
ITS: Interrupted Time Series
NIH: National Institute of Health
OR: Odds Ratio
RCT: Randomized Controlled Trial
RePEc: Research Papers in Economics
RM: Repeated Measures
RR: Relative Risk
SES: Socioeconomic Status
STROBE: Strengthening the Reporting of Observational Studies in Epidemiology
US: United States
