# Supplementary material for: A systematic umbrella review of the association of prescription drug insurance and cost-sharing with drug use, health services use, and health
Source: BMC Health Serv Res. 2022 Mar 3;22:297. doi: 10.1186/s12913-022-07554-w (PMC8895849; doi:10.1186/s12913-022-07554-w)
Supplement: Supplementary file 1 — Additional file 1: Appendix A. Search strategy. Appendix B. Quality Assessment / Risk of Bias tools. Appendix C. Characteristics of included studies. Appendix D. Excluded studies. Appendix E. List of Canadian studies included in reviews. Appendix F. List of reviews that focused specifically on value-based cost-sharing/insurance design. [file 12913_2022_7554_MOESM1_ESM.pdf]

Appendix A. Search strategy

Date of initial search: 22 June 2017
Dates of updated searches: 15 September 2019 and 14 September 2020

EconLit

1. SU health insurance OR SU (costs and fees) OR SU health policy OR SU uninsured OR SU national health insurance
2. TI ((Cost or cost sharing or deductible or coinsurance or co insurance or benefit plan or capitation or cash payment or charge or fee or direct payment or direct contribution or pocket or expense or copay or co payment or co-pay or capitation or tier or tiered system or multitier or onetier or twotier or threetier or single pay)) OR AB ((Cost or cost sharing or deductible or coinsurance or co insurance or benefit plan or capitation or cash payment or charge or fee or direct payment or direct contribution or pocket or expense or copay or co payment or co-pay or capitation or tier or tiered system or multitier or onetier or twotier or threetier or single pay))
3. SU prescription drugs OR SU pharmaceutical
4. TI ((drug or pharmaceutical or medicine or medicament or medication or prescription or prescribe)) OR AB ((drug or pharmaceutical or medicine or medicament or medication or prescription or prescribe))
5. SU health status OR SU health utilization OR SU drug utilization OR SU quality of life OR SU socieconomic status OR SU treatment outcomes OR SU health outcomes
6. AB ((health utilization or drug utilization or quality of life or socieconomic or ses or treatment outcome or disease outcome or health outcome or survival or remission or effectiveness or patient outcome)) OR TI ((health utilization or drug utilization or quality of life or socieconomic or ses or treatment outcome or disease outcome or health outcome or survival or remission or effectiveness or patient outcome))
7. (TX ((Canada or Canadian or Canadians or "British Columbia" or Alberta or Saskatchewan or Ontario or Quebec or "Nova Scotia" or "New Brunswick" or "Newfoundland and Labrador" or Newfoundland or "Northwest Territories" or Yukon or Nunavut or Vancouver or Calgary or Regina or Ottawa or Toronto or Montreal or “Quebec City” or Fredericton or Halifax or Charlottetown or “St. John’s” or Iqaluit or Yellowknife or Whitehorse)))
8. (1 OR 2) AND (3 OR 4) AND (5 OR 6) AND 7

Embase

1. exp national health insurance/
2. *health insurance/
3. *fee/
4. exp health care policy/
5. exp health program/
6. exp health care planning/
7. exp health maintenance organization/

8. *cost/

9. (cost? adj2 (share$ or sharing)).mp.

10. (deductible? or coinsurance or co insurance).mp.

11. (benefit plan? or capitation?).mp.

12. (cash adj1 pay$).tw.

13. ((charg$ or fee? or direct pay$ or direct contribut$) adj3 (patient? or prescrib$ or prescrip$ or pharmaceutic$ or pharmacy or pharmacies or dispens$)).ti. (1117)

14. ((pocket? adj3 pay$) or (copay$ or co pay$ or co-pay$)).mp.

15. ((limit$ or cap$ or restrict$ or reduc$ or regulat$) adj3 (prescrib$ or prescrip$ or reimburs$)).ti.

16. (tier or tiered system? or multitier$ or onetier$ or twotier$ or threetier$ or single pay$).mp.

17. 1 or 2 or 3 or 4 or 5 or 6 or 7 or 8 or 9 or 10 or 11 or 12 or 13 or 14 or 15 or 16

18. *drug prescription/

19. exp prescription drug/

20. (drug$ or pharmaceutic$ or medicines$ or medicament*? or medicat$ or prescrib$ or prescrip$).ti.

21. 18 or 19 or 20

22. *health status/

23. *"quality of life"/

24. *treatment outcome/

25. drug utilization/

26. *health service utilization/

27. ("socioeconomic factor$" or ses or "health outcome$" or qol or "quality of life" or "disease status" or disease$ or utili?ationS or "health service$").mp.

28. 22 or 23 or 24 or 25 or 26 or 27

29. exp Canada/

30. ("British Columbia" or Alberta or Saskatchewan or Manitoba or Ontario or Qubc or "New Brunswick" or "Nova Scotia" or "Newfoundland and Labrador" or Newfoundland or "Prince Edward Island" or "Northwest Territor*" or Nunavut or Yukon or Ottawa or Toronto or Vancouver).mp.

31. 29 or 30

32. 17 and 21 and 28 and 31

Medline

1. exp national health insurance/

2. *Insurance ,Health/

3. *Fees/ and Charges/

4. exp Health Policy/

5. exp Health Planning/

6. exp Health Maintenance Organizations/

7. *costs/ and cost analysis/

8. exp Medically Uninsured/

9. exp Insurance, Pharmaceutical Services/

10. (cost? adj2 (share$ or sharing)).mp.

11. (deductible? or coinsurance or co insurance).mp.

12. (benefit plan? or capitation?).mp.

13. (cash adj1 pay$).tw.

14. ((charg$ or fee? or direct pay$ or direct contribut$) adj3 (patient? or prescrib$ or prescrip$ or pharmaceutic$ or pharmacy or pharmacies or dispens$ or regulat$ or requirement? or restrict$ or monitor$ or control$ or legislation? or law? or act? or policy or policies or reform$ or system? or plan$ or program$ or strateg$ or state$ or government? or medicare or health maintenance organization? or hmo? or insurance)).tw.

15. ((pocket? adj3 pay$) or (copay$ or co pay$ or co-pay$)).mp.

16. ((limit$ or cap$ or restrict$ or reduc$ or regulat$) adj3 (prescrib$ or prescrip$ or reimburs$)).mp.

17. (tier or tiered system? or multitier$ or onetier$ or twotier$ or threetier$ or single pay$).mp.

18. 1 or 2 or 3 or 4 or 5 or 6 or 7 or 8 or 9 or 10 or 11 or 12 or 13 or 14 or 15 or 16 or 17

19. Drug Prescriptions/

20. exp Prescription Drugs/

21. (drug$ or pharmaceutic$ or medicines$ or medicament*? or medicat$ or prescrib$ or prescrip$).mp.

22. 19 or 20 or 21

23. exp Health Status/

24. Drug Utilization/

25. Health Services/ut [Utilization]

26. exp "quality of life"/

27. exp Socioeconomic Factors/

28. exp Treatment Outcome/

29. ("socioeconomic factor$" or ses or "health outcome$" or qol or "quality of life" or "disease status" or disease$ or utili?ationS or "health service$").mp.

30. 23 or 24 or 25 or 26 or 27 or 28 or 29

31. exp Canada/

32. ("British Columbia" or Alberta or Saskatchewan or Manitoba or Ontario or Qu$bec or "New Brunswick" or "Nova Scotia" or "Newfoundland and Labrador" or Newfoundland or "Prince Edward Island" or "Northwest Territor*" or Nunavut or Yukon or Ottawa or Toronto or Vancouver or Montr$al).mp.

33. 32 or 33

34. 18 and 22 and 30 and 33

Scopus

1. ((TITLE-ABS ("health insurance" OR cost OR fee OR "health policy" OR uninsured OR cost?sharing OR deductible OR co?insurance OR "benefit plan?" OR "benefit-plan" OR capitation OR payment OR charge OR copay OR copayer OR co-pay* OR capitation OR payer)) OR (KEY ("health insurance" or "health policy" or uninsured or fee)))

2. (TITLE (prescription OR drug OR pharmaceutical OR medicine OR medication OR medicament OR prescribe))

3. (ABS ("health status" OR utilization OR "quality of life" OR socieconomic or SES or survival OR remission OR "patient outcome*") OR (KEY ("health service utilization" or "drug utilization" or "quality of life" or "health status")))

4. 1 AND 2 AND 3

Appendix B. Quality Assessment / Risk of Bias tools

1. **Cochrane EPOC criteria**

- Randomized Control Trials (RCT) and controlled before and after studies (CBA): Random sequence generation; 2. Allocation concealment; 3. Baseline outcomes similarity; 4. Baseline characteristics similarity; 5. Incomplete outcome data assessment. 6. Blinding of outcome assessment; 7. Protection against contamination; 8. Selective outcome reporting; 9. Other risk of bias.
- Interrupted Time Series ITS (ITS)/Repeated Measures (RM): 1. Intervention independent of other changes; 2. Shape of the intervention prespecified; 3. Intervention unlikely to affect data collection (protection against detection bias); 4. Blinding of outcome assessment; 5. Incomplete outcome data assessment; 6. Avoidance of selective outcome reporting; 7. Other risk of bias.

https://epoc.cochrane.org/resources/epoc-resources-review-authors
https://epoc.cochrane.org/sites/epoc.cochrane.org/files/public/uploads/Resources-for-authors2017/suggested_risk_of_bias_criteria_for_epoc_reviews.pdf

2. **Newcastle-Ottawa Scale for Cohort studies**

- 1. Representativeness of the exposed cohort; 2. Selection of the non-exposed cohort; 3. Ascertainment of exposure; 4. Demonstration that outcome of interest was not present at start of study; 5. Comparability of cohorts on the basis of the design or analysis controlled for confounders; 6. Assessment of outcome; 7. Was follow-up long enough for outcomes to occur; (duration of follow-up) 8. Adequacy of follow-up of cohorts.

http://www.ohri.ca/programs/clinical_epidemiology/oxford.asp
http://www.ohri.ca/programs/clinical_epidemiology/nosgen.pdf

3. **Delphi list**

- 1. Was a method of randomization performed; 2. Was the treatment allocation concealed; 3. Were the groups similar at baseline regarding the most important prognostic indicators; 4. Were the eligibility criteria specified; 5. Was the outcome assessor blinded; 6. Was the care provider blinded; 7. Was the patient blinded; 8. Were point estimates (confidence intervals and/or odds ratios) and measures of variability presented for the primary outcome measures; 9. Did the analysis include an intention-to-treat analysis?

https://www.jclinepi.com/article/S0895-4356(98)00131-0/fulltext

4. **The Effective Public Health Practice Project (EPHPP) Rating Scale**

- 1. Selection bias; 2. Study design; 3. Confounders; 4. Blinding; 5. Data collection methods; 6. Withdrawals and dropouts; 7. Intervention integrity; 8. Analysis.

https://merst.ca/wp-content/uploads/2018/02/quality-assessment-tool_2010.pdf

5. **Cochrane risk of bias tool**

- 1. Random sequence generation; 2. Allocation concealment; 3. Blinding of participants and personnel; 4. Blinding of outcome assessment; 5. Incomplete outcome data; 6. Selective reporting; 7. Other sources of bias;

https://methods.cochrane.org/bias/resources/rob-2-revised-cochrane-risk-bias-tool-randomized-trials

6. **Downs and Black, 1998**

- The modified checklist based on Downs and Black (1998) consisted of 16 items with a maximum score of 14 points awarded for: study design; appropriate reporting of study objectives, methods and results; external validity and internal validity. The original checklist includes 27 items with a maximum score of 32 points. Items not relevant to the objectives of the review, including those specific to experimental studies, were removed.
  Downs SH, Black N. The feasibility of creating a checklist for the assessment of the methodological quality both of randomised and non-randomised studies of health care interventions. J Epidemiol Community Health 1998; 52(6): 377-84.

https://jech.bmj.com/content/52/6/377.abstract

7. **National Institute of Health (NIH) Quality Assessment Tool for Observational Cohort and Cross-Sectional Studies:**

- 1. Was the research question or objective in this paper clearly stated; 2. Was the study population clearly specified and defined; 3. Was the participation rate of eligible persons at least 50%; 4. Were all the subjects selected or recruited from the same or similar populations (including the same time period)? Were inclusion and exclusion criteria for being in the study prespecified and applied uniformly to all participants; 5. Was a sample size justification, power description, or variance and effect estimates provided; 6. For the analyses in this paper, were the exposure(s) of interest measured prior to the outcome(s) being measured; 7. Was the timeframe sufficient so that one could reasonably expect to see an association between exposure and outcome if it existed; 8. For exposures that can vary in amount or level, did the study examine different levels of the exposure as related to the outcome (e.g., categories of exposure, or exposure measured as continuous variable); 9. Were the exposure measures (independent variables) clearly defined, valid, reliable, and implemented consistently across all study participants; 10. Was the exposure(s) assessed more than once over time; 11. Were the outcome measures (dependent variables) clearly defined, valid, reliable, and implemented consistently across all study participants; 12. Were the outcome assessors blinded to the exposure status of participants; 13. Was loss to follow-up after baseline 20% or less; 14. Were key potential confounding variables measured and adjusted statistically for their impact on the relationship between exposure(s) and outcome(s).

https://www.nhlbi.nih.gov/health-topics/study-quality-assessment-tools

8. **Joanna Briggs Institute's critical appraisal checklist for RCTs**

- 1. Was true randomization used for assignment of participants to treatment groups; 2. Was allocation to treatment groups concealed; 3. Were treatment groups similar at the baseline; 4. Were participants blind to treatment assignment; 5. Were those delivering treatment blind to treatment assignment; 6. Were outcomes assessors blind to treatment assignment; 7. Were treatment groups treated identically other than the intervention of interest; 8. Was follow up complete and if not, were differences between groups in terms of their follow up adequately described and analyzed; 9. Were participants analyzed in the groups to which they were randomized; 10. Were outcomes measured in the same way for treatment groups; 11. Were outcomes measured in a reliable way; 12. Was appropriate statistical analysis used; 13. Was the trial design appropriate, and any deviations from the standard RCT design (individual randomization, parallel groups) accounted for in the conduct and analysis of the trial.

https://joannabriggs.org/critical-appraisal-tools
https://joannabriggs.org/sites/default/files/2020-08/Checklist_for_RCTs.pdf

9. **The Strengthening the Reporting of Observational Studies in Epidemiology (STROBE) Statement**

- The checklist comprises 6 domains and 22 criteria: 1. title and abstract 2. background/rationale 3. objectives 4. study design 5. setting 6. participants 7. variables 8. data sources/measurement 9. bias 10. study size 11. quantitative variables 12. statistical methods 13. participants 14. description data 15. outcome data 16. main results 17. other analyses 18. key results 19. limitations 20. interpretation 21. generalizability 22. funding.

https://www.acpjournals.org/doi/full/10.7326/0003-4819-147-8-200710160-00010#t1-10

10. **Gardner, Machin, Campbell, 1986**

- The adapted checklist comprised 2 domains* and 9 items: 1. description of study objective 2. Study design 3. Source population 4. sample size 5. description of statistical procedures 6. statistical analysis 7. Presentation of statistical material 8. presentation of confidence intervals 9. Appropriate conclusion. In cases where more than three out of nine quality criteria were not met, the given study was considered as of poor quality. Gardner et al.'s checklist has 4 domains and 12 items. The authors incorrectly stated that the domain 'conduct of the study' was used; the only item in this domain was not used.
  Gardner MJ, Machin D, Campbell MJ. Use of check lists in assessing the statistical content of medical studies. Br Med J (Clin Res Ed) 1986; 292(6523): 810-2

https://www.bmj.com/content/bmj/292/6523/810.full.pdf

Appendix C. Characteristics of included studies

Adams, Soumerai, Ross-Degnan, 2001 [1]

Type of review/publication	- Narrative review<br>- Journal: Annual Review of Public Health
Research question, overall	The effect of drug coverage on drug utilization, health outcomes, and health care costs in the Medicare population.
Research question, specific to drug insurance/cost-sharing	Same
Was an 'a priori' design provided?	No.
Was there duplicate study selection and data extraction?	Unclear.
Was a comprehensive literature search performed?	No.<br>- databases: Medline<br>- languages: not reported<br>- year/month of last search: not reported (1980-2000)<br>- grey literature included: yes<br>- keywords/search strategy reported: yes
Search strategy, results	- Total number of studies included: 37<br>- Total number of studies, drugs/cost-sharing/insurance: 37<br>- Total number of Canadian studies: 0
Was a list of studies (included and excluded) provided?	No, only references of included studies are given; the number of studies excluded and the reasoning for their exclusion is provided, however, there is no way for the reader to trace the excluded studies.
Were the characteristics of the included studies provided?	No.
Was the scientific quality of the included studies assessed and documented?	No formal quality assessment conducted. Criteria were used to assess the validity of findings including study design, appropriateness of study population, data quality and availability, reliability of measures of association and adequacy of statistical analysis. The strengths and limitations of study designs were generally described,
Was the scientific quality of the included studies used appropriately in formulating conclusions?	To some extent the quality of evidence was generally discussed and taken to account.
Were the methods used to combine the findings of studies appropriate?	n/a
Was the likelihood of publication bias assessed?	n/a
Funding, conflicts of interest reported?	Funding:<br>- review: no<br>- included studies: no<br>Conflict of interest:<br>- review: no<br>- included studies: no

Study's conclusion (as stated by the authors)	Current literature provides considerable evidence that drug coverage is associated with greater use of all drugs and clinically essential medications;<br>Although evidence of the link between coverage, health outcomes, and care costs was sparse, the longitudinal analyses in elderly Medicaid populations found that limiting the number of reimbursable prescriptions per month had serious adverse health outcomes for sick and low-income beneficiaries. In some cases, the adverse events caused by the drug cap were irreversible (i.e. nursing home admissions).
Limitations/risk of bias	- no 'a priori' design;<br>- non-systematic search strategy;<br>- no/unclear duplicate study selection and data extraction;<br>- list of excluded studies not provided;<br>- unclear screening and data extraction process;<br>- study characteristics of studies not provided;<br>- no formal quality assessment of included studies.
Results - drug use (average effects)	n/a
Results - drug use (by sub-groups such as SES, chronically ill, elderly, ...)	In the US Medicare population (65+ years), drug coverage was associated with greater use of all drugs and clinically essential medications;<br>*Magnitude, seniors*: Reductions in drug use ranged between 21-46% depending on the drug class and condition of patients.<br>*Magnitude, seniors vs. non-seniors*: unclear
Results - health outcomes	n/a
Results - health outcomes (by sub-groups such as SES, chronically ill, elderly, ...)	Some evidence that cost-sharing and limits on the the number of reimbursable prescriptions led to serious adverse health outcomes for sick and low-income Medicare beneficiaries (nursing home admissions, use of clinic emergency mental health services by schizophrenic patients)<br>*Magnitude, seniors:* unclear<br>*Magnitude, seniors vs. non-seniors:* unclear
Results - healthcare services utilization (i.e., non-pharmaceutical services)	n/a
Results - healthcare services utilization (i.e., non-pharmaceutical services) (by sub-groups such as SES, chronically ill, elderly, ...)	The association between prescription drug insurance/cost-sharing and healthcare services utilization were not explicitly discussed. In the New Hampshire drug cap studies, an increase in nursing home admissions for chronically ill elderly persons was affected by the cap. Hospitalizations during the period of the cap also increased but the difference was not statistically significant. For patients with schizophrenia, use of emergency mental health services and partial hospitalization during the time of the cap increased, and then decreased to near pre-cap levels after the cap was repealed;<br>*Magnitude, elderly:* Elderly Medicaid enrolees in New Hampshire were almost twice as likely to be admitted to nursing homes during the period of the cap as those in New Jersey (RR 1.8; 95%CI 1.2, 2.6). In addition, there was a slight trend toward higher rates of hospitalization in the New Hampshire cohort during the period of the cap, but this difference was not statistically significant (RR 1.2; 95%CI 0.8, 1.6). For patients with schizophrenia, use of emergency mental health services and partial hospitalization during the time of the cap increased 57%.<br>*Magnitude, elderly vs. non-elderly:* unclear

Harten, Ballantyne, 2004 [2]

Type of review/publication	- Narrative review<br>- journal: Journal of Pharmaceutical Finance, Economics, and Policy
Research question, overall	Canadian evidence of the effects of cost-sharing mechanisms of provincial drug benefit programs on program expenditures, drug utilization and patient health.
Research question, specific to drug insurance/cost-sharing	Canadian evidence of the effects of cost-sharing mechanisms of provincial drug benefit programs on drug utilization and patient health.
Was an 'a priori' design provided?	No
Was there duplicate study selection and data extraction?	Unclear.
Was a comprehensive literature search performed?	No.<br>- databases: Medline;<br>- languages: not reported;<br>- year/month of last search: July 2002;<br>- grey literature included: yes;<br>- keywords/search strategy reported: yes.
Search strategy, results	- Total number of studies included: 7<br>- Total number of studies, drugs/cost-sharing/insurance: 7<br>- Total number of Canadian studies: 7<br>• BC: Anderson, Kerluke, et al., 1993;<br>• AB: Fassbender, Pickard, 2000;<br>• MB: Kozyrskyj, Mustard, Cheang, Simons, 2001;<br>• ON: Hux, Fielding, 1997;<br>• QC: Poirier, LeLorier, et al., 1998; Blais, Boucher, et al., 2001; Tamblyn, Laprise et al., 2001.
Was a list of studies (included and excluded) provided?	No; list of excluded studies not provided.
Were the characteristics of the included studies provided?	To some extent. Certain characteristics of included studies were provided narratively and in tables, such as the cost-sharing policy and outcome measured.
Was the scientific quality of the included studies assessed and documented?	No formal quality assessment conducted. Limitations of included studies generally discussed.
Was the scientific quality of the included studies used appropriately in formulating conclusions?	To some extent; the authors did refer to the quality of the included studies to inform the analyses.

Were the methods used to combine the findings of studies appropriate?	n/a
Was the likelihood of publication bias assessed?	n/a
Funding, conflicts of interest reported?	Funding:<br>- review: no<br>- included studies: no<br>Conflict of interest:<br>- review: no<br>- included studies: no
Study's conclusion (as stated by the authors)	Our review suggests that cost sharing decreases program expenditures by shifting costs to patients and decreasing patients' use of essential and discretionary medications.
Limitations/risk of bias	- no 'a priori' design;<br>- unclear duplicate study selection and data extraction;<br>- non-systematic search strategy;<br>- search limited to English;<br>- list of excluded studies not provided;<br>- no formal quality assessment of included studies.
Results - drug use (average effects)	Found either no change in utilization or a decrease in essential and discretionary medications following introduction of or increases in cost sharing.<br>*Magnitude*: unclear
Results - drug use (by sub-groups such as SES, chronically ill, elderly, ...)	n/a
Results - health outcomes	Only one included study examined health outcomes. It found that drug cost-sharing was associated with a decrease in essential drugs, which was associated with an increase in adverse events as measured by hospitalization, nursing home admissions and mortality (in seniors and welfare recipients);<br>*Magnitude:* unclear
Results - health outcomes (by sub-groups such as SES, chronically ill, elderly, ...)	n/a
Results - healthcare services utilization (i.e., non-pharmaceutical services)	n/a

Results - healthcare services utilization (i.e., non-pharmaceutical services) (by sub-groups such as SES, chronically ill, elderly, ...)	n/a

Lexchin, Grootendorst, 2004 [3]

Type of review/publication	- Narrative review<br>- Journal: International Journal of Health Services
Research question, overall	The effect of drug user fees on drug use and related outcomes in vulnerable populations (the poor and chronically ill)
Research question, specific to drug insurance/cost-sharing	Same.
Was an 'a priori' design provided?	No.
Was there duplicate study selection and data extraction?	Yes.
Was a comprehensive literature search performed?	Yes.<br>- databases: Medline, HealthSTAR and EconLit;<br>- languages: English, French;<br>- year/month of last search: August 2002;<br>- grey literature included: yes;<br>- keyword/search strategy reported: no.
Search strategy, results	- Total number of studies: 24<br>- Total number of studies, drugs/cost-sharing/insurance: 24<br>- Total number of Canadian studies: 5<br>• MB: Kozyrskyj, Mustard, Cheang, Simons, 2001;<br>• ON: Grootendorst, 1997; Grootendorst, Feeny, Furlong, 1997;<br>• QC: Blais, Castilloux et al., 1999; Tamblyn, Laprise et al., 2001.
Was a list of studies (included and excluded) provided?	Yes.
Were the characteristics of the included studies provided?	Yes; author, dates, study population (data source), outcomes, price variation, design, results.
Was the scientific quality of the included studies assessed and documented?	No.
Was the scientific quality of the included studies used appropriately in formulating conclusions?	n/a
Were the methods used to combine the findings of studies appropriate?	n/a
Was the likelihood of publication bias assessed?	n/a
Funding, conflicts of interest reported?	Funding:<br>- review: yes; National Health and Development Research Program (NHRDP); Rx&D Health Research Foundation, Canadian Institutes for Health Research;<br>- included studies: no;<br>Conflict of interest:<br>- review: no;<br>- included studies: no.

Study's conclusion (as stated by the authors)	Cost sharing through the use of copayments or deductibles decreased the use of prescription drugs by the poor and the chronically ill. Drug price elasticities among vulnerable groups—those with low income and/or chronic illnesses generally ranged from –0.34 to –0.50.
Limitations/risk of bias	- no 'a priori' design;<br>- no quality assessment of included studies.
Results - drug use (average effects)	n/a
Results - drug use (by sub-groups such as SES, chronically ill, elderly, ...)	Cost-sharing through the use of copayments or deductibles decreased the use of prescription drugs by the poor and the chronically ill.<br>*Magnitude, poor/chronically ill*: Drug price elasticities among vulnerable groups—those with low income and/or chronic illnesses generally ranged from –0.34 to –0.50. Some evidence that cost-sharing led to patients foregoing essential medications.<br>*Magnitude, poor/chronically ill*: *vs. non-poor/chronically ill*:: unclear
Results - health outcomes	n/a
Results - health outcomes (by sub-groups such as SES, chronically ill, elderly, ...)	Some evidence that drug cost-sharing led to increases in serious adverse events (defined as the first occurrence of acute care hospitalization, long-term care admission, or death; nursing home admission, use of emergency mental health services among those with schizophrenia)<br>*Magnitude, poor/chronically ill:* unclear<br>*Magnitude, poor/chronically ill vs. non-poor/chronically ill:* unclear
Results - healthcare services utilization (i.e., non-pharmaceutical services)	n/a
Results - healthcare services utilization (i.e., non-pharmaceutical services) (by sub-groups such as SES, chronically ill, elderly, ...)	Some evidence that prescription drug cost-sharing led to increases in use of emergency services (acute care hospitalization, emergency room admission, long-term care admission), and nursing home admissions;<br>*Magnitude, poor/chronically ill:* unclear<br>*Magnitude, poor/chronically ill vs. non-poor/chronically ill:* unclear

Rice, Matsuoka, 2004 [4]

Type of review/publication	- Narrative review<br>- Journal: Medical Care Research & Review
Research question, overall	What is the impact of cost-sharing for both medical services and prescription drugs on service use and health status of seniors?
Research question, specific to drug insurance/cost-sharing	Same.
Was an 'a priori' design provided?	No.
Was there duplicate study selection and data extraction?	Unclear.
Was a comprehensive literature search performed?	No.<br>- databases: PubMed, Ingenta, EconLit, Science-direct;<br>- languages: not reported;<br>- year/month of last search: not reported;<br>- grey literature included: no;<br>- keyword/search strategy reported: yes.
Search strategy, results	- Total number of studies: 22<br>- Total number of studies, drugs/cost-sharing/insurance: 16<br>- Total number of Canadian studies: 4<br>• BC: Schneeweiss, Walker et al., 2002<br>• QC: Tamblyn, Laprise et al., 2001; Pilote, Beck, et al., 2002; Blais, Boucher et al., 2001.
Was a list of studies (included and excluded) provided?	No; only references of included studies are given; the number of studies excluded and the reasoning for their exclusion is provided, however, there is no way for the reader to trace the excluded studies.
Were the characteristics of the included studies provided?	Yes; sample/data source, cost-arrangement, impact on service utilization, health outcome and study limitations.
Was the scientific quality of the included studies assessed and documented?	To some extent; no formal quality assessment approach/tool used but the quality of individual studies was generally discussed throughout and study limitations are identified for each included study.
Was the scientific quality of the included studies used appropriately in formulating conclusions?	Yes. The authors discussed individual study limitations throughout their results and in the limitations section of the review.
Were the methods used to combine the findings of studies appropriate?	n/a
Was the likelihood of publication bias assessed?	n/a
Funding, conflicts of interest reported?	Funding:<br>- review: yes; Henry J. Kaiser Family Foundation;<br>- included studies: no;<br>Conflict of interest:<br>- review: yes;<br>- included studies: no.

Study's conclusion (as stated by the authors)	Increased cost-sharing reduced either or both the utilization and health status of seniors (with the exception of life-threatening emergencies)
Limitations/risk of bias	- no 'a priori' design;<br>- grey literature not searched;<br>- unclear screening and data extraction process (inclusion and exclusion criteria not stated)<br>- list of excluded studies not provided;<br>- no formal quality assessment of included studies;<br>- narrow inclusion criteria (only studies from US and Canada were included).
Results - drug use (average effects)	n/a
Results - drug use (by sub-groups such as SES, chronically ill, elderly, ...)	Among seniors, cost-sharing (not necessarily for drugs) was found to reduce the appropriate use of prescription drugs (medications that are thought to improve health status);<br>*Magnitude, seniors*: unclear<br>*Magnitude, seniors vs. non-seniors*: unclear
Results - health outcomes	n/a
Results - health outcomes (by sub-groups such as SES, chronically ill, elderly, ...)	Among seniors, cost-sharing (not necessarily drug) can result in lower health status (either higher mortality or various measures of morbidity), with the following two notable exceptions: 1) when generous provisions are in place to protect vulnerable populations from incurring undue financial risk as a result of cost sharing, 2) the case of patients experiencing serious medical events because they realize the necessity of receiving recommended medical care irrespective of cost-sharing requirements.<br>*Magnitude, seniors:* unclear<br>*Magnitude, seniors vs. non-seniors:* unclear
Results - healthcare services utilization (i.e., non-pharmaceutical services)	n/a
Results - healthcare services utilization (i.e., non-pharmaceutical services) (by sub-groups such as SES, chronically ill, elderly, ...)	Results were contradictory and not conclusive for hospitalization and long-term care admission rates in response to cost-sharing or prescription drug payment limits. However, this review also generally found that having some form of supplemental insurance was associated with more appropriate health care use, particularly when such supplemental insurance provided coverage for prescription medication;<br>*Magnitude, elderly:* unclear<br>*Magnitude, elderly vs. non-elderly:* unclear

Gibson, Ozminkowsky, Goetzel, 2005 [5]

Type of review/publication	- Narrative review<br>- Journal: The American Journal of Managed Care
Research question, overall	Do patients respond to increased cost-sharing by substituting less expensive alternatives for medications with higher levels of copayments or coinsurance?
Research question, specific to drug insurance/cost-sharing	Same.
Was an 'a priori' design provided?	No.
Was there duplicate study selection and data extraction?	Unclear
Was a comprehensive literature search performed?	No.<br>- databases: Medline;<br>- languages: English only;<br>- year/month of last search: April 2005 (1974-2005);<br>- grey literature included: no<br>- keyword/search strategy reported: yes.
Search strategy, results	- Total number of studies included: 30<br>- Total number of studies, drugs/cost-sharing/insurance: 30<br>- Total number of Canadian studies: 4<br>• QC: Blais, Boucher et al. 2001; Blais, Couture et al.2003; Tamblyn, Laprise et al. 2001; Pilote, Beck et al. 2002
Was a list of studies (included and excluded) provided?	No; only the references of included studies are provided; the number of studies excluded and the reasoning for their exclusion is provided, however, there is no way for the reader to trace the excluded studies.
Were the characteristics of the included studies provided?	No.
Was the scientific quality of the included studies assessed and documented?	No.
Was the scientific quality of the included studies used appropriately in formulating conclusions?	No
Were the methods used to combine the findings of studies appropriate?	n/a
Was the likelihood of publication bias assessed?	n/a
Funding, conflicts of interest reported?	Funding:<br>- review: yes; GlaxoSmithKline<br>- included studies: no<br>Conflict of interest:<br>- review: yes<br>- included studies: no

Study's conclusion (as stated by the authors)	The result of increased cost sharing is reduced consumption of prescription drugs which may also have unintended effects of disrupting the process and outcomes of therapy.
Limitations/risk of bias	- no 'a priori' design;<br>- search limited to English;<br>- narrow inclusion criteria (only studies from US and Canada were included);<br>- narrow inclusion criteria (only included studies that used claims-based data sources);<br>- unclear inclusion/exclusion criteria;<br>- list of excluded studies not provided;<br>- study characteristics not clearly presented and/or synthesized;<br>- no formal quality assessment of included studies.
Results - drug use (average effects)	– Demand for prescription drugs<br>Higher levels of drug cost-sharing resulted in reductions in prescription drug use.<br>*Magnitude:* Most estimates of own-price elasticity suggested that a 10% increase in price decreased use by 1 to 4%;<br>– Medication adherence:<br>Patients facing cost-sharing were less likely to adhere to prescribed medications;<br>*Magnitude:* unclear<br>– Non-preferred vs. preferred brand-name drugs:<br>All studies reviewed showed that increasing drug cost-sharing for non-preferred brand-name drugs decreased use of non-preferred brand-name drugs and increased use of preferred brand-name drugs;<br>*Magnitude:* unclear<br>– Generic substitution:<br>Little evidence of generic substitution in plans introducing or increasing a generic vs brand cost-sharing differential;<br>*Magnitude:* unclear<br>– Substitution of over-the-counter drugs for prescription drugs:<br>Limited and inconclusive findings;<br>*Magnitude:* unclear<br>– Essential medications:<br>Higher levels of prescription drug cost-sharing were associated with a reduction in the consumption of essential medications;<br>*Magnitude:* unclear
Results - drug use (by sub-groups such as SES, chronically ill, elderly, ...)	n/a
Results - health outcomes	No studies were identified that measured the effects of prescription drug cost-sharing on direct measures of health status, such as self-reported health status and empirical measures of clinical health status (eg, laboratory readings). One study found that higher levels of cost-sharing had no effect on mortality rates while another reported an indirect decline in claims-based score of health status because of a copayment increase from $1 to $3 but not when there there was a copayment increase from 50% with a $25 maximum to 70% with a $30 maximum;<br>*Magnitude:* unclear
Results - health outcomes (by sub-groups such as SES, chronically ill, elderly, ...)	n/a

Results - healthcare services utilization (i.e., non-pharmaceutical services)	In most studies, higher levels of prescription drug cost-sharing were not associated with changes in the utilization of low-intensity outpatient medical services, such as physician office visits, outpatient visits, and home health visits. However, these studies assessed small changes in prescription drug cost sharing. Two studies reported an increase in high-intensity health services (such as inpatient visits, emergency department visits, readmissions among older patients hospitalized with complications after acute myocardial infarction) as cost-sharing rose in some diagnostic groups (congestive heart failure or coronary artery disease) while not in others (diabetes mellitus). Four studies reported no association between higher levels of cost-sharing and high-intensity services.<br>*Magnitude:* unclear
Results - healthcare services utilization (i.e., non-pharmaceutical services) (by sub-groups such as SES, chronically ill, elderly, ...)	n/a

Maio, Pizzi, Roumm, 2005 [6]

Type of review/publication	- Narrative review<br>- Journal: The Milbank Quarterly
Research question, overall	Among seniors, the effects of cost-sharing mechanisms such as copayments, coinsurance, deductibles, and benefit caps; and administrative mechanisms such as prior authorization and formularies on prescription drug utilization and/or expenditures, other health services such as hospital admissions and office visits, underuse of effective medications, clinical outcomes, adverse events; and on the subject's behavior, such as voluntary disenrollment.
Research question, specific to drug insurance/cost-sharing	Among seniors, the effects of cost-sharing mechanisms such as copayments, coinsurance, deductibles, and benefit caps; on prescription drug utilization and/or expenditures, other health services such as hospital admissions and office visits, underuse of effective medications, clinical outcomes, adverse events; and on the subject's behavior, such as voluntary disenrollment.
Was an 'a priori' design provided?	No.
Was there duplicate study selection and data extraction?	Unclear.
Was a comprehensive literature search performed?	To some extent.<br>- databases: Medline, CINAHL;<br>- languages: English only;<br>- year/month of last search: May 2003;<br>- grey literature included: no<br>- keyword/search strategy reported: yes.
Search strategy, results	- Total number of studies included: 16<br>- Total number of studies, drugs/cost-sharing/insurance: 7<br>- Total number of Canadian studies: 3<br>• QC: Blais, Boucher, et al. 2001, Tamblyn, Laprise, Hanley, et al. 2001, Pilote, Beck et al. 2002.
Was a list of studies (included and excluded) provided?	No. A list of included studies provided but not a list of excluded studies.
Were the characteristics of the included studies provided?	Yes; authors; setting; study design; post-test design, study sample; outcomes measures; results.
Was the scientific quality of the included studies assessed and documented?	To some extent. The quality of quasi-experimental studies was generally discussed but not of randomized studies.
Was the scientific quality of the included studies used appropriately in formulating conclusions?	To some extent; the quality of individual studies was generally discussed and taken into account.
Were the methods used to combine the findings of studies appropriate?	n/a
Was the likelihood of publication bias assessed?	n/a

Funding, conflicts of interest reported?	Funding:<br>- review: yes; Project Patient Care<br>- included studies: no<br>Conflict of interest:<br>- review: yes<br>- included studies: no
Study's conclusion (as stated by the authors)	Overall, our review showed that the impacts of pharmacy utilization management measures such as cost-sharing and administrative mechanisms on seniors' health have not been adequately evaluated. Seniors with chronic conditions requiring extensive pharmaceutical management may be prevented from obtaining drug benefits during times of need.
Limitations/risk of bias	- no 'a priori' design;<br>- non-systematic search strategy;<br>- search limited to English;<br>- list of excluded studies not provided;<br>- grey literature not searched;<br>- no formal quality assessment of included studies.<br>- no/unclear duplicate study selection and data extraction.
Results - drug use (average effects)	n/a
Results - drug use (by sub-groups such as SES, chronically ill, elderly, ...)	There is mixed evidence that prescription cost-sharing mechanisms (copayment, coinsurance, and deductible) reduced seniors' drug use. There is some evidence that for low-income populations, even small copayments may have led them to reduce their use of effective medications;<br>*Magnitude, seniors*: unclear<br>*Magnitude, seniors vs. non-seniors*: unclear
Results - health outcomes	n/a
Results - health outcomes (by sub-groups such as SES, chronically ill, elderly, ...)	There was mixed evidence that prescription drug cost-sharing mechanisms (copayment, coinsurance, and deductible) had negative effects on seniors' health.<br>*Magnitude, seniors:* unclear<br>*Magnitude, seniors vs. non-seniors:* unclear
Results - healthcare services utilization (i.e., non-pharmaceutical services)	n/a
Results - healthcare services utilization (i.e., non-pharmaceutical services) (by sub-groups such as SES, chronically ill, elderly, ...)	For seniors, prescription drug cost-sharing and the use of caps may have led to greater risk of hospitalization or admittance to nursing home facilities;<br>*Magnitude, elderly:* unclear<br>*Magnitude, elderly vs. non-elderly:* unclear

Briesacher, Gurwitz, Soumerai, 2007 [7]

Type of review/publication	- Narrative review<br>- Journal: J Gen Intern Med
Research question, overall	To identify patient-, medication-, and provider-level factors that influence the relationship between medication adherence and medication costs.
Research question, specific to drug insurance/cost-sharing	Same.
Was an 'a priori' design provided?	No.
Was there duplicate study selection and data extraction?	No; only one reviewer extracted the data from individual studies; 3 reviewers selected studies for inclusion.
Was a comprehensive literature search performed?	Yes.<br>- databases: MEDLINE, CINAHL, Sciences Citations Index Expanded, EconLit;<br>- languages: English only;<br>- year/month of last search: October 2006;<br>- grey literature included: no<br>- keyword/search strategy reported: yes.
Search strategy, results	- Total number of studies included: 20 articles (19 independent studies)<br>- Total number of studies, drugs/cost-sharing/insurance:: 17<br>- Total number of Canadian studies: 0
Was a list of studies (included and excluded) provided?	Yes; a list of included studies was provided. References of excluded studies were provided and reasons for exclusion were generally discussed.
Were the characteristics of the included studies provided?	No; partial information was provided as the studies were grouped into categories and summarized for each categories.
Was the scientific quality of the included studies assessed and documented?	No formal quality assessment of included studies. Limitations of included studies were generally discussed.
Was the scientific quality of the included studies used appropriately in formulating conclusions?	To some extent.
Were the methods used to combine the findings of studies appropriate?	n/a
Was the likelihood of publication bias assessed?	n/a
Funding, conflicts of interest reported?	Funding:<br>- review: yes; National Institute on Aging, US Agency for Healthcare Research and Quality;<br>- included studies: no<br>Conflict of interest:<br>- review: yes; Novartis Pharmaceuticals Corporation<br>- included studies: no
Study's conclusion (as stated by the authors)	Strong evidence that among medicare beneficiaries, drug coverage decreased the risk of cost-related medication nonadherence;<br>Strong evidence that among medicare beneficiaries and adults 50+ higher cost-sharing increased the risk of cost-related medication nonadherence.

Limitations/risk of bias	- no 'a priori' design;<br>- no/unclear duplicate study selection and data extraction;<br>- search limited to English;<br>- grey literature not searched;<br>- study characteristics of included studies not provided;<br>- no formal quality assessment of included studies.
Results - drug use (average effects)	Not having prescription drug coverage was a significant and robust risk factor for cost-related non-adherence in all reviewed studies;<br>*Magnitude***:** unclear; duration of coverage and type of coverage affected the magnitude of association.
Results - drug use (by sub-groups such as SES, chronically ill, elderly, ...)	Strong evidence that among medicare beneficiaries, drug coverage decreased the risk of cost-related medication non-adherence; strong evidence that among medicare beneficiaries and adults 50+, higher cost-sharing increased the risk of cost-related medication non-adherence.<br>*Magnitude, older adults, seniors*: unclear<br>*Magnitude, older adults, seniors*: *vs. non-older adults, non-seniors*: unclear
Results - health outcomes	n/a
Results - health outcomes (by sub-groups such as SES, chronically ill, elderly, ...)	n/a
Results - healthcare services utilization (i.e., non-pharmaceutical services)	n/a
Results - healthcare services utilization (i.e., non-pharmaceutical services) (by sub-groups such as SES, chronically ill, elderly, ...)	n/a

Gemmil, Costa-Font, McGuire, 2007 [8]

Type of review/publication	- Meta-regression<br>- Journal: Health Economics
Research question, overall	To determine an estimate for drug-price elasticity using meta-regression analysis
Research question, specific to drug insurance/cost-sharing	Same.
Was an 'a priori' design provided?	No.
Was there duplicate study selection and data extraction?	Can't answer - there was no explicit information provided on the selection and data extraction process.
Was a comprehensive literature search performed?	No<br>- databases: PubMed, EconLit, Blackwell's Synergy, and Ingenta;<br>- languages: not mentioned;<br>- year/month of last search: not mentioned;<br>- grey literature included: no;<br>- keyword/search strategy reported: yes.
Search strategy, results	- Total number of studies included: 31<br>- Total number of studies, drugs/cost-sharing/insurance: 31<br>- - Total number of Canadian studies: 6<br>• ON: Greenlick, Darsky, 1968; Grootendorst, Feeny, Furlong, 1997;<br>• QC: Blais, Castilloux et al., 1999; Blais, Boucher et al., 2001; Contoyannis, Hurley et al., 2005;<br>• CA: Grootendorst, Levine, 2001.
Was a list of studies (included and excluded) provided?	No, only references of included studies were given; the number of studies excluded and the reasoning for their exclusion was provided, however, there is no way for the reader to trace the excluded studies.
Were the characteristics of the included studies provided?	Yes; population, policy variable.
Was the scientific quality of the included studies assessed and documented?	No. Authors discussed publication bias and examined the characteristics of published studies as quality controls (type of journal, aggregate or individual-level data, standard errors,…) and generally commented on quality, but no formal assessment of individual studies was conducted.
Was the scientific quality of the included studies used appropriately in formulating conclusions?	Yes. The authors discuss the general limitations of the included studies.
Were the methods used to combine the findings of studies appropriate?	n/a
Was the likelihood of publication bias assessed?	Yes; funnel plot.

Funding, conflicts of interest reported?	Funding:<br>- review: yes; Merck Company Foundation Program on Pharmaceutical Policy Issues and Generalitat de Catalunya;<br>- included studies: no;<br>Conflict of interest:<br>- review: yes;<br>- included studies: not mentioned
Study's conclusion (as stated by the authors)	The predicted drug elasticity estimates of demand for prescription drugs was -0.209, indicating that across high-income countries, consumers were not particularly responsive to changes in out-of-pocket prices. Consumers in tax-based health insurance systems were found to be less sensitive to out-of-pocket prices than consumers in other types of systems. Most of the estimates reported were at lower end of demand curve and as such, values may be higher for higher levels of cost-sharing.
Limitations/risk of bias	- no 'a priori' design;<br>- no/unclear duplicate study selection and data extraction;<br>- search strategy poorly described;<br>- search limited to English;<br>- grey literature not searched;<br>- study characteristics of included studies not provided;<br>- poorly justified or unclear exclusion criteria;<br>- no formal quality assessment of included studies.
Results - drug use (average effects)	Drug elasticity estimates were significantly different from zero, and the estimated corrected elasticity = -0.21 when the results were made robust to heteroskedasticity and clustering of observations. Elasticity values were higher when the study was published in an economic journal, when the study employed a greater number of observations, and when the study used aggregate data. Elasticity estimates were lower when the institutional setting was a tax-based health insurance system.<br>Higher cost-sharing was negatively associated with the demand for prescription drugs;<br>*Magnitude*: The demand for prescription drugs was relatively inelastic. The estimated corrected own-price elasticity = -0.21 (mean standard error of 0.026); a 10% increase in cost-sharing was associated with a 2% decrease in pharmaceutical spending.
Results - drug use (by sub-groups such as SES, chronically ill, elderly, ...)	n/a
Results - health outcomes	n/a
Results - health outcomes (by sub-groups such as SES, chronically ill, elderly, ...)	n/a
Results - healthcare services utilization (i.e., non-pharmaceutical services)	n/a
Results - healthcare services utilization (i.e., non-pharmaceutical services) (by sub-groups such as SES, chronically ill, elderly, ...)	n/a

Goldman, Joyce, Zheng, 2007 [9]

Type of review/publication	- Narrative review<br>- Journal: JAMA
Research question, overall	Associations among cost-sharing features of prescription drug benefits and use of prescription drugs, use of non-pharmaceutical services, and health outcomes.
Research question, specific to drug insurance/cost-sharing	Same.
Was an 'a priori' design provided?	No.
Was there duplicate study selection and data extraction?	Unclear.
Was a comprehensive literature search performed?	No.<br>- databases: PubMeD;<br>- languages: English;<br>- year/month of last search: not reported;<br>- grey literature included: no<br>- keyword/search strategy reported: no.
Search strategy, results	- Total number of studies examined for analysis : 132<br>- Total number of studies, drugs/cost-sharing/insurance: 132<br>- Total number of Canadian studies: 26<br>• BC: Narine, Senathirajah, Smith, 1999; Narine, Senathirajah, Smith,, 2001; Grootendorst, Dolovich et al., 2001; Marshall, Grootendorst et al., 2002; Hazlet, Blough, 2002; Schneeweiss, Soumerai et al., 2002; Schneeweiss, Walker et al., 2002; Schneeweiss, Soumerai et al., 2003; Schneeweiss, Dormuth et al., 2004; Schneeweiss, Maclure et al., 2006; Li, Guh et al., 2007; Gootendorst, Marshall et al., 2005; Anis, Guh et al., 2005; Dormuth, Glynn et al., 2006;<br>• AB: Ackman, Graham et al., 2006;<br>• MB: Kozyrskyj, Mustard, Cheang, Simons, 2001; Kozyrskyj, Mustard, Simons, 2001;<br>• ON: Grootendorst, O'Brien, Anderson, 1997;<br>• QC: Tamblyn, Laprise et al., 2001; Blais, Boucher et al., 2001; Pilote, Beck et al., 2002; Blais, Couture et al., 2003; Contoyannis, Hurley et al., 2005;<br>• NS: Campbell, Cooke et al., 2003; Mabasa, Ma., 2006.
Was a list of studies (included and excluded) provided?	No; only references of included studies are given; the number of studies excluded and the reasoning for their exclusion is provided, however, there is no way for the reader to trace the excluded studies.
Were the characteristics of the included studies provided?	Yes; source, study sample, study design, drug benefit variation, outcomes; key findings.
Was the scientific quality of the included studies assessed and documented?	No formal quality assessment of included studies. Limitations of included studies were generally discussed.
Was the scientific quality of the included studies used appropriately in formulating conclusions?	To some extent; the quality of individual studies was generally discussed and taken into account.
Were the methods used to combine the findings of studies appropriate?	n/a

Was the likelihood of publication bias assessed?	n/a
Funding, conflicts of interest reported?	Funding:<br>- review: yes; National Institute on Aging through its support of the RAND Roybal Center for Health Policy Simulation and by the Peter Bing Center for Health Economics at RAND<br>- included studies: no<br>Conflict of interest:<br>- review: no<br>- included studies: no
Study's conclusion (as stated by the authors)	Increased cost-sharing was associated with lower rates of drug treatment, worse adherence among existing users and more frequent discontinuation of therapy. For each 10% increase in cost-sharing, prescription drug spending decreased by 2-6% (depending on class of drug and condition of patient). For certain chronic conditions, higher cost-sharing was associated with increased use of medical services (those with congestive heart failure, schizophrenia, diabetes, lipid disorders). There was little evidence that individuals of lower-income were more sensitive to increased cost sharing.
Limitations/risk of bias	- no 'a priori' design;<br>- non-systematic search strategy;<br>- no formal quality assessment of included studies.<br>- grey literature not searched;<br>- no/unclear duplicate study selection and data extraction;<br>- list of excluded studies not provided;
Results - drug use (average effects)	– Demand for prescription drugs<br>Higher cost-sharing was negatively associated with the demand for prescription drugs;<br>*Magnitude*: The demand for prescription drugs was relatively inelastic. Own-price elasticities ranged from -0.2 to -0.6; cost-sharing increases of 10% (through either higher co-payments or coinsurance) were associated with a 2% to 6% decline in prescription drug use. The magnitude of association depended on class of drug and condition of patients.<br>– Essential and nonessential drug use<br>Mixed effects of the impact of copayments on essential drug use;<br>*Magnitude***:** unclear
Results - drug use (by sub-groups such as SES, chronically ill, elderly, ...)	– Low-income<br>Although studies suggest that low-income beneficiaries reduced drug use with higher copayments, there was little evidence that individuals of lower-income were more sensitive to increased cost-sharing than the general population;<br>*Magnitude, low-income*: same as the general population;<br>*Magnitude, low-income vs. non-low-income*: same as the general population.<br>– Chronically ill<br>The evidence suggests that even chronically ill patients were responsive to cost-sharing;<br>*Magnitude, chronically ill*: unclear<br>*Magnitude, chronically ill vs. non-chronically ill*: unclear
Results - health outcomes	The direct evidence on the link between prescription drug cost-sharing and health was limited. Most studies found that when the population was not limited to those with certain chronic illnesses, the outcomes associated with prescription drug cost-sharing were mostly benign. Studies that looked at cost-sharing effects more broadly were ambiguous in their findings;<br>*Magnitude:* unclear
Results - health outcomes (by sub-groups such as SES, chronically ill, elderly, ...)	Some studies found that higher cost-sharing was associated with adverse outcomes especially among vulnerable populations such as the elderly and poor.<br>*Magnitude, poor/chronically ill:* unclear<br>*Magnitude, poor/chronically ill vs. non-poor/chronically ill:* unclear

Results - healthcare services utilization (i.e., non-pharmaceutical services)	Increased drug copayments were not associated with more outpatient visits, hospitalizations, or emergency department visits among a broader population (not restricted to the elderly or those with chronic conditions);<br>*Magnitude:* n/a
Results - healthcare services utilization (i.e., non-pharmaceutical services) (by sub-groups such as SES, chronically ill, elderly, ...)	The findings from studies focusing solely on chronically ill patients are unambiguous: for patients with congestive heart failure, lipid disorders, diabetes, and schizophrenia, greater use of inpatient and emergency medical services was associated with higher cost-sharing for prescription drugs. "For certain conditions, the evidence clearly indicates that more cost sharing is associated with increased use of other medical services, such as hospitalizations and emergency department visits".<br>*Magnitude, chronically ill:* unclear<br>*Magnitude, chronically ill vs. non-chronically ill:* unclear

Gemmil, Thomson, Mossialos, 2008 [10]

Type of review/publication	- Narrative review<br>- Journal: International Journal for Equity in Health
Research question, overall	The impact of prescription drug charges on efficiency and equity.
Research question, specific to drug insurance/cost-sharing	Same.
Was an 'a priori' design provided?	No
Was there duplicate study selection and data extraction?	Unclear.
Was a comprehensive literature search performed?	Yes.<br>- databases: PubMed, EconLit, Blackwell's synergy, Ingenta;<br>- languages: English and non-English (did not specify which ones);<br>- year/month of last search: "until 2006";<br>- grey literature included: yes;<br>- keywords/search strategy: yes.
Search strategy, results	- Total number of studies included: 173<br>- Total number of studies, drugs/cost-sharing/insurance: 173<br>- Total number of Canadian studies: 28<br>• BC: Grootendorst, O'Brien et al., 1997; Grootendorst, Dolovich, et al., 2001; Narine, Senathirajah, Smith, 2001; Hazlet, Blough, 2002; Schneweiss, Maclure, Soumerai, 2002; Marshall, Grootendorst, et al., 2002; Schneeweiss, Dormuth, et al., 2004; Anis, Guh et al., 2005; Grootendorst, Marshall, et al., 2005; Grootendorst, Stewart, 2006; Schneweiss, Maclure, et al., 2006; Li, Guh et al., 2007;<br>• SK: Livingstone, Lix, et al., 2004;<br>• MB: Kozyrskyj, Mustard, Cheang, Simons, 2001;<br>• ON: Greenlick, Darsky, 1968; Hux, Naylor et al., 1997;<br>• QC: Blais, Castilloux et al., 1999; Poirier, LeLorier et al., 1998; Blais, Boucher et al., 2001; Tamblyn, Laprise et al., 2001; Pilote, Beck, et al., 2002; Blais, Couture et al., 2003; Contoyannis, Hurley et al., 2005;<br>• CA: Grootendorst, Levine, 2001; Alan, Crossley et al., 2002; Alan, Crossley et al., 2003; Smart, Stabile, 2005; Mabasa, Ma, 2006.
Was a list of studies (included and excluded) provided?	No, only references of included studies were reported; the number of studies excluded and the reasoning for their exclusion were provided, however, there is no way for the reader to trace the excluded studies.
Were the characteristics of the included studies provided?	To some extent: type of study (experimental study, natural study, observational study), type of data analyzed (cross-sectional data, time-series data, panel data), type of statistical analysis used (regression techniques; no regression techniques).
Was the scientific quality of the included studies assessed and documented?	No; 'quality' was assessed by looking at study design, type of data analyzed, and techniques used for analysis but did not assess quality beyond that.
Was the scientific quality of the included studies used appropriately in formulating conclusions?	To some extent; the quality of individual studies was generally discussed and taken to account.
Were the methods used to combine the findings of studies appropriate?	n/a

Was the likelihood of publication bias assessed?	n/a
Funding, conflicts of interest reported?	Funding:<br>- review: yes; Merck;<br>- included studies: no;<br>Conflict of interest:<br>- review: yes; no conflicts of interests reported;<br>- included studies: no.
Study's conclusion (as stated by the authors)	Using the interpretation of efficiency that focuses on improving health through the provision of effective health care, the cost, health, and distributional consequences of prescription drug charges can be seen to lower efficiency. Prescription drug charges are also likely to lower equity in the use of health care.
Limitations/risk of bias	- no 'a priori' design;<br>- no formal quality assessment of included studies;<br>- study characteristics of included studies not provided;<br>- list of excluded studies not provided;<br>- no/unclear duplicate study selection and data extraction.
Results - drug use (average effects)	– Demand for prescription drugs<br>Individuals who faced prescription drug charges were less likely to use prescription drugs; and those with insurance coverage were more likely to use them.<br>*Magnitude*: Overall, the demand for prescription drugs was almost always inelastic. Studies that used aggregate data generally found that a 10% increase in price resulted in a 0.6 to 8% decrease in use while studies that used individual- or household-level data generally found that a 10% increase in price resulted in a 0.2 to 6% decrease in use.<br>– Volume of drug use:<br>Most studies included found a negative relationship between prescription cost-sharing and levels of prescription drug use while insurance coverage had a positive effect on the volume of drug used;<br>*Magnitude*: unclear<br>– Brand-name vs generic drugs:<br>The demand for brand-name drugs is more price-elastic than that of generic drugs;<br>*Magnitude*: The demand for brand-name and the demand for generic drugs are both relatively inelastic.<br>- Essential and nonessential drug use:<br>*Direction*: Most studies found that prescription drug charges lowered the use of essential and non- essential drugs, although reductions in the use of non-essential drugs were usually slightly larger;<br>*Magnitude*: unclear
Results - drug use (by sub-groups such as SES, chronically ill, elderly, ...)	Older people were not found to be more sensitive to price than the general population.<br>*Magnitude, older adults*: A 10% increase in price led to changes in use for older people ranging from a 5.6% reduction to a 0.9% increase based on non-aggregate data, and one study using aggregate data found a reduction of 5.1%.<br>*Magnitude, older adults vs. non-older adults* : Among the general population, price elasticity estimates showed that a 10% increase in price led to a 0.2 to 4.6% decrease in use based on non-aggregate data and a 0.9 to 8.0% decrease in use based on aggregate data.<br><br>Poorer people were not found to be more sensitive to price than the general population;<br>*Magnitude, poor*: among the poor, a 10% increase in price led to reductions in use ranging from 0.3 to 2.0% based on non-aggregate data and 0.5 to 4.0% based on aggregate data.<br>*Magnitude, poor vs. non-poor*: among the general population, a 10% increase in price led to a 0.2 to 4.6% decrease in use based on non-aggregate data and a 0.9 to 8.0% decrease in use based on aggregate data;

Results - health outcomes	Overall, most studies that directly or indirectly considered the impact of prescription drug charges on health concluded that they lowered or were likely to lower health status because they led patients to forego the use of essential drugs, reduced adherence to treatment, and increased the likelihood of needing more intensive care and of dying;<br>*Magnitude of effect:* unclear
Results - health outcomes (by sub-groups such as SES, chronically ill, elderly, ...)	n/a
Results - healthcare services utilization (i.e., non-pharmaceutical services)	There was generally a positive relationship between prescription drug cost-sharing and outpatient, inpatient, and emergency care. Studies also found that prescription limits increased the frequency of partial hospitalization and nursing home admissions and the use of emergency mental health service. Two studies that found no effect were based on chronically ill patients;<br>*Magnitude:* unclear
Results - healthcare services utilization (i.e., non-pharmaceutical services) (by sub-groups such as SES, chronically ill, elderly, ...)	n/a

Remler, Greene, 2009 [11]

Type of review/publication	- Narrative review<br>- Journal: Annual Review of Public Health
Research question, overall	To determine the effects of cap and co-payment policies on rational use of medicines, healthcare utilization, health outcomes and costs
Research question, specific to drug insurance/cost-sharing	To determine the effects of cap and co-payment policies levied on prescription drugs on rational use of medicines, healthcare utilization, health outcomes, and costs.
Was an 'a priori' design provided?	No.
Was there duplicate study selection and data extraction?	Unclear.
Was a comprehensive literature search performed?	Unclear<br>- databases: not reported<br>- languages: not reported<br>- year/month of last search: not reported<br>- grey literature included: yes<br>- keywords/search strategy reported: no
Search strategy, results	unclear; not reported.<br>Section that examined prescription drug cost-sharing is based on Goldman, Joyce, Zheng (2007) and Gibson, Ozminkowski, Goetzel (2005) and a "a few important recent publications and working papers.
Was a list of studies (included and excluded) provided?	No. Only a reference list was provided.
Were the characteristics of the included studies provided?	No.
Was the scientific quality of the included studies assessed and documented?	No quality assessment of included studies. Research design is generally discussed.
Was the scientific quality of the included studies used appropriately in formulating conclusions?	To some extent; the quality of individual studies was generally discussed and taken into account.
Were the methods used to combine the findings of studies appropriate?	n/a
Was the likelihood of publication bias assessed?	n/a
Funding, conflicts of interest reported?	Funding:<br>- review: no<br>- included studies: no<br>Conflict of interest:<br>- review: yes<br>- included studies: no

Study's conclusion (as stated by the authors)	- On average, a 10% increase in pharmaceutical cost-sharing (measured as equivalent coinsurance) results in decreases of 2%–6% in pharmaceutical spending;<br>- There is mixed evidence that pharmaceutical cost-sharing affects more and less essential drugs differently;<br>- Pharmaceutical cost-sharing among those with chronic disease sometimes reduces the use of valuable drugs;<br>- There is a lack of direct evidence about pharmaceutical cost-sharing's effect on health.
Limitations/risk of bias	- no 'a priori' design;<br>- no/unclear duplicate study selection and data extraction;<br>- non-systematic search strategy;<br>- search strategy poorly described;<br>- unclear screening and data extraction process;<br>- list of included and excluded studies not provided;<br>- study characteristics of included studies not provided;<br>- no formal quality assessment of included studies.
Results - drug use (average effects)	There was an inverse association between pharmaceutical cost-sharing and pharmaceutical spending/use. There was mixed evidence that pharmaceutical cost-sharing affected essential drugs differently;<br>*Magnitude*: On average, a 10% increase in pharmaceutical cost-sharing (measured as equivalent coinsurance) resulted in decreases of 2%–6% in pharmaceutical spending/ use.
Results - drug use (by sub-groups such as SES, chronically ill, elderly, ...)	– Low-income<br>Evidence has not consistently shown a relationship between income and cost-sharing effects; the findings were mixed and not conclusive, and the work was limited by the relatively homogenous populations and proxy measures of income. In those cases in which lower-income individuals were more responsive to cost-sharing, both high-value care (such as mammogram use) and lower-value care were reduced;<br>*Magnitude, low-income*: unclear<br>*Magnitude, low-income vs. mid-, high-income*: unclear<br>– Chronically ill<br>Only a few studies compared the impact of cost-sharing on different health status groups; pharmaceutical cost-sharing among those with chronic disease sometimes reduced the use of valuable drugs; several studies conducted on chronically ill populations (including those with rheumatoid arthritis, heart failure, diabetes, schizophrenia, and lipid disorders) found unambiguous reductions in the use of drugs regarded as important for maintaining the health of the chronically ill;<br>*Magnitude, chronically ill*: unclear<br>*Magnitude, chronically ill vs. non-chronically ill*: unclear<br>- Very little empirical evidence has been reported on variation by race/ethnicity, age or gender.
Results - health outcomes	There was a lack of direct evidence about pharmaceutical cost-sharing's effect on health.<br>*Magnitude:* unclear.
Results - health outcomes (by sub-groups such as SES, chronically ill, elderly, ...)	n/a
Results - healthcare services utilization (i.e., non-pharmaceutical services)	Some evidence suggested that pharmaceutical cost-sharing increased emergency department use and hospitalizations. Limited evidence about resulting increases in outpatient care;<br>*Magnitude:* unclear

Results - healthcare services utilization (i.e., non-pharmaceutical services) (by sub-groups such as SES, chronically ill, elderly, ...)	Some studies examined a selective reduction in cost-sharing for selected important chronic medications and found significant increases in their use that might be associated with significant reductions in emergency room and hospital usage. Some evidence suggests that pharmaceutical cost-sharing increased emergency department use and hospitalizations. There was less evidence about increases in outpatient care;<br>*Magnitude, chronically ill:* unclear<br>*Magnitude, chronically ill vs. non-chronically ill:* unclear

Green, Maclure, et al., 2010 [12]

Type of review/publication	- Narrative review<br>- Report: The Cochrane Library
Research question, overall	To determine the effects of a pharmaceutical policy restricting the reimbursement of selected medications on drug use, health care utilization, health outcomes, and costs.
Research question, specific to drug insurance/cost-sharing	Same
Was an 'a priori' design provided?	Yes
Was there duplicate study selection and data extraction?	Yes
Was a comprehensive literature search performed?	Yes.<br>- databases: Cochrane Central Register of Controlled Trials (CENTRAL); MEDLINE; PubMed; EMBASE; Web of Science; Worldwide Political Science Abstracts; EconLit; International Political Science Abstracts; NHS Economic Evaluation Database; PAIS International; IPA, International Pharmaceutical Abstracts; Organization for Economic Co-operation and Development (OECD); World Bank e-Library/World Bank Documents & Reports;<br>- languages: no language restrictions;<br>- year/month of last search: January 2009;<br>- grey literature included: yes;<br>- keyword/search strategy reported: yes.
Search strategy, results	- Total number of studies included: 29<br>- Total number of studies included relevant to drug insurance/cost-sharing: 29<br>- Total Canadian studies: 11<br>• BC: Grootendorst, Marshall et al. 2005; Hazlet, Blough 2002; Marshall, Grootendorst et al. 2002; Marshall, Willison et al. 2007; Schneeweiss, Maclure, et al. 2004; Schneeweiss, Maclure, et al. 2006;<br>• ON: Jackevicious, Tu et al. 2008; Marshall, Gough et al. 2006; Marshall, Willison et al. 2007;<br>• NS: Kephart, Sketris et al. 2005; MacCara, Sketris et al. 2001;<br>• NL: Bursey, Crowley et al. 2000.
Was a list of studies (included and excluded) provided?	Yes
Were the characteristics of the included studies provided?	Yes; methods, participants, interventions, outcomes, notes
Was the scientific quality of the included studies assessed and documented?	Yes; included studies were appraised using the seven Cochrane EPOC criteria for interrupted time series: 1. Intervention independent of other changes; 2. The shape of the intervention pre-specified; 3. Intervention unlikely to affect data collection; 4. Knowledge of allocated interventions adequately prevented during the study; 5. Incomplete outcome data; 6. Free of selective reporting; 7. Free of other bias.
Was the scientific quality of the included studies used appropriately in formulating conclusions?	Yes

Were the methods used to combine the findings of studies appropriate?	n/a
Was the likelihood of publication bias assessed?	n/a
Funding, conflicts of interest reported?	Funding:<br>- review: yes; University of Victoria; British Colombia Medical Association; Canadian Institutes of Health Research;<br>- included studies: no<br>Conflict of interest:<br>- review: yes; Ministry of Health Services of British Columbia; Norwegian Medicines Agency;<br>- included studies: no
Study's conclusion (as stated by the authors)	Implementing restrictions to coverage and reimbursement of selected medications can decrease third party drug spending without increasing the use of other health services (6 studies). Relaxing reimbursement rules for drugs used for secondary prevention can also remove barriers to access.
Limitations/risk of bias	- restrictive inclusion criteria may limit the usefulness of the review.
Results - drug use (average effects)	Restriction to reimbursement decreased drug use, either immediately after policy implemented or long-term. Impact varied by drug class and whether restrictions were implemented or relaxed;<br>*Magnitude*: unclear
Results - drug use (by sub-groups such as SES, chronically ill, elderly, ...)	n/a
Results - health outcomes	Only two of the studies included reported health outcome data, precluding any conclusions about the impact of prior authorization policies on patient outcomes.<br>*Magnitude:* unclear
Results - health outcomes (by sub-groups such as SES, chronically ill, elderly, ...)	n/a
Results - healthcare services utilization (i.e., non-pharmaceutical services)	The effects of pharmaceutical reimbursement on health care access were uncertain. Some studies reported an immediate increase in utilization while another found no significant difference in office visits, hospitalization, or length of stay. Very few studies looked at the long-term impact on utilization. One study reported an increase in outpatient services but no change in inpatient and long-term services;<br>*Magnitude:* unclear
Results - healthcare services utilization (i.e., non-pharmaceutical services) (by sub-groups such as SES, chronically ill, elderly, ...)	n/a

Holst, 2010 [13]

Type of review/publication	- Narrative review<br>- Working paper: Wissenschaftszentrum Berlin für Sozialforschung (WZB)
Research question, overall	Does direct patient cost-sharing improve the efficiency of use of resources in health care? What effects does it have on social inequality of health opportunities in the population, and the political goal of reducing this? What do the existing studies on this subject have to say, and how relevant are they to the realities of healthcare? What conclusions can be drawn concerning the commonplace ideas of health economics?
Research question, specific to drug insurance/cost-sharing	Does direct patient pharmaceutical cost-sharing affect pharmaceutical use, non-pharmaceutical healthcare services, and health outcomes?
Was an 'a priori' design provided?	No.
Was there duplicate study selection and data extraction?	Unclear.
Was a comprehensive literature search performed?	Unclear.<br>- databases: not reported;<br>- languages: not reported;<br>- year/month of last search: not reported;<br>- grey literature included: yes;<br>- keyword/search strategy reported: no.
Search strategy, results	- Total number of studies included: not clearly reported;<br>- Total number of studies included relevant to drug insurance/cost-sharing: not clearly reported;<br>- Total Canadian studies: not clearly reported.
Was a list of studies (included and excluded) provided?	No
Were the characteristics of the included studies provided?	No
Was the scientific quality of the included studies assessed and documented?	No
Was the scientific quality of the included studies used appropriately in formulating conclusions?	To some extent; the quality of individual studies was generally discussed and taken into account.
Were the methods used to combine the findings of studies appropriate?	n/a
Was the likelihood of publication bias assessed?	n/a

Funding, conflicts of interest reported?	Funding:<br>- review: no;<br>- included studies: no;<br>Conflict of interest:<br>- review: no;<br>- included studies: no.
Study's conclusion (as stated by the authors)	Increased cost sharing was associated with lower rates of drug treatment, worse adherence among existing users and more frequent discontinuation of therapy. For each 10% increase in cost-sharing, prescription drug spending decreased by 2-6% (depending on class of drug and condition of patient). For certain chronic conditions, higher cost-sharing was associated with increased use of medical services (those with congestive heart failure, schizophrenia, diabetes, lipid disorders).
Limitations/risk of bias	- no 'a priori' design;<br>- search strategy poorly described;<br>- unclear screening and data extraction process (inclusion and exclusion criteria not stated);<br>- list of included and excluded studies not provided;<br>- study characteristics of included studies not provided;<br>- no formal quality assessment of included studies.
Results - drug use (average effects)	Consistent findings that increasing prescription cost-sharing reduced drug use and patient compliance to drug therapies. Effect varied depending on class of substance;<br>*Magnitude*: unclear
Results - drug use (by sub-groups such as SES, chronically ill, elderly, ...)	– Elderly<br>Older people responded especially sensitively to cost-sharing.<br>*Magnitude, older adults*: unclear<br>*Magnitude, older adults vs. non-older adults*: unclear<br>– Low-income<br>Some evidence that lower-income individuals were sensitive to increased cost-sharing;<br>*Magnitude, subgroup*: unclear<br>*Magnitude, low-income vs. mid-, high-income*: unclear<br>– Chronically ill<br>Cost-induced non-adherence to medical recommendations was observed more often among people who needed treatment than among healthy citizens;<br>*Magnitude, chronically ill*: unclear<br>*Magnitude, chronically ill vs. non-chronically ill*: unclear
Results - health outcomes	n/a
Results - health outcomes (by sub-groups such as SES, chronically ill, elderly, ...)	n/a
Results - healthcare services utilization (i.e., non-pharmaceutical services)	n/a
Results - healthcare services utilization (i.e., non-pharmaceutical services) (by sub-groups such as SES, chronically ill, elderly, ...)	With certain chronic conditions, an increase in drug co-payments led to increased use of other medical services such as consulting practitioners and hospital admissions;<br>*Magnitude, chronically ill:* unclear<br>*Magnitude, chronically ill vs. non-chronically ill:* unclear

Polinski, Kilabuk, et al., 2010 [14]

Type of review/publication	- Narrative review<br>- Journal: Journal of the American Geriatrics Society
Research question, overall	To assess the extent to which Medicare Part D's cost-sharing provisions and drug coverage rules affected the under- and overuse of specific drugs and classes.
Research question, specific to drug insurance/cost-sharing	Same
Was an 'a priori' design provided?	No
Was there duplicate study selection and data extraction?	Yes
Was a comprehensive literature search performed?	No.<br>- databases: Medline;<br>- languages: not reported;<br>- year/month of last search: October 2009;<br>- grey literature included: no;<br>- keywords/search strategy reported: yes.
Search strategy, results	- Total number of studies included: 26<br>- Total number of studies, drugs/cost-sharing/insurance: 26<br>- Total number of Canadian studies: 0
Was a list of studies (included and excluded) provided?	No; only list of the included studies was provided.
Were the characteristics of the included studies provided?	Yes; research question; data source; beneficiary characteristics; study design; drugs studied; results.
Was the scientific quality of the included studies assessed and documented?	Yes; the Newcastle-Ottawa Scale for cohort studies was used. However, the scale was not described and only total scores were reported.
Was the scientific quality of the included studies used appropriately in formulating conclusions?	To some extent; the quality of individual studies was generally discussed and taken into account.
Were the methods used to combine the findings of studies appropriate?	n/a
Was the likelihood of publication bias assessed?	n/a
Funding, conflicts of interest reported?	Funding:<br>- review: yes; CVS Caremark, National Institute on Aging; National Institute of Mental Health; National Heart, Lung, and Blood Institute;<br>- included studies: no;<br>Conflict of interest:<br>- review: yes; HealthCore; World Health Information Science; Pfizer; CVS/Caremark; Express Scripts;<br>- included studies: no.

Study's conclusion (as stated by the authors)	Introducing Part D led to greater use of essential medicines especially in beneficiaries who had been previously uninsured, and of nonessential medicines. In the Part D transition period, dually eligible beneficiaries' drug use remained largely unchanged. When beneficiary cost-sharing increased in the coverage gap, use of essential and overused medications declined.
Limitations/risk of bias	- no 'a priori' design;<br>- non-systematic search strategy;<br>- grey literature not searched;<br>- unclear exclusion criteria;<br>- list of excluded studies not provided;<br>- formal quality assessment of included studies poorly described and discussed; only global ratings provided; unclear how any of the domains were operationalized and assessed.
Results - drug use (average effects)	n/a
Results - drug use (by sub-groups such as SES, chronically ill, elderly, ...)	The inception of Medicare Part D was associated with a consistent overall increase in drug use. There was little variation in effect estimates between studies evaluating the effect of Part D implementation. Across all studies, entry of Part D beneficiaries into the coverage gap was associated with reduced drug use;<br>*Magnitude, seniors*: The inception of Part D was associated with a 6% to 13% increase in drug use. Changes in use varied according to drug, disease, and population studied. There was little indication that Part D selectively led to greater use of essential, underused drugs than of overused medications. Across all studies, entry of Part D beneficiaries into the coverage gap was associated with 9% to 16% less drug use. Patients who entered the coverage gap were 5% to 11% more likely to report discontinuing, switching, or failing to initiate a medication than were patients who did not enter the coverage gap. Use of generic drugs increased 20% during the coverage gap.<br>*Magnitude, seniors vs. non-seniors*: unclear
Results - health outcomes	n/a
Results - health outcomes (by sub-groups such as SES, chronically ill, elderly, ...)	n/a
Results - healthcare services utilization (i.e., non-pharmaceutical services)	n/a
Results - healthcare services utilization (i.e., non-pharmaceutical services) (by sub-groups such as SES, chronically ill, elderly, ...)	n/a

Polinski, Donohue, et al, 2011 [15]

Type of review/publication	- Narrative review;<br>- Journal: Journal of the American Geriatrics Society
Research question, overall	To assess the extent to which Part D's cost-sharing provisions and drug coverage rules affected the under-and overuse of specific drugs and classes
Research question, specific to drug insurance/cost-sharing	Same.
Was an 'a priori' design provided?	No
Was there duplicate study selection and data extraction?	Yes
Was a comprehensive literature search performed?	- No.<br>- databases: Medline<br>- languages: not mentioned<br>- year/month of last search: October 2010<br>- grey literature included: no<br>- keyword/search strategy: yes
Search strategy, results	- Total number of studies included: 19<br>- Total number of studies included relevant to drug insurance/cost-sharing: 19<br>- Total Canadian studies: 0
Was a list of studies (included and excluded) provided?	No. Only the included studies are provided
Were the characteristics of the included studies provided?	Yes; authors, research question; data source; beneficiary characteristics; study design; drugs studied; results.
Was the scientific quality of the included studies assessed and documented?	Yes; the Newcastle-Ottawa Scale for cohort studies was used. However, the scale was not described and only total scores were reported.
Was the scientific quality of the included studies used appropriately in formulating conclusions?	To some extent; limitations of studies described in general in discussion section
Were the methods used to combine the findings of studies appropriate?	n/a
Was the likelihood of publication bias assessed?	n/a
Funding, conflicts of interest reported?	Funding:<br>- review: yes; National Heart Lung and Blood Institute Grant; CVS/Caremark;<br>- included studies: no;<br>Conflict of interest:<br>- review: yes; CVS/Caremark; Express Scripts;<br>- included studies: no.

Study's conclusion (as stated by the authors)	Introducing Medicare Part D led to greater use of essential medicines especially in beneficiaries who had been previously uninsured, and of nonessential medicines. In the Part D transition period, dually eligible beneficiaries' drug use remained largely unchanged. When beneficiary cost-sharing increased in the coverage gap, use of essential and overused medications declined.
Limitations/risk of bias	- no 'a priori' design;<br>- unclear inclusion/exclusion criteria;<br>- non-systematic search strategy;<br>- search strategy poorly described;<br>- grey literature not searched;<br>- formal quality assessment of included studies poorly described and discussed; only global ratings provided; unclear how any of the domains were operationalized and assessed.
Results - drug use (average effects)	n/a
Results - drug use (by sub-groups such as SES, chronically ill, elderly, ...)	In the period after Medicare Part D implementation there was an increase in the use of essential medicines especially in beneficiaries who had been previously uninsured, and of nonessential medicines. During the transition period, dually eligible beneficiaries' drug use remained largely unchanged. In the coverage gap, when cost-sharing increased, the use of essential and overused medications declined;<br>*Magnitude, seniors*: unclear<br>*Magnitude, seniors vs. non-seniors*: unclear
Results - health outcomes	n/a
Results - health outcomes (by sub-groups such as SES, chronically ill, elderly, ...)	n/a
Results - healthcare services utilization (i.e., non-pharmaceutical services)	n/a
Results - healthcare services utilization (i.e., non-pharmaceutical services) (by sub-groups such as SES, chronically ill, elderly, ...)	n/a

Swartz, 2010 [16]

Type of review/publication	- Narrative review<br>- The Synthesis Project (The Robert Wood Johnson Foundation)
Research question, overall	What is known and unknown about the effects of consumer cost sharing?
Research question, specific to drug insurance/cost-sharing	1. What are the effects of cost-sharing on the distribution of spending and total spending?<br>2. What are the effects of increased cost-sharing on health outcomes?<br>3. How do responses to cost-sharing differ by socioeconomic factors and health status?<br>4. What is the effect of cost-sharing on different types of services?<br>5. What are the effects of increased cost-sharing for prescription drugs?
Was an 'a priori' design provided?	No.
Was there duplicate study selection and data extraction?	No.
Was a comprehensive literature search performed?	No.<br>- databases: unclear;<br>- languages: unclear<br>- year/month of last search: unclear<br>- grey literature included: unclear<br>- keywords/search strategy reported: no
Search strategy, results	- Total number of studies included: unclear<br>- Total number of studies, drugs/cost-sharing/insurance: unclear<br>- Total number of Canadian studies: unclear
Was a list of studies (included and excluded) provided?	No, only references of included studies are provided.
Were the characteristics of the included studies provided?	No.
Was the scientific quality of the included studies assessed and documented?	No formal quality assessment conducted. However, in general, greater weight is given to studies that used data from natural experiments with credible comparison groups, as well as studies that relied on data from larger numbers of people and from people who were representative of subgroups of people.
Was the scientific quality of the included studies used appropriately in formulating conclusions?	n/a
Were the methods used to combine the findings of studies appropriate?	n/a

Was the likelihood of publication bias assessed?	n/a
Funding, conflicts of interest reported?	Funding:<br>- review: no<br>- included studies: no<br>Conflict of interest:<br>- review: no<br>- included studies: no
Study's conclusion (as stated by the authors)	The demand for most health care services is price sensitive. When people have to pay more for health care, they reduce their use of health care.<br><br>Low-income people are at greater risk than higher-income people in terms of poor health outcomes due to increased cost-sharing. This conclusion appears to be especially pertinent for low-income elderly with chronic conditions and low- to middle-income people with mental health diagnoses that are treatable with prescription drugs. We do not know if increased patient cost-sharing would reduce the growth in total national health care spending. In general, most people do not distinguish between health care services or prescription drugs that are essential and those that are not essential.
Limitations/risk of bias	- no 'a priori' design;<br>- list of excluded studies not provided;<br>- no/unclear duplicate study selection and data extraction;<br>- search strategy poorly described;<br>- poorly justified or unclear exclusion criteria;<br>- study characteristics of included studies not provided;<br>- no quality assessment of included studies;
Results - drug use (average effects)	Increased cost-sharing for prescription drugs is associated with decline in use and spending on drugs. The evidence is unclear whether people respond to increased cost-sharing by switching to less expensive, close drug substitutes. With increased cost-sharing, both essential and non-essential drug use is decreased but the decrease is larger for nonessential drugs.<br>*magnitude*: Increased cost-sharing of about 10 percent is associated with a decline of between 1 percent and 6 percent in spending on prescription drugs.
Results - drug use (by sub-groups such as SES, chronically ill, elderly, ...)	Cost-sharing reduced use of essential drugs in people with chronic conditions and the elderly. Studies that look at the Medicare doughnut hole found that elderly reduce drug use when they have to pay full price. A recent study examined changes in prescription drug co-payments imposed on privately insured people and how the effects were different for people living in low-income areas compared with high-income areas. Privately insured people who did not have the increased co-payments were a comparison group. The results indicate that for each medication class examined, individuals living in high-income areas were consistently more likely to continue taking their medications than people in low-income areas.<br><br>*magnitude, elderly:* One study in the elderly found cost-sharing reduced essential drugs by 9.1% for essential drugs and 15.1% for nonessential drugs.<br>*magnitude, elderly vs non-elderly:* unclear<br>*magnitude, poor:* unclear<br>*magnitude, poor vs non-poor:* unclear
Results - health outcomes	There are very few studies that look at the effect of cost-sharing on health. As such, long-term health effects of reduced use of essential drugs especially people with chronic health conditions is unknown.<br>*magnitude:* unclear

Results - health outcomes (by sub-groups such as SES, chronically ill, elderly, ...)	It is suggested that low-income people are at greater risk than higher income people in terms of poor health outcomes due to increased cost-sharing.<br>*magnitude, low income:* unclear<br>*magnitude, low income vs high income:* unclear
Results - healthcare services utilization (i.e., non-pharmaceutical services)	N/A
Results - healthcare services utilization (i.e., non-pharmaceutical services) (by sub-groups such as SES, chronically ill, elderly, ...)	Increased cost-sharing for prescription drugs appears to cause increased expenditures on emergency department services and inpatient hospitalizations by elderly and welfare beneficiaries. Tamblyn et al., found that after Quebec implemented a significant increase in cost-sharing for prescription drugs, there was a significant increase in emergency department (ED) visits, hospitalizations, and admissions to a long-term- care facility due to people reducing their use of essential drugs. Another study found higher rates of hospitalizations and death among Medicare beneficiaries who faced a cap on their pharmacy benefits under a Medicare+Choice plan compared with beneficiaries in the same Medicare+Choice plan who did not have a cap on pharmacy benefits. Another study argues that the evidence is unambiguous for people with chronic illnesses that higher cost-sharing leads to greater use of hospital inpatient and emergency department services, presumably because the people cut back on their use of essential drugs. Low-income people in poor health are more likely to suffer adverse health outcomes, such as increased rates of emergency department (ED) use, hospitalizations, admission to nursing homes when increased cost-sharing causes them to reduce their use of health care, particularly prescription drugs.<br>*magnitude, elderly:* unclear<br>*magnitude elderly vs non-elderly*: unclear<br>*magnitude, chronically ill:* unclear<br>*magnitude, chronically ill vs non-chronically ill:* unclear<br>*magnitude, low income:* unclear<br>*magnitude, low income vs high income:* unclear

Baicker, Goldman, 2011 [17]

Type of review/publication	- Narrative review<br>- Journal: Journal of Economic Perspectives
Research question, overall	To determine the relationship between patient cost-sharing and healthcare spending growth.
Research question, specific to drug insurance/cost-sharing	The effects of cost-sharing on drug utilization and health outcomes.
Was an 'a priori' design provided?	No.
Was there duplicate study selection and data extraction?	Unclear.
Was a comprehensive literature search performed?	Unclear.<br>- databases: not reported;<br>- languages: not reported;<br>- year/month of last search: not reported;<br>- grey literature included: not reported;<br>- keywords/search strategy reported: no.
Search strategy, results	- Total number of studies included: not reported;<br>- Total number of studies, drugs/cost-sharing/insurance: not reported;<br>- Total number of Canadian studies: 1<br>• QC: Tamblyn, Laprise et al. 2001.
Was a list of studies (included and excluded) provided?	No
Were the characteristics of the included studies provided?	No
Was the scientific quality of the included studies assessed and documented?	No
Was the scientific quality of the included studies used appropriately in formulating conclusions?	No
Were the methods used to combine the findings of studies appropriate?	n/a
Was the likelihood of publication bias assessed?	n/a
Funding, conflicts of interest reported?	Funding:<br>- review: no;<br>- included studies: no;<br>Conflict of interest:<br>- review: no;<br>- included studies: no.

Study's conclusion (as stated by the authors)	In pharmaceuticals, the earliest studies on the link between cost-sharing and use find relatively modest effects, but they focus on small changes in copayments. More recent work indicates that the responses to increased patient cost-sharing are stronger. Overall, these studies suggest a price elasticity for drug expenditures of 0.2 to 0.6, similar to results from the RAND Health Insurance Experiment.
Limitations/risk of bias	- no 'a priori' design;<br>- no/unclear duplicate study selection and data extraction;<br>- search strategy not described;<br>- list of included and excluded studies not provided;<br>- no quality assessment of included studies;<br>- study characteristics of included studies not provided;<br>- focus on US studies limits the usefulness of the review.
Results - drug use (average effects)	Higher cost-sharing was negatively associated with the demand for prescription drugs;<br>*Magnitude*: The evidence suggest a price elasticity for drug expenditures of -0.2 to -0.6. The range reflects differences in responsiveness by drug class and its importance.
Results - drug use (by sub-groups such as SES, chronically ill, elderly, ...)	One study that examined Medicare Part D found that providing insurance to the elderly led to increased prescription drug use;<br>*Magnitude, seniors*: providing insurance to the elderly led to a 13% increase in prescription drug use. Further interpretation not provided<br>*Magnitude, seniors vs. non-seniors*: unclear
Results - health outcomes	Evidence on the ultimate effect of cost-sharing on health outcomes was sparse. Most studies did not examine the effect of coinsurance on health directly. Existing evidence suggested that increased out-of-pocket costs led to lower compliance of drug use, which may indirectly have led to poorer health;<br>*Magnitude:* unclear
Results - health outcomes (by sub-groups such as SES, chronically ill, elderly, ...)	Adverse health consequences of cost-sharing (unclear if drug cost-sharing only) have been found for patients with congestive heart failure, lipid disorders, diabetes, and schizophrenia.<br>*Magnitude, chronically ill:* unclear<br>*Magnitude, chronically ill vs. non-chronically ill:* unclear
Results - healthcare services utilization (i.e., non-pharmaceutical services)	The authors conclude that there were substantial cross-price effects from changing copayments for pharmaceuticals. Increasing drug copayments in private insurance plans led to increases in other medical spending. This conclusion, however, was supported by only two studies.
Results - healthcare services utilization (i.e., non-pharmaceutical services) (by sub-groups such as SES, chronically ill, elderly, ...)	n/a

Eaddy, Cook, et al., 2012 [18]

Type of review/publication	- Narrative review.<br>- Journal: Pharmacy and Therapeutics
Research question, overall	To assess the relationship between patient cost-sharing, medication adherence, clinical, utilization, and economic outcomes.
Research question, specific to drug insurance/cost-sharing	Same
Was an 'a priori' design provided?	No
Was there duplicate study selection and data extraction?	Unclear
Was a comprehensive literature search performed?	Yes.<br>- databases: PubMed; Ovid; Medline; Web of Science; Google Scholar;<br>- languages: English;<br>- year/month of last search: May 2008;<br>- grey literature included: no;<br>- keywords/search strategy reported: yes.
Search strategy, results	- Total number of studies included: 160<br>- Total number of studies, drugs/cost-sharing/insurance: 160<br>- Total number of Canadian studies: 17<br>• BC: Anis, Guh, et al. 2005; Dormuth, Glynn, et al. 2006; Schneeweiss, Maclure, et al.2002; Schneeweiss, Patrick, et al. 2007-Circulation; Schneeweiss, Patrick, et al. 2007-Am J Manag Care;<br>• SK: Suissa, Ernst, Kezouh, 2002; Blackburn, Dobson et al. 2005;<br>• ON: Ungar, Kozyrskyj, et al. 2008; Jackevicius, Li, et al. 2008; Rasmussen, Chong, Alter, 2007;<br>• QC: Tamblyn, Laprise, et al. 2001; Blais, Boucher, et al. 2001; Pilote, Beck, et al. 2002; Blais, Couture, et al. 2003; Bouchard, Dragomir, et al. 2007<br>• NS: Kephart, Skedgel, et al. 2007<br>• Canada: Turner, Wright, et al. 1995.
Was a list of studies (included and excluded) provided?	No. Only the included studies are provided.
Were the characteristics of the included studies provided?	No; characteristics of included studies provided for only a subset of 24 studies (year of publication, medication class, population, changes in cost-sharing, change in adhererence0.
Was the scientific quality of the included studies assessed and documented?	No
Was the scientific quality of the included studies used appropriately in formulating conclusions?	To some extent; limitations of studies described in general.
Were the methods used to combine the findings of studies appropriate?	n/a
Was the likelihood of publication bias assessed?	n/a

Was the conflict of interest included?	Funding:<br>- review: yes; GlaxoSmithKline<br>- included studies: no<br>Conflict of interest:<br>- review: yes; GlaxoSmithKline<br>- included studies: no
Study's conclusion (as stated by the authors)	Increasing patient cost-sharing was associated with declines in medication adherence, which in turn was associated with poorer health outcomes.
Limitations/risk of bias	- no 'a priori' design;<br>- unclear duplicate study selection and data extraction;<br>- search strategy poorly described;<br>- search limited to English;<br>- grey literature not searched;<br>- unclear screening and data extraction process (inclusion and exclusion criteria not clearly stated);<br>- list of excluded studies not provided;<br>- no formal quality assessment of included studies;<br>- study characteristics of included studies not provided.
Results - drug use (average effects)	Most studies found a statistically significant relationship between increased patient drug cost-sharing and decreased medication adherence. The effect depended on the population and intervention;<br>*Magnitude*: overall, a $10 increase was associated with a 3.8% decrease in adherence.
Results - drug use (by sub-groups such as SES, chronically ill, elderly, ...)	n/a
Results - health outcomes	Most studies indicated that increased patient drug cost-sharing adversely affected health outcomes (outcomes included adverse events, self-reported health status, and symptoms). A few studies found no effect on outcomes and no effect of adherence, supporting the hypothesis that the effect of cost sharing on outcomes is mediated through adherence;<br>*Magnitude:* unclear
Results - health outcomes (by sub-groups such as SES, chronically ill, elderly, ...)	n/a
Results - healthcare services utilization (i.e., non-pharmaceutical services)	Most studies indicated that increased patient drug cost-sharing adversely affected health services utilization (ED visits, outpatient visits, preventative services, hospitalizations and nursing-home admissions). Fewer studies indicated that an increase in cost-sharing did not affect medical utilization or number of medical visits;<br>*Magnitude:* unclear
Results - healthcare services utilization (i.e., non-pharmaceutical services) (by sub-groups such as SES, chronically ill, elderly, ...)	n/a

Lemstra, Blackburn et al., 2012 [19]

Type of review/publication	- Meta-analysis<br>- Journal: Canadian Journal of Cardiology
Research question, overall	To quantify the proportion of adherence to statin medications by study design (randomized trial vs observational study); and to provide estimates of risk indicators associated with nonadherence to statin medications.
Research question, specific to drug insurance/cost-sharing	Association between co-payment and adherence to statin medications.
Was an 'a priori' design provided?	No
Was there duplicate study selection and data extraction?	Unclear; methodological quality evaluation by a panel of 3 reviewers.
Was a comprehensive literature search performed?	Yes.<br>- databases: PubMed; PsychINFO; CINAHL); Cochrane Central; DARE; NHSEED; HTAD;Embase;<br>- languages: English only;<br>- year/month of last search: June 2011;<br>- grey literature included: no;<br>- keywords/search strategy reported: yes.
Search strategy, results	- Total number of studies included: 67<br>- Total number of studies, drugs/cost-sharing/insurance: 6<br>- Total number of Canadian studies: 1<br>• BC: Schneeweiss, Patrick et al. 2007 - Circulation
Was a list of studies (included and excluded) provided?	No; list of excluded studies not provided.
Were the characteristics of the included studies provided?	Yes; year/country, adherence measure/data source, % adherent, factors associated with non-adherence, odds ratio (95% CI), factors not associated with non-adherence.

Was the scientific quality of the included studies assessed and documented?	Yes;<br>RCT Appraisal:<br>1. Was a method of randomization performed?<br>2. Was the treatment allocation concealed?<br>3. Were the groups similar at baseline regarding the most important prognostic indicators?<br>4. Were the eligibility criteria specified?<br>5. Was the outcome assessor blinded?<br>6. Was the care provider blinded?<br>7. Was the patient blinded?<br>8. Were point estimates (confidence intervals and/or odds ratios) and measures of variability presented for the primary outcome measures?<br>9. Did the analysis include an intention-to-treat analysis?<br><br>Observational Trial Appraisal<br>1. Was the selection process for patient enrollment specified?<br>2. Were the patients uniformly identified at presentation (i.e., at the same stage in disease progression?)<br>3. Were the criteria for inclusion and exclusion specified?<br>4. Was any comparative information obtained for the patients who were not enrolled in the study?<br>5. Were all the patients who were initially entered into the study accounted for in the results?<br>6. Were the characteristics of patients lost to follow up been described? (Yes – little or zero losses to follow up). 7. Were any statistical tests used?<br>8. Was adjustment for extraneous prognostic factors (confounders) carried out?<br><br>A score of 5 out of 9 and 5 out of 8 was required for a randomized trial and [illegible]
Was the scientific quality of the included studies used appropriately in formulating conclusions?	Yes
Were the methods used to combine the findings of studies appropriate?	Yes
Was the likelihood of publication bias assessed?	No
Funding, conflicts of interest reported?	Funding:<br>- review: yes; Ministry of Health, Saskatchewan; Merck Frosst/Schering Pharmaceuticals, AstraZeneca Canada, Pfizer Canada;<br>- included studies: no<br>Conflict of interest:<br>- review: yes<br>- included studies: no
Study's conclusion (as stated by the authors)	Among 6 studies with a total sample size of 884,643, patients required to make a copayment when their statin medications were dispensed were 28% more likely than others to be nonadherent (rate ratio ? 1.28; 95% CI, 1.09-1.50).
Limitations/risk of bias	- no 'a priori' design;<br>- no/unclear duplicate study selection and data extraction;<br>- search limited to English;<br>- grey literature not searched;<br>- list of excluded studies not provided;<br>- arbitrary threshold used to categorize the quality of included studies.

Results - drug use (average effects)	n/a
Results - drug use (by sub-groups such as SES, chronically ill, elderly, ...)	Statin users required to make a copayment were more likely than others to be non-adherent;<br>*Magnitude, statin users* : Among six studies with a total sample size of 884,643, patients required to make a copayment when their statin medications were dispensed were 28% more likely than others to be non-adherent (rate ratio 1.3; 95%CI, 1.1-1.5).<br>*Magnitude, statin users vs. non-statin users*: unclear
Results - health outcomes	n/a
Results - health outcomes (by sub-groups such as SES, chronically ill, elderly, ...)	n/a
Results - healthcare services utilization (i.e., non-pharmaceutical services)	n/a
Results - healthcare services utilization (i.e., non-pharmaceutical services) (by sub-groups such as SES, chronically ill, elderly, ...)	n/a

Sinnott, Buckley, et al., 2013 [20]

Type of review/publication	- Meta-analysis<br>- Journal: PLOS One
Research question, overall	The effect of copayments for prescriptions on adherence to prescription medicines in publicly insured populations.
Research question, specific to drug insurance/cost-sharing	Same.
Was an 'a priori' design provided?	No
Was there duplicate study selection and data extraction?	Yes
Was a comprehensive literature search performed?	Yes<br>- databases: PubMed, Medline(Ovid), Cinahl, EMBASE, EconLit, SCOPUS, Web of Knowledge, Cochrane Library;<br>- languages: no restrictions<br>- year/month of last search: September 2012<br>- grey literature included: yes;<br>- keywords/search strategy reported: yes.
Search strategy, results	- Total number of studies included: 7<br>- Total number of studies, drugs/cost-sharing/insurance: 7<br>- Total number of Canadian studies: 0
Was a list of studies (included and excluded) provided?	Yes
Were the characteristics of the included studies provided?	Yes; author/year, setting, sample size and characteristics, type of study, adherence measurement, intervention, follow up, result.
Was the scientific quality of the included studies assessed and documented?	Yes.<br>- Controlled before-and-after (CBA) studies and interrupted time series designs (ITS) were assessed for quality/risk of bias using a modified version of the EPOC Data Collection Checklist and Quality Criteria for CBA and ITS; studies were rated as strong, moderate, weak or fatally flawed (''weak'' = two or more unmet criteria. Criteria: 1. baseline measurement; 2. characteristics of studies using second site as control; 3. confounding; 4. blinded assessment of primary outcome; 5. protection against contamination; 6. reliable primary outcome measure; 7. Other risks of bias; 8.follow up patient/attrition;<br>- Cohort studies were assessed using the Effective Public Health Practice Project component rating scale; studies were rated as strong, moderate or weak ("weak" = two weak ratings across the criteria). Criteria: 1. selection bias; 2. allocation bias; 3.confounding; 4.Blinding; 5. data collection objective; 6.Attrition bias; 7.intervention integrity; 8.statistics.<br>Only global ratings provided; 6/7 studies rated as weak, 1 study rated as weak/moderate.
Was the scientific quality of the included studies used appropriately in formulating conclusions?	Yes
Were the methods used to combine the findings of studies appropriate?	Yes. Statistical heterogeneity was assessed using the I2 test for heterogeneity. Findings were pooled using random effects models.
Was the likelihood of publication bias assessed?	Yes. Visual inspection of a funnel plot suggested that publication bias may be present.

Funding, conflicts of interest reported?	Funding:<br>- review: yes; Health Research Board, Ireland<br>- included studies: no;<br>Conflict of interest:<br>- review: yes;<br>- included studies: no.
Study's conclusion (as stated by the authors)	This meta-analysis showed an 11% increased odds of non-adherence to medicines in publicly insured populations where copayments for medicines are necessary.
Limitations/risk of bias	- no 'a priori' design;<br>- quality assessment: only global ratings provided; unclear how any of the domains were operationalized and assessed;<br>- arbitrary threshold used to categorize the quality of included studies;<br>- small number of included studies limits the generalizability of the findings
Results - drug use (average effects)	There was a positive association between co-payments and non-adherence;<br>*Magnitude*: summary odds ratio for non-adherence was 1.11 (95% CI 1.09–1.14); publicly insured patients who were required to pay copays for their prescription medicines had 11% higher odds of reporting non-adherence relative to those who faced no co-payments.
Results - drug use (by sub-groups such as SES, chronically ill, elderly, ...)	n/a
Results - health outcomes	n/a
Results - health outcomes (by sub-groups such as SES, chronically ill, elderly, ...)	n/a
Results - healthcare services utilization (i.e., non-pharmaceutical services)	n/a
Results - healthcare services utilization (i.e., non-pharmaceutical services) (by sub-groups such as SES, chronically ill, elderly, ...)	n/a

Maimaris, Paty, et al., 2013 [21]

Type of review/publication	- Narrative review.<br>- journal: Plos One
Research question, overall	What is the influence of national or regional health systems on hypertension awareness, treatment and control?
Research question, specific to drug insurance/cost-sharing	What is the effect of health systems financing on hypertension outcomes/ medication adherence?
Was an 'a priori' design provided?	Yes
Was there duplicate study selection and data extraction?	Yes
Was a comprehensive literature search performed?	Yes.<br>- databases: MEDLINE, Embase, Global Health, LILACS, Africa-Wide Information, IMSEAR, IMEMR, WPRIM);<br>- languages: no restrictions<br>- year/month of last search: May 2013;<br>- grey literature included: no;<br>- keywords/search strategy reported: yes.
Search strategy, results	- Total number of studies included: 53<br>- Total number of studies, drugs/cost-sharing/insurance: 35<br>- Total number of Canadian studies: 0
Was a list of studies (included and excluded) provided?	No; list of excluded studies not provided.
Were the characteristics of the included studies provided?	Yes. Study, setting and sample size, study design and length of follow-up, findings, risk of bias assessment
Was the scientific quality of the included studies assessed and documented?	Yes. For observational study designs, risk of bias was assessed using three domains: selection bias, information bias, and confounding. For RCTs, the Cochrane risk of bias tool was used.
Was the scientific quality of the included studies used appropriately in formulating conclusions?	Yes
Were the methods used to combine the findings of studies appropriate?	n/a
Was the likelihood of publication bias assessed?	n/a
Funding, conflicts of interest reported?	Funding:<br>- review: yes; Canadian Institutes of Health Research;<br>- included studies: no;<br>Conflict of interest:<br>- review: yes; no conflicts of interests reported;<br>- included studies: no.

Study's conclusion (as stated by the authors)	There was an association between reduced co-payments for health care, including for medications, and improved outcomes of hypertension care in multiple US studies, and in single studies set in Finland, Israel, and Brazil. This is consistent with a wealth of other evidence on how co-payments reduce uptake of necessary care. Health insurance coverage was found to be associated with improved outcomes of hypertension care in US settings.
Limitations/risk of bias	- grey literature not searched;<br>- list of excluded studies not provided;<br>- preponderance of US studies limits the generalizability of the findings;<br>- formal quality assessment of included studies poorly described and discussed; only global ratings provided; unclear how any of the domains were operationalized and assessed.
Results - drug use (average effects)	n/a
Results - drug use (by sub-groups such as SES, chronically ill, elderly, ...)	Health insurance and lower cost-sharing were associated with hypertension treatment (defined as the use of at least one antihypertensive medication in an individual with known hypertension) and antihypertensive medication adherence.<br>*Magnitude, individuals with hypertension*: unclear<br>*Magnitude, individuals with hypertension vs. individuals without hypertension*: unclear
Results - health outcomes	n/a
Results - health outcomes (by sub-groups such as SES, chronically ill, elderly, ...)	Health insurance and lower cost-sharing were associated with hypertension awareness and hypertension control in individuals being treated for hypertension, or, alternatively, measured by the mean blood pressure amongst individuals with hypertension.<br>*Magnitude, individuals with hypertension:* unclear<br>*Magnitude, individuals with hypertension vs. individuals without hypertension:* unclear
Results - healthcare services utilization (i.e., non-pharmaceutical services)	n/a
Results - healthcare services utilization (i.e., non-pharmaceutical services) (by sub-groups such as SES, chronically ill, elderly, ...)	n/a

Pimentel, Lapane, Briesacher, 2013 [22]

Type of review/publication	- Narrative review.<br>- journal: Drugs Aging
Research question, overall	The impact of Medicare Part D on the long-term care context, specifically costs to long-term care residents, providers and payers; prescription drug coverage and utilization; and clinical and administrative outcomes.
Research question, specific to drug insurance/cost-sharing	The impact of Medicare Part D on prescription drug utilization and clinical outcomes.
Was an 'a priori' design provided?	No
Was there duplicate study selection and data extraction?	No. One reviewer independently completed the three stages (title, abstract, full-text) of the review and two reviewers determined whether articles were appropriate for inclusion.
Was a comprehensive literature search performed?	Yes. Search for peer-reviewed literature was supplemented with website search. Searched bibliographies of publications deemed eligible for inclusion.<br>- databases: PubMed, Cumulative Index to Nursing and Allied Health Literature (CINAHL), Health Business Fulltext Elite and Science Citation Index Expanded;<br>- languages: English only;<br>- year/month of last search: January 2013;<br>- grey literature included: yes;<br>- keyword/search strategy reported: yes
Search strategy, results	- Total number of studies included: 19<br>- Total number of studies, drugs/cost-sharing/insurance: 11<br>- Total number of Canadian studies: 0
Was a list of studies (included and excluded) provided?	No; list of excluded studies not provided.
Were the characteristics of the included studies provided?	Yes; authors/year/reference, study design, data source, time period, LTC setting (n), unit of observation (n), outcomes (costs to LTC residents, providers and payers; prescription drug coverage; prescription drug utilization; clinical; administrative).
Was the scientific quality of the included studies assessed and documented?	Yes; quality rating scale developed by Downs and Black (1998) to assess study quality. The modified checklist consists of 16 items with a maximum score of 14 points awarded for: study design; appropriate reporting of study objectives, methods and results; external validity and internal validity. Unclear which items were removed and which were kept. Some global ratings reported. Full assessment not provided. Unclear how any of the domains were operationalized and assessed.
Was the scientific quality of the included studies used appropriately in formulating conclusions?	To some extent. A few of the studies' limitations were discussed, but the quality assessment was not considered when formulating conclusions.
Were the methods used to combine the findings of studies appropriate?	n/a
Was the likelihood of publication bias assessed?	n/a

Funding, conflicts of interest reported?	Funding:<br>- review: yes; no specific funding received. Research scientist award acknowledged from the National Institute on Aging;<br>- included studies: no;<br>Conflict of interest:<br>- review: yes; no conflicts of interests reported;<br>- included studies: no.
Study's conclusion (as stated by the authors)	Empirical evidence of Medicare Part D's impact on long term care is sparse. The prescription drug benefit may require further modifications to more effectively provide for medication needs and improve health outcomes
Limitations/risk of bias	- no 'a priori' design;<br>- no duplicate study selection and data extraction;<br>- formal quality assessment of included studies poorly described and discussed; only global ratings provided; unclear how any of the domains were operationalized and assessed;<br>- list of excluded studies not provided.
Results - drug use (average effects)	n/a
Results - drug use (by sub-groups such as SES, chronically ill, elderly, ...)	Findings of prescription drug utilization were mixed. Prescription drug benefit was associated with decreased use of drugs that carry safety concerns, but overall drug utilization may have been unaffected. A shift in drug utilization within drug classes was seen (i.e., from non-covered to covered drugs and utilization of new drugs to treat side effects);<br>*Magnitude, seniors*: unclear<br>*Magnitude, seniors vs. non-seniors*: unclear
Results - health outcomes	n/a
Results - health outcomes (by sub-groups such as SES, chronically ill, elderly, ...)	Results were overall inconsistent. Clinician reports suggested a high incidence of adverse events (e.g., psychiatric hospital admissions, emergency department visits) immediately following medicare prescription drug plan and adverse effects of prescription drug substitutions for formulary-related reasons, however, some long-term care providers did not perceive adverse health effects of Part D among residents<br>*Magnitude, seniors:* unclear<br>*Magnitude, seniors vs. non-seniors:* unclear
Results - healthcare services utilization (i.e., non-pharmaceutical services)	n/a
Results - healthcare services utilization (i.e., non-pharmaceutical services) (by sub-groups such as SES, chronically ill, elderly, ...)	n/a

Kiil, Houlberg, 2014 [23]

Type of review/publication	- narrative review.<br>- journal: European Journal of Health Economics
Research question, overall	What are the behavioural effects of copayment within the health area across countries? What is the extent to which copayment reduces individual demand for services on which it is imposed, has adverse health effects, and give rise to distributional consequences.
Research question, specific to drug insurance/cost-sharing	What are the behavioural effects of copayment for prescription drugs across countries? What is the extent to which copayment for prescription drugs reduces individual demand for services on which it is imposed, has adverse health effects, and give rise to distributional consequences.
Was an 'a priori' design provided?	No
Was there duplicate study selection and data extraction?	Unclear
Was a comprehensive literature search performed?	No.<br>- databases: EconLit<br>- languages: English, Danish, Swedish;<br>- year/month of last search: December 2011;<br>- grey literature included: yes;<br>- keywords/search strategy reported: yes.
Search strategy, results	- Total number of studies included: 47<br>- Total number of studies, drugs/cost-sharing/insurance: 18<br>- Total number of Canadian studies: 9<br>• BC : Wang, Patrick, et al. 2010 ; Li, Guh, et al. 2007;<br>• ON : Zhong, 2007<br>• QB : Poirier, LeLorier,, et al. 1998; Tamblyn, Laprise, et al. 2001; Pilote, Beck, et al. 2002; Grootendorst, Levine, 2002; Blais, Couture, et al. 2003; Contoyannis, Hurley, et al. 2005.
Was a list of studies (included and excluded) provided?	No; list of excluded studies not provided.
Were the characteristics of the included studies provided?	Yes; references, country, analysis (focus, design, method), data (type, years, level), publication type.
Was the scientific quality of the included studies assessed and documented?	No
Was the scientific quality of the included studies used appropriately in formulating conclusions?	To some extent, quality of the included studies were not considered when making conclusions, but authors discuss general limitations of some studies when formulating conclusions.

Were the methods used to combine the findings of studies appropriate?	n/a
Was the likelihood of publication bias assessed?	n/a
Funding, conflicts of interest reported?	Funding:<br>- review: no;<br>- included studies: no;<br>Conflict of interest:<br>- review: no;<br>- included studies: no.
Study's conclusion (as stated by the authors)	Considering the demand effects, the majority of the reviewed studies found that copayment reduces the use of prescription medicine. The empirical evidence on whether copayment for some services, but not for others, causes substitution from the services that are subject to copayment to the 'free' services rather than lower total use is sparse and mixed.
Limitations/risk of bias	- no 'a priori' design;<br>- unclear duplicate study selection and data extraction;<br>- non-systematic search strategy;<br>- no formal quality assessment of included studies.
Results - drug use (average effects)	Overall, pharmaceutical copayments had negative effects on use of prescription medicine. The extent to which copayment affected the use of prescription medicine depended on the type of medicine as well as the patient population;<br>*Magnitude*: unclear
Results - drug use (by sub-groups such as SES, chronically ill, elderly, ...)	The majority of the reviewed studies found that copayment led to a larger reduction in the use of prescription medicine for vulnerable population groups than for the non-vulnerable general population;<br>*Magnitude, vulnerable population*: unclear<br>*Magnitude, vulnerable population vs. non-vulnerable general population*: unclear
Results - health outcomes	Overall, the effects of copayments on mortality was unclear. The health effects of copayment have only been analyzed empirically in a limited number of studies, of which half did not find any significant effects in the short-term.<br>Some studies observed a drop in the use of essential medicines following an increase in copayment which led to an increase in mortality, while increased drug compliance because of a drop in copayment reduced rate of mortality.<br>*Magnitude:* unclear
Results - health outcomes (by sub-groups such as SES, chronically ill, elderly, ...)	n/a
Results - healthcare services utilization (i.e., non-pharmaceutical services)	Overall, pharmaceutical copayments had positive effects on the substitution to other types of health care services (such as hospitalization, accident emergency departments, long-term care, general practise consultation);<br>*Magnitude:* unclear
Results - healthcare services utilization (i.e., non-pharmaceutical services) (by sub-groups such as SES, chronically ill, elderly, ...)	n/a

Mann, Barnieh, et al., 2014 [24]

Type of review/publication	- narrative review.<br>- journal: PLOS One
Research question, overall	What is the impact of drug insurance (vs. no drug insurance) and varying levels of patient cost-sharing (i.e. copayment, deductible, caps, and maximum out-of-pocket expenditure) on medication adherence, clinical and economic outcomes in patients with cardiovascular-related chronic disease?
Research question, specific to drug insurance/cost-sharing	Same.
Was an 'a priori' design provided?	No
Was there duplicate study selection and data extraction?	Yes
Was a comprehensive literature search performed?	Yes.<br>- databases: MEDLINE, EMBASE, CINAHL, Cochrane Controlled Trials Register, Current Controlled Trials;<br>- languages: English only;<br>- year/month of last search: March 2013;<br>- grey literature included: no;<br>- keywords/search strategy reported: yes.
Search strategy, results	Total number of studies included: 11<br>Total number of studies relevant to drug insurance/cost-sharing: 11<br>Total number of Canadian studies: 3<br>• BC: Schneeweiss, Patrick, et al. 2007-Circulation; Schneeweiss, Patrick, et al. 2007-Am J Manag Care;<br>• QC: Pilote, Beck, et al. 2002.
Was a list of studies (included and excluded) provided?	No; list of excluded studies not provided.
Were the characteristics of the included studies provided?	Yes, the following characteristics are reported in a figure: study design, study setting, intervention and comparator, number of patients, cost-sharing policy [illegible]
Was the scientific quality of the included studies assessed and documented?	To some extent; Cochrane risk of bias tool for RCT, and Cochrane EPOC taxonomy for non-RCTs, controlled before-after studies and interrupted time series designs were used to assess the quality of the studies. 7 components were rated as low, mid, high risk. No information was provided to justify ratings.
Was the scientific quality of the included studies used appropriately in formulating conclusions?	No. The scientific quality of the studies was not discussed in the context of discussing results and formulating conclusions.
Were the methods used to combine the findings of studies appropriate?	n/a
Was the likelihood of publication bias assessed?	n/a

Funding, conflicts of interest reported?	Funding:<br>- review: yes; Alberta Innovates - Health Solutions, Government of Canada, Government of Alberta;<br>- included studies: no;<br>Conflict of interest:<br>- review: yes; no conflicts of interests reported;<br>- included studies: no.
Study's conclusion (as stated by the authors)	Lowering cost-sharing in patients with chronic diseases may improve adherence, but the impact on clinical and economic outcomes is uncertain.
Limitations/risk of bias	- no 'a priori' design;<br>- list of excluded studies not provided;<br>- search limited to English;<br>- grey literature not searched;<br>- unclear how any of the quality criteria were operationalized and assessed;<br>- quality assessment not explicitly taken into account.
Results - drug use (average effects)	n/a
Results - drug use (by sub-groups such as SES, chronically ill, elderly, ...)	- individuals with cardiovascular-related chronic disease<br>The addition of drug insurance for those without previous drug insurance appear to have consistently increased adherence to medications. In general, studies evaluating drug insurance cost-sharing strategies had conflicting results with some studies showing significant differences in some outcomes while other studies demonstrated no discernible difference in outcomes. The use of deductibles (up to $350 per year) did not appear to have a significant impact on medication adherence.<br>The impact of a maximum out-of-pocket limits was uncertain;<br>*Magnitude, chronically ill*: unclear<br>*Magnitude, chronically ill vs. non-chronically ill*: unclear
Results - health outcomes	n/a
Results - health outcomes (by sub-groups such as SES, chronically ill, elderly, ...)	- individuals with cardiovascular-related chronic diseases<br>Results for clinical outcomes were scarce and mixed (only 2 studies were identified).<br>*Magnitude, individuals with cardiovascular-related chronic disease:* Unclear<br>*Magnitude, individuals with cardiovascular-related chronic diseases vs. individuals without cardiovascular-related chronic diseases:* unclear
Results - healthcare services utilization (i.e., non-pharmaceutical services)	n/a
Results - healthcare services utilization (i.e., non-pharmaceutical services) (by sub-groups such as SES, chronically ill, elderly, ...)	n/a

Kesselheim, Huybrechts et al., 2015 [25]

Type of review/publication	- Narrative review.<br>- Journal: American Journal of Public Health
Research question, overall	To determine how expansions or restrictions in prescription drug insurance have affected patients' health outcomes or their use of health care services.
Research question, specific to drug insurance/cost-sharing	Same.
Was an 'a priori' design provided?	No
Was there duplicate study selection and data extraction?	Yes
Was a comprehensive literature search performed?	Yes.<br>- databases: MEDLINE via Ovid, EMBASE, EconLit, and Business Source Complete;<br>- languages: English only;<br>- year/month of last search: May 2014;<br>- grey literature included: no<br>- keywords/search strategy reported: yes.
Search strategy, results	- Total number of studies included: 23<br>- Total number of studies, drugs/cost-sharing/insurance: 23<br>- Total number of Canadian studies: 0
Was a list of studies (included and excluded) provided?	No; list of excluded studies not provided.
Were the characteristics of the included studies provided?	Yes; study design, population sample, study years, participant details, exposure/intervention, main outcomes, findings, funding source.
Was the scientific quality of the included studies assessed and documented?	Yes; used guidelines outlined in Cochrane Handbook for Systematic Reviews of Interventions; 6 components: generation, allocation concealment, blinding, incomplete outcome data, selective outcome reporting, and other sources of bias.
Was the scientific quality of the included studies used appropriately in formulating conclusions?	Yes
Were the methods used to combine the findings of studies appropriate?	n/a
Was the likelihood of publication bias assessed?	n/a
Was the conflict of interest included?	Funding:<br>- review: yes; CVS Caremark;<br>- included studies: no;<br>Conflict of interest:<br>- review: yes; Agency for Healthcare Research and Quality, Robert Wood Johnson Foundation, National Institute of Mental Health<br>- included studies: no.

Study's conclusion (as stated by the authors)	Prescription drug insurance can have significant effects on both outcomes patient health status and health care service. Benefits were demonstrated in a variety of clinical circumstances, geographic regions, and temporal settings.
Limitations/risk of bias	- no 'a priori' design;<br>- search limited to English;<br>- grey literature not searched;<br>- surprisingly low number of studies identified;<br>- list of excluded studies not provided;<br>- only summary scores presented; unclear what led to low quality scores;<br>- 22 of 23 included studied were conducted in the United States which limits the generalizability of the findings.
Results - drug use (average effects)	– Prescription drug insurance coverage<br>Three studies examined the impact of drug insurance on patients' use of drugs and adherence by comparing cohorts of patients with and without coverage. Two of three studies found that those with insurance used more drugs;<br>*Magnitude*: unclear<br>– Extending drug insurance<br>*Magnitude:* One study examined the effects of extending drug coverage to patients on their drug use and found that the number of prescription fills increased non-significantly by 2 per patient per year;<br>– Drug insurance restriction<br>Six studies evaluated the effects of drug insurance restrictions on drug utilization and adherence. All studies found that drug insurance restrictions led to lower drug utilization and/or adherence.<br>*Magnitude*: unclear
Results - drug use (by sub-groups such as SES, chronically ill, elderly, ...)	n/a
Results - health outcomes	- Six studies evaluated the impact of drug insurance on patients' health by comparing cohorts of patients with and without coverage. Four of six studies found that those with insurance had better treatment adherence and/or health outcomes (self-reported health, mortality, functional disability, hospitalizations);<br>- Five studies examined the effects of extending drug coverage to patients on their health outcomes; findings were mixed;<br>- Five studies evaluated the effects of drug insurance restrictions on health outcomes. Four of five studies found that drug insurance restrictions led to worse treatment adherence and health outcomes (emergency department use, hospitalizations, health outcomes, rates of death)<br>*Magnitude:* unclear
Results - health outcomes (by sub-groups such as SES, chronically ill, elderly, ...)	n/a

Results - healthcare services utilization (i.e., non-pharmaceutical services)	Multiple studies found that limiting drug insurance was associated with an increase in the use of health services including emergency department use, hospitalizations, nursing home admissions, psychiatric hospitalizations, outpatient mental health visits, and emergency mental health services; other studies find that the expansion of drug insurance led to reductions in hospitalizations;<br>*Magnitude:* one study reported the effect of reaching the coverage limit in Part D: Emergency department use (relative risk [RR] 1.6; 95%CI 1.4, 1.8) and hospitalizations (RR 1.9; 95%CI 1.6, 2.1);<br>Another study reported positive associations between reaching the Part D coverage gap and worse outcomes among patients in psychiatric institutions with schizophrenia and bipolar disorder, including hospitalizations (schizophrenia: hazard ratio [HR] 1.32; 99.5%CI 1.06, 1.65; bipolar disorder: HR 1.45; 99.5%CI 1.16, 1.82). *********CAN YOU DOUBLE-CHECK THAT THE CIS ARE 99.5% AND NOT 95%. (the CIs are 99.5%, i went back and checked the original study that the review cited and they provide 99.5% CIs; also when reading this study I found that the second HR reported in the review for bipolar disorder is incorrect, it should be 1.29, (99.5%CI:1.02-1.64)> should we keep the one reported in the original study?********
Results - healthcare services utilization (i.e., non-pharmaceutical services) (by sub-groups such as SES, chronically ill, elderly, ...)	n/a

Luiza, Chavez et al., 2015 [26]

Type of review/publication	- Narrative review<br>- Report: The Cochrane Library
Research question, overall	To determine the effects of cap and co-payment policies on rational use of medicines, healthcare utilization, health outcomes and costs.
Research question, specific to drug insurance/cost-sharing	Same.
Was an 'a priori' design provided?	Yes.
Was there duplicate study selection and data extraction?	Yes.
Was a comprehensive literature search performed?	Yes.<br>- databases: Cochrane Library; MEDLINE; EMBASE; IPSA; EconLit; Worldwide Political Science Abstracts; PAIS International; INRUD Bibliography; WHOLIS, LILACS; Global Health Library; PubMed; BIREME; OpenGrey; JOLIS Library Network; OECD Library; World Bank e-Library; World Health Organization; World Bank Documents & Reports; International Clinical Trials Registry Platform (ICTRP); ClinicalTrials.gov;<br>- languages: no language restrictions;<br>- year/month of last search: January 2013;<br>- grey literature included: yes;<br>keyword/search strategy reported: no.
Search strategy, results	- Total number of studies included: 32<br>- Total number of studies included relevant to drug insurance/cost-sharing: 32<br>- Total number of Canadian studies: 9<br>• BC: Caetano, Raymond et al., 2006; Dormuth, Glynn et al., 2006; Dormuth, Maclure et al., 2008; Wang, Patrick, et al., 2008; Dormuth, Neumann et al, 2009;<br>• ON: Hux, Naylor, Fielding, 1997;<br>• QC: Poirier, LeLorier et al., 1998; Tamblyn, Laprise et al., 2001; Blais, Couture et al., 2003.
Was a list of studies (included and excluded) provided?	Yes.
Were the characteristics of the included studies provided?	Yes; interventions, outcomes, participants, methods.
Was the scientific quality of the included studies assessed and documented?	Yes.<br>- For RCTs, and Interrupted time series (ITS)/Repeated Measures (RM), assessed risk of bias using the Cochrane EPOC tool criteria that provides 9 standard domains for RCTs and 7 domains for ITS/RM. Summary ratings and justifications are provided for each study<br>- Controlled studies: 1. Random sequence generation; 2. Allocation concealment; 3. Baseline outcomes similarity; 4. Baseline characteristics similarity; 5. Incomplete outcome data assessment. 6. Blinding of outcome assessment; 7. Protection against contamination; 8. Selective outcome reporting; 9. Other risk of bias.<br>- Interrupted time series (ITS)/Repeated Measures (RM): 1. Intervention independent of other changes (protection against secular changes); 2. Shape of the intervention prespecified; 3. Intervention unlikely to affect data collection (protection against detection bias); 4. Blinding of outcome assessment; 5. Incomplete outcome data assessment; 6. Avoidance of selective outcome reporting. 7. Other risk of bias.<br>- Confidence in overall estimates graded using GRADE.

Was the scientific quality of the included studies used appropriately in formulating conclusions?	Yes.
Were the methods used to combine the findings of studies appropriate?	n/a
Was the likelihood of publication bias assessed?	n/a
Funding, conflicts of interest reported?	Funding:<br>- review: yes; Alliance for Health Policy and Systems Research<br>- included studies: yes<br>Conflict of interest:<br>- review: yes<br>- included studies: yes
Study's conclusion (as stated by the authors)	Overall, this review found that introducing or raising direct patient payments for medicines through caps, co-payments, co-insurance or combinations of these was found to reduce the use of both important and unimportant medicines across studies. However, impact was sometimes uncertain and varied from small to moderate relative reductions. Reductions were found among medicines for symptomatic and asymptomatic conditions. These included medicines that were important for treating chronic conditions.
Limitations/risk of bias	- restrictive inclusion criteria may limit the usefulness of the review.
Results - drug use (average effects)	Raising direct patient payments for medicines was found to reduce the use of both important and unimportant drugs. The impact was sometimes uncertain and varied from small to moderate relative reductions;<br>*Magnitude*: unclear
Results - drug use (by sub-groups such as SES, chronically ill, elderly, ...)	Not explicitly discussed.
Results - health outcomes	No studies were included that reported effects of cost-sharing on health outcomes.
Results - health outcomes (by sub-groups such as SES, chronically ill, elderly, ...)	n/a
Results - healthcare services utilization (i.e., non-pharmaceutical services)	The effects of pharmaceutical cost-sharing on emergency department use, hospitalization or use of outpatient care were uncertain;<br>*Magnitude:* unclear
Results - healthcare services utilization (i.e., non-pharmaceutical services) (by sub-groups such as SES, chronically ill, elderly, ...)	Not explicitly discussed.

Aziz, Hatah,e t al., 2016 [27]

Type of review/publication	- Narrative review (systematic)<br>- journal: Patient Prefer Adherence
Research question, overall	How do payment scheme affect patients' adherence to medications.
Research question, specific to drug insurance/cost-sharing	Same,
Was an 'a priori' design provided?	No
Was there duplicate study selection and data extraction?	No; initial screening was performed by one author, full-text review was conducted by three reviewers, conflicts were resolved through consensus.
Was a comprehensive literature search performed?	Yes.<br>- databases: Medline, ProQuest Medical Library, ScienceDirect<br>- languages: English only;<br>- year/month of last search: February 2015;<br>- grey literature included: no;<br>- keywords/search strategy reported: yes.
Search strategy, results	- Total number of studies included: 21<br>- Total number of studies, drugs/cost-sharing/insurance: 21<br>- Total number of Canadian studies: 2<br>• Canada: Law, Cheng, et al., 2012;<br>• ON: Zheng, Poulose, Fulford, et al., 2012
Was a list of studies (included and excluded) provided?	No; list of excluded studies not provided.
Were the characteristics of the included studies provided?	Yes; authors, study design, patient groups, number of patients, patients' diagnosis, method of payment, method of medication adherence, primary outcome, secondary outcome.
Was the scientific quality of the included studies assessed and documented?	Yes, the potential for bias was evaluated using the Downs and Black guidelines which analyzed 27 items based on the data quality, external and internal validity, and power of the studies.<br>Items 1–10: assessed whether the information provided was sufficient to allow the reader to make an unbiased assessment of the finding of the study; items 11–13: assessed external validity – which addressed the extent to which findings from the study could be generalized to the population from which the study subjects were derived; items 14–20: assessed potential bias – which addressed biases in the measurement of the intervention and the outcome; items 21–26: assessed confounding – which addressed bias in the selection of the study subjects; item 27: assessed the power of study – which attempted to assess whether the negative findings from a study could be due to chance.
Was the scientific quality of the included studies used appropriately in formulating conclusions?	No
Were the methods used to combine the findings of studies appropriate?	n/a
Was the likelihood of publication bias assessed?	n/a

Funding, conflicts of interest reported?	Funding:<br>- review: yes; Ministry of Higher Education, Malaysia;<br>- included studies: no;<br>Conflict of interest:<br>- review: yes; no conflicts of interests reported;<br>- included studies: no.
Study's conclusion (as stated by the authors)	High out-of-pocket expenditure and lack of or no prescription drug coverage were strongly associated with reduction of patients' adherence to medication. Although reduction in out-of-pocket medication expenditure may improve patients' adherence to medication, the nonadherence rate among patients who received medication at no cost was found to be high especially in non-severe disease. Factors that may influence the nonadherence among subsidized patients were unknown.
Limitations/risk of bias	- no 'a priori' design;<br>- no duplicate study selection;<br>- grey literature not searched;<br>- concerning low number of studies included;<br>- search limited to English;
Results - drug use (average effects)	Lower cost-sharing, higher prescription caps, subsidies, and insurance were associated with higher medication adherence;<br>*Magnitude*: unclear
Results - drug use (by sub-groups such as SES, chronically ill, elderly, ...)	n/a
Results - health outcomes	n/a
Results - health outcomes (by sub-groups such as SES, chronically ill, elderly, ...)	n/a
Results - healthcare services utilization (i.e., non-pharmaceutical services)	n/a
Results - healthcare services utilization (i.e., non-pharmaceutical services) (by sub-groups such as SES, chronically ill, elderly, ...)	n/a

Banerjee, Khandelwal, et al., 2016 [28]

Type of review/publication	- Narrative review.<br>- journal: Open Heart
Research question, overall	What are the barriers and facilitators to adherence to secondary cardiovascular disease (CVD) prevention medications at health system level.
Research question, specific to drug insurance/cost-sharing	Does drug insurance/cost-sharing act as barrier or facilitator to CVD medication adherence?
Was an 'a priori' design provided?	Yes
Was there duplicate study selection and data extraction?	Yes
Was a comprehensive literature search performed?	Yes.<br>- databases: MEDLINE, Embase, Cochrane Library, Psychinfo, Health Systems Evidence, Health Management Information Consortium (HMIC), LILACS, Africa-Wide Information and Google Scholar;<br>- languages: no restrictions;<br>- year/month of last search: October 2015;<br>- grey literature included: yes;<br>- keywords/search strategy reported: yes.
Search strategy, results	- Total number of studies included: 25<br>- Total number of studies, drugs/cost-sharing/insurance: 4<br>- Total number of Canadian studies: 0
Was a list of studies (included and excluded) provided?	No; list of excluded studies not provided.
Were the characteristics of the included studies provided?	Yes; authors, year, barriers/facilitators, setting, study design, sample size, study details, outcomes, relevant findings.
Was the scientific quality of the included studies assessed and documented?	Yes. For observational study designs, risk of bias was assessed using three domains: selection bias, information bias (differential misclassification and non-differential misclassification) and confounding. For RCTs, the Cochrane risk of bias tool was used (selection bias, performance bias, detection bias, attrition bias, reporting bias, other bias). Assessment not provided (only presence or absence of bias for each component provided along with generic total assessment [low, unclear, high]).
Was the scientific quality of the included studies used appropriately in formulating conclusions?	Yes
Were the methods used to combine the findings of studies appropriate?	n/a
Was the likelihood of publication bias assessed?	Yes. Publication bias was assessed by funnel plot analysis. Although meta-analysis was not undertaken, funnel plot asymmetry suggests possible publication bias.
Funding, conflicts of interest reported?	Funding:<br>- review: yes; World Heart Federation;<br>- included studies: no;<br>Conflict of interest:<br>- review: yes; no conflicts of interests reported;<br>- included studies: no.

Study's conclusion (as stated by the authors)	High-quality evidence on health system barriers and facilitators to adherence to secondary prevention medications for cardiovascular disease is lacking, especially for low-income settings. Full prescription coverage, reduced copayments, fixed-dose combination and counselling may be effective in improving adherence and are priorities for further research.
Limitations/risk of bias	- quality assessment: only summary scores presented; unclear what led to low quality scores;<br>- small number of relevant included studies (4) limits the usefulness and generalizability of the findings;<br>- list of excluded studies was not provided.
Results - drug use (average effects)	n/a
Results - drug use (by sub-groups such as SES, chronically ill, elderly, ...)	Reduced copayments and full prescription coverage were associated with increased adherence and persistence.<br>*Magnitude, individuals with cardiovascular diseases*: Two retrospective cohort studies investigated the impact of copayments on adherence. Among 4105 patients with acute myocardial infarction in Austria, those with waived copayments had higher persistence at 120 days for ACEI/ARB than those with copayments (OR 1.4, 95%CI 1.1 to 1.7), but β blocker (OR 1.1, 95%CI 0.9 to 1.4) or statin use (OR 1.1, 95%CI 0.9 to 1.3) did not significantly differ between these groups. The second US study of coronary heart disease patients found that compared with copayment <US$10, copayment ≥US$20 was associated with lower persistence at 1 year for statins (OR 0.42; 95%CI 0.36 to 0.49). A US-based prospective cohort study of 7955 myocardial infarction patients in 216 hospitals showed that non-persistence to secondary prevention medications was less likely with private insurance (OR 0.85, 95% CI 0.76 to 0.95) and prescription cost assistance (OR 0.63, 95%CI 0.54 to 0.75). A US-based RCT included 5855 individuals post-MI, randomized to full or usual prescription coverage. Full adherence was higher with full prescription coverage for all medication classes (OR 1.4, 1.2 to 1.7). Increased adherence to all three medications for the patient subgroup undergoing coronary artery bypass graft was found, post hoc (OR 1.7, 95% CI 1.04 to 2.7).<br>*Magnitude, individuals with cardiovascular diseases vs. individuals without cardiovascular diseases:* unclear
Results - health outcomes	n/a
Results - health outcomes (by sub-groups such as SES, chronically ill, elderly, ...)	n/a
Results - healthcare services utilization (i.e., non-pharmaceutical services)	n/a
Results - healthcare services utilization (i.e., non-pharmaceutical services) (by sub-groups such as SES, chronically ill, elderly, ...)	n/a

Doshi, Li, et al., 2016 [29]

Type of review/ publication	- systematic review<br>- The American Journal of Managed Care
Research question, overall	What is the impact of cost-sharing on utilization of speciality drugs indicated for rheumatoid arthritis, multiple sclerosis, and cancer, and on use of nondrug medical services, health outcomes and spending?
Research question, specific to drug insurance/cost-sharing	Same.
Was an 'a priori' design provided?	No, a priori design was not provided. Authors only included the inclusion/ exclusion criteria.
Was there duplicate study selection and [illegible]	All stages of the review were conducted independently, by 2 investigators but there is no reporting of how disagreement was resolved.
Was a comprehensive literature search performed?	Unclear.<br>- databases: Medline<br>- languages: English;<br>- year/month of last search: 2014;<br>- grey literature included: no;<br>- keywords/search strategy reported: yes.<br>- The search was also supplemented by reviewing the reference lists of selected articles.
Search strategy, results	Total number of included studies: 19<br>Total number of studies relevant to drug insurance/cost-sharing: 19<br>Total number of Canadian studies: 0
Was a list of studies (included and excluded) provided?	No, only included studies are provided.
Were the characteristics of the included studies provided?	Yes. Presented in appendix.
Was the scientific quality of the included studies assessed and documented?	No, the scientific quality of each included study was not assessed by the authors but general limitations are discussed
Was the scientific quality of the included studies used appropriately in formulating conclusions?	Yes, the quality and limitations of the included studies were included as part of the analysis and the interpretation of results in the discussion section.
Were the methods used to combine the findings of studies appropriate?	n/a
Was the likelihood of publication bias assessed?	n/a

Funding, conflicts of interest reported?	Funding:<br>- review: Pharmaceutical Research and Manufacturers of America (PhRMA); Pfizer;<br>- included studies: no<br>Conflict of interest:<br>- review: yes. Dr Doshi has served as a consultant or advisory board member for Alkermes Inc, Boehringer Ingelheim, Forest Laborato- ries, Ironwood Pharmaceuticals, Merck & Co Inc, and Shire; has received grants in the past from Amgen Inc, Pfizer Inc, Humana Inc, PhRMA, and the National Pharmaceutical Council; and has a spouse who owns stock in Merck & Co Inc and Pfizer Inc. Dr Pettit has received consulting fees from Alkermes Inc.<br>- included studies: no
Study's conclusion (as stated by the authors)	Higher cost-sharing was associated with reductions in utilization of speciality drugs for rheumatoid arthritis, MS and cancer. The evidence was not consistent and varied by speciality drug use outcome and by disease.
Limitations/risk of bias	- no 'a priori' design;<br>- non-systematic search strategy;<br>- grey literature not searched;<br>- list of excluded studies not provided;<br>- no formal quality assessment of included studies;
Results - drug use (average effects)	– Prescription abandonment (prescription submitted and approved by the insurer but not obtained by the patient): All studies (n=3) reported a strong association of higher cost-sharing with abandonment (vs initiation) of specialty drug prescriptions, for all indications examined.<br>*Magnitude, individuals using specialty drugs*: unclear<br>– Initiation (first time use of specialty drug within a study period): All studies (n=8) examining initiation in patients with rheumatoid arthritis and multiple sclerosis reported a negative association with higher cost sharing. Initiation of specialty drugs for cancer was largely reported to be insensitive to cost-sharing in the 3 studies examining this outcome.<br>*Magnitude, individuals using specialty drugs*: The demand elasticity ranged from -0.03 to -0.33 for patients with rheumatoid arthritis or multiple sclerosis<br>– Adherence: Evidence on relationship between cost-sharing and adherence was mixed. Majority of studies reported a statistically significant increase in discontinuation associated with increased cost-sharing.<br>*Magnitude, individuals using specialty drugs*: unclear<br>– Discontinuation/persistence (having a continuous gap of time between prescription fills): Six of the 7 studies reported a statistically significant increase in discontinuation (or decrease in persistence) associated with increased cost sharing for at least 1 of the indications examined.<br>*Magnitude, individuals using specialty drugs*: the magnitude of the effects appeared small.
Results - drug use (by sub-groups such as SES, chronically ill, elderly, ...)	n/a
Results - health outcomes	Research examining broader health outcomes was unvailable.
Results - health outcomes (by sub-groups such as SES, chronically ill, elderly, ...)	n/a

Results - healthcare services utilization (i.e., non-pharmaceutical services)	Studies have not examined effects on medical utilization.
Results - healthcare services utilization (i.e., non-pharmaceutical services) (by sub-groups such as SES, chronically ill, elderly, ...)	n/a

Powell, Saloner, Sabik, 2016 [30]

Type of review/publication	- Systematic review<br>- Journal: Medical Care Research and Review
Research question, overall	To synthesize the literature on the effects of cost-sharing, focusing on low-income populations in USA and to evaluate key evidence on four cost-sharing assumptions
Research question, specific to drug insurance/cost-sharing	To synthesize the literature on the effects of cost-sharing, focusing on low-income populations in USA, on health care utilization and spending
Was an 'a priori' design provided?	No.
Was there duplicate study selection and data extraction?	Unclear.
Was a comprehensive literature search performed?	Yes<br>- databases: PubMed/Medline. CINAHL, ISI Web of Science<br>- languages: English only<br>- year/month of last search: May 2014<br>- grey literature included: yes<br>- keywords/search strategy reported: yes
Search strategy, results	- Total number of studies included: Unclear<br>- Total number of studies, drugs/cost-sharing/insurance: Unclear<br>- Total number of Canadian studies: 0
Was a list of studies (included and excluded) provided?	No, only key references are given
Were the characteristics of the included studies provided?	Only for the key references (population, methods, major findings)
Was the scientific quality of the included studies assessed and documented?	When assessing quality of study design, the authors considered those that analyzed natural experiments (i.e., state government–initiated changes in cost sharing) to be higher quality. They also gave preference to studies with larger sample sizes (>1,000 subjects), and included representative data from multiple states (or at least multiple geographic areas of the same state).
Was the scientific quality of the included studies used appropriately in formulating conclusions?	No
Were the methods used to combine the findings of studies appropriate?	n/a
Was the likelihood of publication bias assessed?	n/a

Funding, conflicts of interest reported?	Funding:<br>- review: yes; Robert Wood Johnson Health and Society Scholars Program, National Institutes of Health co-funded by the National Cancer Institute and the Office of Behavioural and Social Sciences Research;<br>- included studies: no<br>Conflict of interest:<br>- review: yes; no conflicts of interest reported<br>- included studies: no
Study's conclusion (as stated by the authors)	Cost sharing has a deterrent effect on initiation of new treatments and medications, and in some cases can reduce utilization of ongoing treatments for chronic conditions, potentially leading to avoidable complications.
Limitations/risk of bias	- no 'a priori' design;<br>- search limited to English language studies<br>- no/unclear duplicate study selection and data extraction;<br>- list of included and excluded studies not provided;<br>- results not clearly synthesized<br>- US focus limits the generalizability of the findings
Results - drug use (average effects)	Increasing copayments resulted in decreased utilization of drugs and higher rates of non-adherence. However, the size of the effect varies across subgroups.<br><br>*magnitude:* unclear
Results - drug use (by sub-groups such as SES, chronically ill, elderly, ...)	In patients with high need for prescription drugs relatively, find that increased copayments results in decreased adherence. This was found in Medicaid patients with schizophrenia and privately insured adults with diabetes and congestive heart failure who were living in lowest median income areas.<br>m*agnitude, chronically ill:* unclear<br>m*agnitude, chronically ill vs non-chronically-ill:* unclear<br>m*agnitude, poor:* unclear<br>m*agnitude, poor vs non-poor:* unclear
Results - health outcomes	N/A
Results - health outcomes (by sub-groups such as SES, chronically ill, elderly, ...)	N/A
Results - healthcare services utilization (i.e., non-pharmaceutical services)	N/A
Results - healthcare services utilization (i.e., non-pharmaceutical services) (by sub-groups such as SES, chronically ill, elderly, ...)	Reduced use of prescription drugs from non-adherence has been linked to adverse consequences. A study of Medicaid beneficiaries with cancer found that after relatively small copayments were imposed ($0.50-$3.00) in Georgia in 2002, days supply of medication decreased and odds of an ED visit increased. Outside Medicaid, there is strong evidence from a natural experiment in Quebec where increased copayments for prescription drugs led to a spike in hospitalizations.<br><br>*magnitude, chronically ill:* unclear<br><br>m*agnitude, chronically ill vs non-chronically-ill:* unclear

Gourzoulidis, Kourlaba, et al., 2017 [31]

Type of review/publication	- Narrative review.<br>- journal: Health Policy
Research question, overall	To determine the association between copayment, medication adherence and outcomes in patients with heart failure and diabetes mellitus.
Research question, specific to drug insurance/cost-sharing	Same.
Was an 'a priori' design provided?	No
Was there duplicate study selection and data extraction?	Yes
Was a comprehensive literature search performed?	Unclear.<br>- databases: PubMed, Scopus and Cochrane databases;<br>- languages: English only;<br>- year/month of last search: not reported;<br>- grey literature included: no;<br>- keywords/search strategy reported: yes.
Search strategy, results	- Total number of studies included: 38<br>- Total number of studies, drugs/cost-sharing/insurance: 11<br>- Total number of Canadian studies: 0 (Patterson, Blalock, et al., 2011 wrongly listed as a Canadian study)
Was a list of studies (included and excluded) provided?	No; list of excluded studies not provided.
Were the characteristics of the included studies provided?	Yes; study type, country, type of diabetes, year of data collection, impact on outcomes (health, resource utilization, economic), adherence measure, change in adherence by copayment change.
Was the scientific quality of the included studies assessed and documented?	To some extent; the quality of included studies was evaluated using a modified version of Effective Public Health Practice project Quality Assessment Tool (EPHPP). The methodological dimensions were: allocation bias, study design, confounders, blinding, data collection methods, attrition bias, intervention integrity and statistics. These domains are rated on a three-point scale (strong, moderate, weak) according to predefined criteria and procedures recommended for tool use, and then given an overall rating. Unclear how any of these criteria were operationalized and assessed.
Was the scientific quality of the included studies used appropriately in formulating conclusions?	Yes
Were the methods used to combine the findings of studies appropriate?	n/a
Was the likelihood of publication bias assessed?	n/a
Funding, conflicts of interest reported?	Funding:<br>- review: yes; Novartis;<br>- included studies: no;<br>Conflict of interest:<br>- review: yes; Novartis, Servier, Orion Pharma;<br>- included studies: no.

Study's conclusion (as stated by the authors)	There is no strong evidence demonstrating a direct effect of copayment changes on health and economic outcomes in patients with diabetes mellitus and heart failure. However, there is moderate evidence indicating that copayment increases may result in lower medication adherence, which in turn may lead to poorer health outcomes and higher total healthcare expenses. Thus, it can be mentioned that copayments could have an indirect impact on outcomes.
Limitations/risk of bias	- no 'a priori' design;<br>- no duplicate study selection and data extraction;<br>- search limited to English;<br>- grey literature not searched;<br>- list of excluded studies not provided;<br>- unclear how any of the quality criteria were operationalized and assessed;<br>- arbitrary threshold used to categorize the quality of included studies;<br>- limited generalizability of the findings (10/11 studies used US data).
Results - drug use (average effects)	n/a
Results - drug use (by sub-groups such as SES, chronically ill, elderly, ...)	Seven of eight studies evaluating the relationship between drug copayment and medication adherence in diabetes mellitus population and one of three in heart failure population, found a statistically significant inverse association between increases in copayments and medication adherence;<br>*Magnitude, individuals with heart failure or diabetes mellitus*: unclear<br>*Magnitude, individuals with heart failure or diabetes mellitus vs. individuals without heart failure or diabetes mellitus*: unclear
Results - health outcomes	n/a
Results - health outcomes (by sub-groups such as SES, chronically ill, elderly, ...)	Only one included study examined the association between changes in copayments and health outcomes (higher copayments were associated with poorer glycemic control;<br>*Magnitude, individuals with heart failure or diabetes mellitus:* each $5 increase in patient drug cost share resulted in a 0.1 % point increase in glycosylated hemoglobinA(1c)).<br>*Magnitude, individuals with heart failure or diabetes mellitus vs. individuals without heart failure or diabetes mellitus:* unclear
Results - healthcare services utilization (i.e., non-pharmaceutical services)	n/a
Results - healthcare services utilization (i.e., non-pharmaceutical services) (by sub-groups such as SES, chronically ill, elderly, ...)	Studies showed no significant association between copayment change and emergency department visits, office visits, hospitalizations or laboratory/diagnostic tests among patients with diabetes mellitus. One study found that higher drug copayments were associated with an increase in emergency department visits among patients with heart failure;<br>*Magnitude, chronically ill:* unclear<br>*Magnitude, chronically ill vs. non-chronically ill:* unclear

Park, Martin, 2017 [32]

Type of review/publication	- Narrative review (systematic)<br>- Journal: Health Services Research
Research question, overall	To update a past systematic review on whether Medicare Part D changed drug utilization and out-of-pocket (OOP) costs overall and within subpopulations, and to identify evidence gaps.
Research question, specific to drug insurance/cost-sharing	Same.
Was an 'a priori' design provided?	No
Was there duplicate study selection and data extraction?	No, one reviewer (YP) did the extraction, with ongoing discussion with EGM about screening and extraction
Was a comprehensive literature search performed?	Yes<br>- databases: PubMed/MEDLINE, EconLit/EBSCO, Social Services Abstracts/ProQuest, PAIS International/ProQuest, Business Source Complete/EBSCO, PsycINFO, Scopus, Grey Literature Report by New York Academy of Medicine, the Kaiser Family Foundation, and the National Bureau of Economic Research.<br>- languages: English only;<br>- year/month of last search: Nov 2015<br>- grey literature included: yes.<br>- keywords/search strategy reported: yes.
Search strategy, results	- Total number of studies included: 65<br>- Total number of studies, drugs/cost-sharing/insurance: 62<br>- Total number of Canadian studies: 0
Was a list of studies (included and excluded) provided?	No; list of excluded studies not provided.
Were the characteristics of the included studies provided?	Yes; research question, study design (data years, geographic location, population, treatment group, comparison group, time period, data sources, number of observations, and data analysis method), drug utilization, and OOP cost outcomes, and limitations.
Was the scientific quality of the included studies assessed and documented?	Yes; the authors used a predefined criteria and a fixed coding guide to systematically assess the risk of bias in each study. As the Institute of Medicine did not provide a universal checklist to evaluate bias (Institute of Medicine 2011), the rubric included bias common to observational studies: comparability between intervention and control groups, attrition, data collection and quality, measurement error, miss- ing data, and reliability and validity of the outcome measures. This rubric was pretested on five articles, and each reviewer subsequently scored articles on each dimension and an overall score for the total risk of bias. Scores were compared between reviewers, with discrepancies resolved through consensus.
Was the scientific quality of the included studies used appropriately in formulating conclusions?	Yes
Were the methods used to combine the findings of studies appropriate?	n/a

Was the likelihood of publication bias assessed?	n/a
Was the conflict of interest included?	Funding:<br>- review: yes; no specific funding received;<br>- included studies: no;<br>Conflict of interest:<br>- review: yes; no conflicts of interests reported;<br>- included studies: no.
Study's conclusion (as stated by the authors)	Overall, Medicare Part D enrolees have increased drug utilization and decreased OOP costs, but coverage gaps limit the program's impact. Beneficiaries whose insurance becomes more generous after enrolment had disproportionately increased drug utilization and decreased OOP costs. Outcomes among dual-eligibles were mixed. There is strong evidence on how Medicare Part D and the donut hole coverage gap affect utilization and OOP costs, but weak evidence on how effects vary among dual-eligibles or across diseases. Findings suggest that the Affordable Care Act's provisions to expand coverage and reduce the donut hole should improve patient outcomes.
Limitations/risk of bias	- no 'a priori' design;<br>- search limited to English;<br>- list of excluded studies not provided;<br>- no duplicate study selection and data extraction;<br>- US focus limits the generalizability of the findings.
Results - drug use (average effects)	Studies consistently found that Medicare Part D increased drug utilization across numerous outcomes, including medication persistence, number of days with possession of at least 1 drug within a class, annual prescription fills per person, drug access, and cost-related behaviour changes such as medication cessation, applying to pharmaceutical assistance programs, and receiving free prescription samples. Similarly, Medicare Part D coverage gaps negatively impacted drug utilization. The coverage gap prompted some substitution of generic for brand-name drugs.<br>*Magnitude, seniors:* the strongest effect sizes were for medication use and increases were highest among beneficiaries receiving low-income subsidies.<br>*Magnitude, seniors vs. non-seniors*: unclear
Results - drug use (by sub-groups such as SES, chronically ill, elderly, ...)	*Chronically ill*<br>Although utilization increased for most disease-specific studies, some studies had mixed outcomes. Most studies found increased utilization of diabetes-related medications However, beneficiaries with cancer and depression did not have significantly different drug utilization.<br>*Magnitude, chronically ill: unclear*<br>*Magnitude, chronically ill vs. non-chronically ill: unclear*
Results - health outcomes	n/a
Results - health outcomes (by sub-groups such as SES, chronically ill, elderly, ...)	n/a
Results - healthcare services utilization (i.e., non-pharmaceutical services)	n/a
Results - healthcare services utilization (i.e., non-pharmaceutical services) (by sub-groups such as SES, chronically ill, elderly, ...)	n/a

Gupta, McColl et al., 2018 [33]

Type of review/publication	- Narrative review<br>- Journal: Patient Prefer Adherence
Research question, overall	The extent, determinants, and consequences of cost-related nonadherence to prescription medications in Canada.
Research question, specific to drug insurance/cost-sharing	Association between drug insurance/cost-sharing and cost-related nonadherence to prescription medications in Canada.
Was an 'a priori' design provided?	No
Was there duplicate study selection and data extraction?	Unclear; study selection, yes; data extraction, unclear.
Was a comprehensive literature search performed?	Yes.<br>- databases: Medline, CINAHL, ProQuest, ScienceDirect, Global Health, Google Scholar;<br>- languages: English only;<br>- year/month of last search: February 2018;<br>- grey literature included: no;<br>- keywords/search strategy reported: yes.
Search strategy, results	- Total number of studies included: 37<br>- Total number of studies, drugs/cost-sharing/insurance: 20<br>- Total number of Canadian studies: 20<br>• BC: Anis, guh, et al., 2005; Zheng, Poulose, et al., 2012;<br>• ON: Zhong, 2007; Kratzer, Cheng, et al., 2015; Ungar, Kozyrskyj et al., 2008; Allin, Hurley, 2009;<br>• QC: Tamblyn, Laprise, et al., 2001; Tamblyn, Eguale, et al., 2014; Wang, Li et al., 2015; Després, Forget et al., 2016;<br>• Canada: Millar, 1999; Kennedy, Morgan, 2006, 2009; Kemp, Roughead et al., 2010; Law, Cheng, et al., 2012; Campbell, King-Shier et al., 2014; Hennessy, Sanmartin et al., 2016; Lee, Morgan 2017; Law, Cheng et al., 2018.<br><br>Rotermann, Sanmartin et al., 2014 incorrectly listed as having examined insurance.
Was a list of studies (included and excluded) provided?	No; list of excluded studies not provided.
Were the characteristics of the included studies provided?	Yes; study, province/country, population characteristic, sample size, study design, focus of the study, outcome measurement.
Was the scientific quality of the included studies assessed and documented?	No formal quality assessment. Limitations of included studies generally discussed.
Was the scientific quality of the included studies used appropriately in formulating conclusions?	To some extent; the quality of individual studies was generally discussed and taken into account.
Were the methods used to combine the findings of studies appropriate?	n/a
Was the likelihood of publication bias assessed?	n/a

Was the conflict of interest included?	Funding:<br>- review: yes; Queen’s University, Canadian Institutes for Health Research;<br>- included studies: no;<br>Conflict of interest:<br>- review: yes; no conflicts of interests reported;<br>- included studies: no.
Study's conclusion (as stated by the authors)	Canadians who were young (between 18 and 64 years), without drug insurance, had lower income or precarious or irregular employment, and high out-of-pocket expenditure on drugs were most likely to face cost-related nonadherence to their prescriptions.
Limitations/risk of bias	- no 'a priori' design;<br>- no/unclear duplicate study selection and data extraction;<br>- search limited to English;<br>- grey literature not searched;<br>- list of excluded studies not provided;<br>- no formal quality assessment of included studies.
Results - drug use (average effects)	Having prescription drug insurance was significantly associated with having access to prescription medication without financial barriers. High drug costs (i.e., ?5% of annual household income or ?$20 a month out-of-pocket) was the major determinant of cost-related non-adherence;<br>*Magnitude*: unclear
Results - drug use (by sub-groups such as SES, chronically ill, elderly, ...)	Three studies including people with cardiovascular conditions found that those spending ?> 5% costs of medications out of their pocket were more likely to report cost-related non-adherence than those spending ?< 5%;<br>*Magnitude, chronically ill*: unclear<br>*Magnitude, chronically ill vs. non-chronically ill*: unclear
Results - health outcomes	Few studies reported that cost-sharing for drugs in the form of co-payments led patients to forego essential medications and a decline in health care status;<br>*Magnitude:* unclear
Results - health outcomes (by sub-groups such as SES, chronically ill, elderly, ...)	Few studies reported that cost-sharing for drugs in the form of co-payments led patients to forego essential medications and a decline in health care status, especially in the vulnerable population.
Results - healthcare services utilization (i.e., non-pharmaceutical services)	Evidence regarding the impact of cost-related non-adherence on individual health outcomes such as disease exacerbation, poor self-reported health, increase in symptoms leading to increasing hospitalizations, emergency department visits, or mortality was limited and mixed. Two studies found that relative to those with no drug insurance, the insured made more use of physician services;<br>*Magnitude:* unclear
Results - healthcare services utilization (i.e., non-pharmaceutical services) (by sub-groups such as SES, chronically ill, elderly, ...)	A study conducted with elderly and social assistance recipients in Québec found that the introduction of cost-sharing was associated with increased rates of emergency department visits. Another study found that among elderly patients with rheumatoid arthritis, higher cost-sharing was associated with more physician visits and among those were admitted to the hospital at least once, there were more admissions;<br>*Magnitude, poor/chronically ill:* unclear<br>*Magnitude, poor/chronically ill vs. non-poor/chronically ill:* unclear

Ofori-Asenso, Jakhu et al., 2018 [34]

Type of review/publication	- Meta-analysis<br>- Journal: Journals of Gerontology: Medical Sciences
Research question, overall	What factors are associated with non-adherence and/or discontinuation of statins among older persons (65+)?
Research question, specific to drug insurance/cost-sharing	What is the association between co-payment and non-adherence and/or discontinuation of statins among older persons (65+)?
Was an 'a priori' design provided?	Yes
Was there duplicate study selection and data extraction?	Yes
Was a comprehensive literature search performed?	Yes.<br>- databases: Medline; Embase; CINAHL; PsycINFO; National Health Service Economic and Evaluation Database (NHSEED); Database of Abstracts of Reviews of Effects (DARE); Cochrane Central Register of Controlled Trials;<br>- languages: English only;<br>- year/month of last search: December 2016;<br>- grey literature included: yes;<br>- keywords/search strategy reported: yes.
Search strategy, results	- Total number of studies included: 45<br>- Total number of studies, drugs/cost-sharing/insurance: 6<br>- Total number of Canadian studies: 0
Was a list of studies (included and excluded) provided?	No; list of excluded studies not provided.
Were the characteristics of the included studies provided?	Yes; country, sample size, % females, design, adherence definition, factors associated with non-adherence/discontinuation, quality grading.

Was the scientific quality of the included studies assessed and documented?	Yes; Observational studies were assessed using a set of questions from the the National Institute of Health (NIH) Quality Assessment Tool for Observational Cohort and Cross-Sectional Studies. Unclear which question was used:<br>1. Was the research question or objective in this paper clearly stated?<br>2. Was the study population clearly specified and defined?<br>3. Was the participation rate of eligible persons at least 50%?<br>4. Were all the subjects selected or recruited from the same or similar populations (including the same time period)? Were inclusion and exclusion criteria for being in the study prespecified and applied uniformly to all participants?<br>5. Was a sample size justification, power description, or variance and effect estimates provided?<br>6. For the analyses in this paper, were the exposure(s) of interest measured prior to the outcome(s) being measured?<br>7. Was the timeframe sufficient so that one could reasonably expect to see an association between exposure and outcome if it existed?<br>8. For exposures that can vary in amount or level, did the study examine different levels of the exposure as related to the outcome (e.g., categories of exposure, or exposure measured as continuous variable)?<br>9. Were the exposure measures (independent variables) clearly defined, valid, reliable, and implemented consistently across all study participants?<br>10.Was the exposure(s) assessed more than once over time?<br>11.Were the outcome measures (dependent variables) clearly defined, valid, reliable, and implemented consistently across all study participants?<br>12.Were the outcome assessors blinded to the exposure status of participants?<br>13.Was loss to follow-up after baseline 20% or less?<br>14.Were key potential confounding variables measured and adjusted statistically for their impact on the relationship between exposure(s) and outcome(s)?<br><br>Randomized clinical trials (RCTs) were assessed using the Joanna Briggs Institute's (JBI) critical appraisal checklist for RCTs.<br>1. Was true randomization used for assignment of participants to treatment groups?<br>2. Was allocation to treatment groups concealed?<br>3. Were treatment groups similar at the baseline?<br>4. Were participants blind to treatment assignment?<br>5. Were those delivering treatment blind to treatment assignment?<br>6. Were outcomes assessors blind to treatment assignment?<br>7. Were treatment groups treated identically other than the intervention of interest?<br>8. Was follow up complete and if not, were differences between groups in terms of their follow up adequately described and analyzed?<br>9. Were participants analyzed in the groups to which they were randomized?<br>10.Were outcomes measured in the same way for treatment groups?<br>11.Were outcomes measured in a reliable way?<br>12.Was appropriate statistical analysis used?<br>13.Was the trial design appropriate, and any deviations from the standard RCT design (individual randomization, parallel groups) accounted for in the conduct and analysis of the trial?<br><br>Studies that scored ≥70% of the applied assessment criteria were graded as high quality.
Was the scientific quality of the included studies used appropriately in formulating conclusions?	No
Were the methods used to combine the findings of studies appropriate?	Yes; random effect models with inverse variance weighting. The I^2 statistic was used to quantify statistical heterogeneity across studies. Subgroup analyses were used to explore sources of heterogeneity.
Was the likelihood of publication bias assessed?	No

Was the conflict of interest included?	Funding:<br>- review: Monash University, National Health and Medical Research Council;<br>- included studies: no;<br>Conflict of interest:<br>- review: yes; honoraria from Amgen Australia; AstraZeneca/Bristol-Myers Squibb Australia; Janssen-Cilag; Merck, Sharp, and Dohme (Australia); Novartis Australia; Novo Nordisk; Sanofi; Servier Laboratories; Takeda Australia for work unrelated to this study.<br>- included studies: no.
Study's conclusion (as stated by the authors)	The implementation of health system cost-sharing measures such as copayments will need to be carefully considered especially with respect to low-income individuals as well as those with higher need for medical care. Our findings indicate that these are significant barriers to adhering to and continuing necessary preventive therapies.
Limitations/risk of bias	- search limited to English;<br>- grey literature not searched;<br>- list of excluded studies not provided;<br>- quality assessment: only global ratings provided; unclear how any of the domains were operationalized and assessed;<br>- arbitrary threshold used to categorize the quality of included studies.
Results - drug use (average effects)	n/a
Results - drug use (by sub-groups such as SES, chronically ill, elderly, ...)	Higher copayment/cost (not necessarily drug cost-sharing) increased the likelihood of non-adherence and of discontinuation;<br>*Magnitude, seniors*: The association between higher copayment and non adherence and discontinuation was positive with OR=1.4 (95% CI: 1.3-1.5) and OR=1.6 (95% CI: 1.5-1.7), respectively. Further interpretation not provided.<br>*Magnitude, seniors vs. non-seniors*: unclear
Results - health outcomes	n/a
Results - health outcomes (by sub-groups such as SES, chronically ill, elderly, ...)	n/a
Results - healthcare services utilization (i.e., non-pharmaceutical services)	n/a
Results - healthcare services utilization (i.e., non-pharmaceutical services) (by sub-groups such as SES, chronically ill, elderly, ...)	n/a

Schneider, Gaedke et al., 2018 [35]

Type of review/publication	- Meta-analysis<br>- Journal: The International Journal of Clinical Practice
Research question, overall	The characteristics of pharmacotherapy associated with non-adherence to medication in the CVD population, focusing only on studies with self-reported measures.
Research question, specific to drug insurance/cost-sharing	Same
Was an 'a priori' design provided?	unclear; a predefined protocol is mentioned but not provided
Was there duplicate study selection and data extraction?	No; study selection, yes; data extraction, no.
Was a comprehensive literature search performed?	Yes.<br>- databases: PubMed, LILACS, Academic Search, CINAHL.<br>- languages: unclear<br>- year/month of last search: May 2016<br>- grey literature included: unclear<br>- keywords/search strategy reported: yes
Search strategy, results	- Total number of studies included: 31<br>- Total number of studies, drugs/cost-sharing/insurance: 17<br>- Total number of Canadian studies: 1
Was a list of studies (included and excluded) provided?	To some extent. A complete list and overview of the included studies were reported; the number of studies excluded was provided and the reason for exclusion was given, however, there was no way for the reader to trace the excluded studies.
Were the characteristics of the included studies provided?	Yes. An overview of the included studies was provided narratively and in tables, including authors, sample size, country, design, condition, measure, adherence conceptualization, and quality scores.
Was the scientific quality of the included studies assessed and documented?	Yes. Performed quality assessment using STROBE criteria and listed quality score (%) for each included study in tables; However unclear how individual domains were operationalized and assessed.<br>- quality assessment: only global ratings provided; unclear how any of the domains were operationalized and assessed;
Was the scientific quality of the included studies used appropriately in formulating conclusions?	No; limitations were generally discussed.
Were the methods used to combine the findings of studies appropriate?	Yes, meta-analyses were conducted, heterogeneity was taken into account and sensitivity analyses was performed. The ORs were pooled using a random effects model.
Was the likelihood of publication bias assessed?	Yes.
Funding, conflicts of interest reported?	Funding:<br>- review: Brazilian Federal Agency for Support and Evaluation of Graduated Education-CAPES.<br>- included studies: no<br>Conflict of interest:<br>- review: yes, none declared<br>- included studies: no

Study's conclusion (as stated by the authors)	The results of this review suggest that access to insurance or another program that assists with medication costs was a protection factor for nonadherence. On the other hand, a high frequency of dosing was a risk factor for nonadherence. Therefore, these characteristics of pharmacotherapy must be considered to improve medication adherence among CVD patients.
Limitations/risk of bias	- no 'a priori' design;<br>- list of excluded studies not provided;<br>- no duplicate study extraction<br>- unclear if grey literature was searched;<br>- formal quality assessment of included studies conducted using a reporting checklist, poorly described and discussed; only global scored provided; unclear how any of the domains were operationalized and assessed.
Results - drug use (average effects)	n/a
Results - drug use (by sub-groups such as SES, chronically ill, elderly, ...)	Insurance or programs that assisted with medication costs were a protection factor for non-adherence among individuals with chronic cardiovascular diseases;<br>*Magnitude, chronically ill:* among individuals with chronic cardiovascular diseases, insurance or program that assisted with medication cost was associated with a 24% decrease in the risk of non-adherence (OR 0.76; 95%CI 0.60, 0.95);<br>*Magnitude, chronically ill vs. non-chronically ill: unclear*
Results - health outcomes	n/a
Results - health outcomes (by sub-groups such as SES, chronically ill, elderly, ...)	n/a
Results - healthcare services utilization (i.e., non-pharmaceutical services)<br><br>(as stated by the authors)	n/a
Results - healthcare services utilization (i.e., non-pharmaceutical services) (by sub-groups such as SES, chronically ill, elderly, ...)	n/a

Cheen, Tan et al., 2018 [36]

Type of review/publication	- Meta-analysis<br>- Journal: International Journal of Clinical Practice
Research question, overall	To assess prevalence of primary medication non-adherence in six common chronic diseases (asthma and/ or chronic obstructive pulmonary disease, depression, diabetes mellitus, hyperlipidaemia, hypertension and osteoporosis), to identify and categorize factors associated with primary medication nonadherence; and to explore characteristics that contributed to heterogeneity between studies.
Research question, specific to drug insurance/cost-sharing	To assess whether drug insurance/cost-sharing is associated with primary medication non-adherence among individuals with six common chronic diseases.
Was an 'a priori' design provided?	No
Was there duplicate study selection and data extraction?	Yes
Was a comprehensive literature search performed?	Yes;<br>- databases: Medline; Embase; Cochrane Library; CINAHL; PsycINFO;<br>- languages: English only;<br>- year/month of last search: August 2018;<br>- grey literature included: no;<br>- keywords/search strategy reported: yes.
Search strategy, results	- Total number of studies included: 33<br>- Total number of studies, drugs/cost-sharing/insurance: 8<br>- Total number of Canadian studies: 0
Was a list of studies (included and excluded) provided?	No; list of excluded studies not provided.
Were the characteristics of the included studies provided?	Yes; first author/country; study design; setting; chronic disease included; sample size (n); mean/median age; study period (months); duration of pre-period (months); definition of primary medication non-adherence; method of calculating primary medication non-adherence prevalence; prevalence of primary medication non-adherence (%).
Was the scientific quality of the included studies assessed and documented?	Yes. The Cochrane risk of bias tool was used to assess clinical trials, the Newcastle-Ottawa Scale for assessing cohort studies and the National Heart, Lung and Blood Institute Quality Assessment Tool for assessing cross-sectional studies. Unclear how domains were operationalized and assessed for Newcastle-Ottawa scale and the National Heart, Lung and Blood Institute Quality Assessment Tool.
Was the scientific quality of the included studies used appropriately in formulating conclusions?	No
Were the methods used to combine the findings of studies appropriate?	Yes; random effects models were used to obtain pooled estimates. Heterogeneity not formally examined (e..g, I^2 not computed); subgroup analysis used to examine study heterogeneity.
Was the likelihood of publication bias assessed?	No

Was the conflict of interest included?	Funding:<br>- review: no;<br>- included studies: no;<br>Conflict of interest:<br>- review: no;<br>- included studies: no.
Study's conclusion (as stated by the authors)	Factors commonly associated with primary medication non-adherence include younger age, number of concurrent medications, practitioner specialty and higher co-payment.
Limitations/risk of bias	- no 'a priori' design;<br>- search limited to English;<br>- grey literature not searched;<br>- list of excluded studies not provided;<br>- formal quality assessment of included studies poorly described and discussed;<br>- results may not be generalizable to other chronic conditions not included;<br>- all included studies conducted in predominantly English-speaking populations;<br>- unable to obtain pooled estimates of the strength of association of each factor because of substantial heterogeneity in the analyses of factors associated with primary medication non-adherence.
Results - drug use (average effects)	n/a
Results - drug use (by sub-groups such as SES, chronically ill, elderly, ...)	On the whole, among individuals with chronic diseases (asthma, chronic obstructive pulmonary disease, depression, diabetes mellitus, hyperlipidaemia, hypertension and osteoporosis), higher co-payments were associated with primary medication non-adherence;<br>*Magnitude, chronically ill*: A high co-payment amount had the strongest association with primary medication non-adherence, with ORs ranging from 1.01 to 33 (compared to lower co-payments);<br>*Magnitude, chronically ill vs. non-chronically ill*: unclear
Results - health outcomes	n/a
Results - health outcomes (by sub-groups such as SES, chronically ill, elderly, ...)	n/a
Results - healthcare services utilization (i.e., non-pharmaceutical services)	n/a
Results - healthcare services utilization (i.e., non-pharmaceutical services) (by sub-groups such as SES, chronically ill, elderly, ...)	n/a

Kolasa, Kowalcyzk, 2017 [37]

Type of review/publication	- Narrative review.<br>- journal: Health Economics, Policy and Law
Research question, overall	What is the association between prescription drug cost-sharing and health care consumption and health outcomes.
Research question, specific to drug insurance/cost-sharing	Same.
Was an 'a priori' design provided?	Yes; unclear if protocol is publicly available.
Was there duplicate study selection and data extraction?	Yes
Was a comprehensive literature search performed?	Yes.<br>- databases: The Cochrane Library, PubMed, Embase;<br>- languages: English only;<br>- year/month of last search: February 2016;<br>- grey literature included: no;<br>- keywords/search strategy reported: yes.
Search strategy, results	- Total number of studies included: 18<br>- Total number of studies, drugs/cost-sharing/insurance: 18<br>- Total number of Canadian studies: 2<br>• BC: Li, Guh, et al., 2007; Wang, Patrick, et al., 2010.
Was a list of studies (included and excluded) provided?	No; list of excluded studies not provided.
Were the characteristics of the included studies provided?	Yes; study objective, number of respondents, cost-sharing scheme, diagnosis of study population, mean age, follow-up, design and settings.
Was the scientific quality of the included studies assessed and documented?	Yes; used a checklist, adapted from Gardner, Machin, Campbell (1986), for the assessment of the statistical content of medical studies. The adapted checklist comprised 2 domains* and 9 items:<br>*Design features*<br>1. Was the objective of the study sufficiently described?<br>2. Was an appropriate study design used to achieve the objective?<br>3. Was there a satisfactory statement given of source of subjects?<br>4. Was there a power-based assessment of adequacy of sample size?<br>*Analysis and presentation*<br>5. Was there a statement adequately describing or referencing all statistical procedures used?<br>6. Were the statistical analyzes used appropriate?<br>7. Was the presentation of statistical material satisfactory?<br>8. Were the confidence intervals given for the main results?<br>9. Was the conclusion drawn from the statistical analysis justified?<br><br>In cases where more than three out of nine quality criteria were not met, the given study was considered as of poor quality.<br><br>* Gardner et al.'s checklist has 4 domains and 12 items. The authors incorrectly stated that the domain 'conduct of the study' was used; the only item in this domain was not used.

Was the scientific quality of the included studies used appropriately in formulating conclusions?	To some extent; none of the included studies were assessed as 'poor quality.'
Were the methods used to combine the findings of studies appropriate?	n/a
Was the likelihood of publication bias assessed?	n/a
Funding, conflicts of interest reported?	Funding:<br>- review: yes; no specific funds were received;<br>- included studies: no;<br>Conflict of interest:<br>- review: yes; no conflicts of interests reported;<br>- included studies: no.
Study's conclusion (as stated by the authors)	Copayments for pharmaceuticals may limit the drug consumption of patients in the short term but create unexpected demand for healthcare services in the longer term. The findings suggest that the bigger the out-of-pocket payment burden the fewer prescriptions filled, but the higher demand for health care services, such as outpatient and emergency room lists, as well as hospitalization. This review tends to suggest that a decrease in the burden of out-of-pocket payments might also improve patient health outcomes.
Limitations/risk of bias	- search limited to English;<br>- grey literature not searched;<br>- list of excluded studies not provided;<br>- arbitrary threshold used to categorize the quality of included studies;<br>- unclear how any of the quality criteria were operationalized and assessed;<br>- arbitrary threshold used to categorize the quality of included studies;
Results - drug use (average effects)	n/a
Results - drug use (by sub-groups such as SES, chronically ill, elderly, ...)	n/a
Results - health outcomes	Association between drug cost-sharing and health outcomes was reported in seven studies, of which five found statistically significant results of an inverse relationship. Six studies studied a direct relationship while one studied an indirect relationship through adherence. Health outcomes included self-assessed health, major vascular events, cardiovascular-related mortality and all-cause mortality;<br>*Magnitude:* unclear
Results - health outcomes (by sub-groups such as SES, chronically ill, elderly, ...)	n/a
Results - healthcare services utilization (i.e., non-pharmaceutical services)	All 11 included studies found positive associations between increases in out-of-pocket expenses for drugs and the use of health care services (9 of 11 found associations that were statistically significant). Health care services included physician visits, hospitalization, and emergency room visits;<br>*Magnitude:* unclear

Results - healthcare services utilization (i.e., non-pharmaceutical services)<br>(by sub-groups such as SES, chronically ill, elderly, ...)	n/a

Mishuk, Fasina, Qian, 2019 [38]

Type of review/publication	- Narrative review<br>- Journal: Research in Social and Administrative Pharmacy
Research question, overall	Evaluate the impact of US federal and state generic drug policies on drug use, spending, and patient outcomes.
Research question, specific to drug insurance/cost-sharing	Evaluate the impact of four domains related to drug insurance/cost-sharing on drug use, and patient outcomes:<br>- Medicare/Medicaid prescription coverage cap policy;<br>- Medicare Part D;<br>- Cost-sharing;<br>- Affordable Care Act (ACA)
Was an 'a priori' design provided?	No
Was there duplicate study selection and data extraction?	Yes
Was a comprehensive literature search performed?	Yes<br>- databases: PubMed, Web of Science, PsycINFO, and Business Source Premier;<br>- languages: English only;<br>- year/month of last search: June 2017<br>- grey literature included: no;<br>- keywords/search strategy reported: yes.
Search strategy, results	- Total number of studies included: 34<br>- Total number of studies, drugs/cost-sharing/insurance: 24<br>- Total number of Canadian studies: 0
Was a list of studies (included and excluded) provided?	No; list of excluded studies not provided.
Were the characteristics of the included studies provided?	Yes; policy domains, study (year), study design, population, policy intervention, outcomes, results.
Was the scientific quality of the included studies assessed and documented?	Yes; the Effective Public Health Practice Project (EPHPP) tool was used to evaluate all included studies for methodological quality and risk of bias. The "Quality Assessment Tool for Quantitative Studies" lead to an overall methodological rating of strong, moderate or weak in eight domains: selection bias; study design; confounders; blinding; data collection methods; withdrawals and dropouts; intervention integrity; analysis.<br>Global rating: strong if no weak ratings, moderate if one weak rating, weak if two or more weak ratings. Only global ratings provided. Unclear how any of the domains were operationalized and assessed.
Was the scientific quality of the included studies used appropriately in formulating conclusions?	No
Were the methods used to combine the findings of studies appropriate?	n/a
Was the likelihood of publication bias assessed?	n/a

Was the conflict of interest included?	Funding:<br>- review: yes; no specific funding received;<br>- included studies: no;<br>Conflict of interest:<br>- review: yes; no conflicts of interests reported;<br>- included studies: no.
Study's conclusion (as stated by the authors)	Policies lowering cost-sharing (n = 7 studies) were associated with increased patient's medication use and adherence, but the impact varied by therapeutic classes. Existing evidence evaluating Medicare Part D (n = 12 studies) suggested decreased prescription spending for beneficiaries and Medicare. Only two studies that examined the impact of Affordable Care Act (ACA) were identified; one examined impact of the ACA on utilization or health and found that the ACA was associated with lower discontinuation of treatment.
Limitations/risk of bias	- no 'a priori' design;<br>- search limited to English;<br>- grey literature not searched;<br>- list of excluded studies not provided;<br>- results not clearly synthesized;<br>- quality assessment: only global ratings provided; unclear how any of the domains were operationalized and assessed;<br>- US focus limits the generalizability of the findings.
Results - drug use (average effects)	Seven studies found that policies lowering prescription cost-sharing were associated with increased patient's medication use and adherence, but the impact varied by therapeutic classes.<br>*Magnitude*: unclear
Results - drug use (by sub-groups such as SES, chronically ill, elderly, ...)	Existing evidence evaluating Medicare Part D suggested decreased prescription spending for beneficiaries and increased use of generics. Policies lowering cost-sharing were associated with increased patient's medication use and adherence, but the impact varied by therapeutic classes while government insurance plans with higher cost-sharing were associated with reduced generic utilization. Evidence suggests that lower cost-sharing increased generic drug use which further enhanced medication adherence.<br>*Magnitude, seniors*: unclear<br>*Magnitude, seniors vs. non-seniors*: unclear
Results - health outcomes	n/a
Results - health outcomes (by sub-groups such as SES, chronically ill, elderly, ...)	n/a
Results - healthcare services utilization (i.e., non-pharmaceutical services)	Results not clearly synthesized.
Results - healthcare services utilization (i.e., non-pharmaceutical services) (by sub-groups such as SES, chronically ill, elderly, ...)	Government insurance plans with high-cost sharing on generic drugs were associated with less use of health services among children;<br>*Magnitude, children:* unclear<br>*Magnitude, children vs. adults:* unclear

REFERENCES

1. Adams AS, Soumerai SB, Ross-Degnan D. The case for a medicare drug coverage benefit: a critical review of the empirical evidence. *Annu Rev Public Health* 2001; **22**: 49-61.

2. Harten C, Ballantyne P. The Impact of Cost-Sharing within Canadian Provincial Drug Benefit Programs: A Review. *Journal of Pharmaceutical Finance, Economics and Policy* 2004; **13**(1): 35-53.

3. Lexchin J, Grootendorst P. Effects of Prescription Drug User Fees on Drug and Health Services Use and On Health Status in Vulnerable Populations: a Systematic Review of the Evidence. *Int J Health Serv* 2004; **34**(1): 101-22.

4. Rice T, Matsuoka KY. The impact of cost-sharing on appropriate utilization and health status: a review of the literature on seniors. *Med Care Res Rev* 2004; **61**(4): 415-52.

5. Gibson TB, Ozminkowski RJ, Goetzel RZ. The effects of prescription drug cost sharing: a review of the evidence. *Am J Manag Care* 2005; **11**(11): 730-40.

6. Maio V, Pizzi L, Roumm AR, et al. Pharmacy Utilization and the Medicare Modernization Act. 2005.

7. Briesacher BA, Gurwitz JH, Soumerai SB. Patients at-risk for cost-related medication nonadherence: a review of the literature. *J Gen Intern Med* 2007; **22**(6): 864-71.

8. Gemmill MC, Costa-Font J, McGuire A. In search of a corrected prescription drug elasticity estimate: a meta-regression approach. *Health Econ* 2007; **16**(6): 627-43.

9. Goldman DP, Joyce GF, Zheng Y. Prescription drug cost sharing: associations with medication and medical utilization and spending and health. *JAMA* 2007; **298**(1): 61-9.

10. Gemmill MC, Thomson S, Mossialos E. What impact do prescription drug charges have on efficiency and equity? Evidence from high-income countries. *Int J Equity Health* 2008; **7**: 12.

11. Remler DK, Greene J. Cost-sharing: a blunt instrument. *Annu Rev Public Health* 2009; **30**: 293-311.

12. Green CJ, Maclure M, Fortin PM, Ramsay CR, Aaserud M, Bardal S. Pharmaceutical policies: effects of restrictions on reimbursement. *The Cochrane database of systematic reviews* 2010; (8): CD008654.

13. Holst J. Patient cost sharing: Reforms without evidence. Theoretical considerations and empirical findings from industrialized countries, WZB Discussion Paper, No. SP I 2010-303. Berlin: Wissenschaftszentrum Berlin für Sozialforschung (WZB), 2010.

14. Polinski JM, Kilabuk E, Schneeweiss S, Brennan T, Shrank WH. Changes in drug use and out-of-pocket costs associated with Medicare Part D implementation: a systematic review. *J Am Geriatr Soc* 2010; **58**(9): 1764-79.

15. Polinski JM, Donohue JM, Kilabuk E, Shrank WH. Medicare Part D's effect on the under- and overuse of medications: a systematic review. *J Am Geriatr Soc* 2011; **59**(10): 1922-33.

16. Swartz K. Cost-sharing: Effects on spending and outcomes. The Synthesis Project. Research Synthesis Report No. 20. Princeton, NJ: Robert Wood Johnson Foundation, 2010.

17. Baicker K, Goldman D. Patient Cost-Sharing and Healthcare Spending Growth. *J Econ Perspect* 2011; **25**(2): 47-68.

18. Eaddy MT, Cook CL, O'Day K, Burch SP, Cantrell CR. How patient cost-sharing trends affect adherence and outcomes: a literature review. *P T* 2012; **37**(1): 45-55.

19. Lemstra M, Blackburn D, Crawley A, Fung R. Proportion and risk indicators of nonadherence to statin therapy: a meta-analysis. *Can J Cardiol* 2012; **28**(5): 574-80.

20. Sinnott S-J, Buckley C, O'Riordan D, Bradley C, Whelton H. The effect of copayments for prescriptions on adherence to prescription medicines in publicly insured populations; a systematic review and meta-analysis. *PLoS One* 2013; **8**(5): e64914.

21. Maimaris W, Paty J, Perel P, et al. The influence of health systems on hypertension awareness, treatment, and control: a systematic literature review. *PLoS Med* 2013; **10**(7): e1001490.

22. Pimentel CB, Lapane KL, Briesacher BA. Medicare part D and long-term care: a systematic review of quantitative and qualitative evidence. *Drugs Aging* 2013; **30**(9): 701-20.

23. Kiil A, Houlberg K. How does copayment for health care services affect demand, health and redistribution? A systematic review of the empirical evidence from 1990 to 2011. *The European journal of health economics : HEPAC : health economics in prevention and care* 2014; **15**(8): 813-28.

24. Mann BS, Barnieh L, Tang K, et al. Association between drug insurance cost sharing strategies and outcomes in patients with chronic diseases: a systematic review. *PLoS One* 2014; **9**(3): e89168.

25. Kesselheim AS, Huybrechts KF, Choudhry NK, et al. Prescription Drug Insurance Coverage and Patient Health Outcomes: A Systematic Review. *Am J Public Health* 2015; **105**(2): E17-E30.

26. Luiza VL, Chaves LA, Silva RM, et al. Pharmaceutical policies: effects of cap and co-payment on rational use of medicines. *The Cochrane database of systematic reviews* 2015; **5**: CD007017.

27. Aziz H, Hatah E, Makmor Bakry M, Islahudin F. How payment scheme affects patients' adherence to medications? A systematic review. *Patient preference and adherence* 2016; **10**: 837-50.

28. Banerjee A, Khandelwal S, Nambiar L, et al. Health system barriers and facilitators to medication adherence for the secondary prevention of cardiovascular disease: a systematic review. *Open heart* 2016; **3**(2): e000438.

29. Doshi JA, Li P, Ladage VP, Pettit AR, Taylor EA. Impact of cost sharing on specialty drug utilization and outcomes: a review of the evidence and future directions. *Am J Manag Care* 2016; **22**(3): 188-97.

30. Powell V, Saloner B, Sabik LM. Cost Sharing in Medicaid: Assumptions, Evidence, and Future Directions. *Med Care Res Rev* 2016; **73**(4): 383-409.

31. Gourzoulidis G, Kourlaba G, Stafylas P, Giamouzis G, Parissis J, Maniadakis N. Association between copayment, medication adherence and outcomes in the management of patients with diabetes and heart failure. *Health Policy* 2017; **121**(4): 363-77.

32. Park YJ, Martin EG. Medicare Part D's Effects on Drug Utilization and Out-of-Pocket Costs: A Systematic Review. *Health Serv Res* 2017; **52**(5): 1685-728.

33. Gupta S, McColl MA, Guilcher SJ, Smith K. Cost-related nonadherence to prescription medications in Canada: a scoping review. *Patient preference and adherence* 2018; **12**: 1699-715.

34. Ofori-Asenso R, Jakhu A, Curtis AJ, et al. A Systematic Review and Meta-analysis of the Factors Associated With Nonadherence and Discontinuation of Statins Among People Aged ≥65 Years. *J Gerontol A Biol Sci Med Sci* 2018; **73**(6): 798-805.

35. Schneider APH, Gaedke MA, Garcez A, Barcellos NT, Paniz VMV. Effect of characteristics of pharmacotherapy on non-adherence in chronic cardiovascular disease: A systematic review and meta-analysis of observational studies. *Int J Clin Pract* 2018; **72**(1).

36. Cheen MHH, Tan YZ, Oh LF, Wee HL, Thumboo J. Prevalence of and factors associated with primary medication non-adherence in chronic disease: A systematic review and meta-analysis. *Int J Clin Pract* 2019; **73**(6): e13350.

37. Kolasa K, Kowalczyk M. The effects of payments for pharmaceuticals: a systematic literature review. *Health Economics, Policy and Law* 2019; **14**(3): 337-54.

38. Mishuk AU, Fasina I, Qian J. Impact of U.S. federal and state generic drug policies on drug use, spending, and patient outcomes - A systematic review. *Res Social Adm Pharm* 2019.

Appendix D. Excluded studies

– Not a review

Morgan S, Daw JR, Law MR. Are income-based public drug benefit programs fit for an aging population?. IRPP 2014.

Tamblyn RM. Prescription drug coverage: An essential service or a fringe benefit?. CMAJ 2005; 173(11): 1343-4.

Anis AH, Guh DP, Lacaille D, et al. When patients have to pay a share of drug costs: effects on frequency of physician visits, hospital admissions and filling of prescriptions. CMAJ 2005; 173(11): 1335-40.

Gaynor M, Li J, Vogt WB. Is drug coverage a free lunch? Cross-price elasticities and the design of prescription drug benefits. NBER, 2006.

Gibson TB, McLaughlin CG, Smith DG. Generic utilization and cost-sharing for prescription drugs. Adv Health Econ Health Serv Res 2010; 22: 195-219.

Grootendorst P, Palfrey D, Willison D, et al. A review of the comprehensiveness of provincial drug coverage for Canadian seniors. Can J Aging 2003; 22(1): 33-44.

Jia L, Meng Q, Yuan B, et al. Effects of drug cost sharing policy on the drug use, financial risks and moral hazard for the health insurance beneficiaries. Value Health 2014; 17(7): A795.

Njie GJ, Mukhtar Q, Finnie RK, et al. Effectiveness of reducing out-of-pocket costs for medications to treat hypertension and hyperlipidemia: a community guide systematic review. J Am Soc Hypertens 2014; 8(4): e124-5.

Shenolikar R, Burch S, Gilmore A, et al. PHP39 Sensitivity of medication use and outcomes to formulary controls in the elderly: a review of the literature. Value Health 2010; 13(3): A88.

Sinnott SJ, Sinnott CM, Whelton H. The effect of reduced or removed copayments for prescription medicines on adherence - a systematic review. Value Health 2013; 16(7): A461.

Liu J. Balancing therapeutic safety and efficacy to improve clinical and economic outcomes in schizophrenia: a managed care perspective. Am J Manag Care 2014; 20(8 Suppl): S174-83.

Fairman KA, Curtiss FR. How do seniors respond to 100% cost-sharing for prescription drugs? Quality of the evidence underlying opinions about the Medicare Part D coverage gap. J Manag Care Med 2011; 17(5): 382-92.

Tamblyn R. The impact of pharmacotherapy policy: a case study. Can J Clin Pharmacol 2001; 8: 39A-44A.

Powe NR. Prescription drugs in Medicare and the ESRD program. Semin Nephrol 2000; 20(6): 535-42.

Dafny L, Ody C, Schmitt M. When discounts raise costs: the effect of copay coupons on generic utilization. Am Econ J Econ Policy 2017; 9(2): 91-123.

Alpert A. The anticipatory effects of Medicare Part D on drug utilization. J Health Econ 2016; 49: 28-45.

Ghosh A, Simon K, Sommers BD. The effect of state Medicaid expansions on prescription drug use: evidence from the Affordable Care Act. NBER, 2017.

Fout BT, Gilleskie DB. Does health insurance encourage or crowd out beneficial nonmedical care? A dynamic analysis of insurance, health inputs, and health production. Am J Health Econ 2015; 1(2): 125-64.

Wladysiuk M, Araszkiewicz A, Godman B, et al. Influence of patient co-payments on atypical antipsychotic choice in Poland. Appl Health Econ Health Policy 2011; 9(2): 101-10.

Skipper N. On utilization and stockpiling of prescription drugs when co-payments Increase: heterogeneity across types of drugs. Aarhus University School of Economics Working Paper, 2010.

Poisal JA. Medicaid drugs. Health Care Financ Rev 2004; 25(3): 1-4.

Morgan SG, Gagnon MA, Charbonneau M, et al. Evaluating the effects of Quebec's private–public drug insurance system. CMAJ 2017; 189(40): E1259-63.

Gagnon MA. The role and impact of cost-sharing mechanisms for prescription drug coverage. CMAJ 2017; 189(19): E680-1.

– No data on effects of cost-sharing and/or prescription drug insurance

Alsabbagh MW, Lemstra M, Eurich D, et al. Socioeconomic status and nonadherence to antihypertensive drugs: a systematic review and meta-analysis. Value Health 2014; 17(2): 288-96.

Babu BV, Babu GR. Coverage of, and compliance with, mass drug administration under the programme to eliminate lymphatic filariasis in India: a systematic review. Trans R Soc Trop Med Hyg 2014; 108(9): 538-49.

Hadley J. Sicker and poorer—the consequences of being uninsured: a review of the research on the relationship between health insurance, medical care use, health, work, and income. Med Care Res Rev 2003; 60(2 Suppl): 3S-75S.

Buchmueller TC, Grumbach K, Kronick R, et al. The effect of health insurance on medical care utilization and implications for insurance expansion: a review of the literature. Med Care Res Rev 2005; 62(1): 3-30.

Liu S, Chollet D. Price and income elasticity of the demand for health insurance and health care services: a critical review of the literature. Mathematica Policy Research, 2006.

Freeman JD, Kadiyala S, Bell JF, et al. The causal effect of health insurance on utilization and outcomes in adults: a systematic review of US studies. Med Care 2008; 46(10): 1023-32.

Crockett AB. Use of prescription drugs: rising or declining?. Nurs Clin North Am 2005; 40(1): 33-49.

Levy H, Meltzer D. What do we really know about whether health insurance affects health. Health policy and the uninsured, 2004: 179-204.

Ogbechie OA, Hsu J. Systematic review of benefit designs with differential cost sharing for prescription drugs. Am J Manag Care 2015; 21(5): e338-48.

Spaan E, Mathijssen J, Tromp N, et al. The impact of health insurance in Africa and Asia: a systematic review. Bull World Health Organ 2012; 90(9): 685-92.

Gruber J. The role of consumer copayments for health care: lessons from the RAND health insurance experiment and beyond. Henry J. Kaiser Family Foundation, 2016.

Gleason PP, Gunderson BW, Gericke KR. Are incentive-based formularies inversely associated with drug utilization in managed care?. Ann Pharmacother 2005; 39(2): 339-45.

Happe LE, Clark D, Holliday E, et al. A systematic literature review assessing the directional impact of managed care formulary restrictions on medication adherence, clinical outcomes, economic outcomes, and health care resource utilization. J Manag Care Spec Pharm 2014; 20(7): 677-84.

Laba TL, Bleasel J, Brien JA, et al. Strategies to improve adherence to medications for cardiovascular diseases in socioeconomically disadvantaged populations: a systematic review. Int J Cardiol 2013; 167(6): 2430-40.

Lee JL, Fischer MA, Shrank WH, et al. A systematic review of reference pricing: implications for US prescription drug spending. Am J Manag Care 2012; 18(11): e429-37.

Maddox TM, Ho PM. Medication adherence and the patient with coronary artery disease: challenges for the practitioner. Curr Opin Cardiol 2009; 24(5): 468-72.

Moe-Byrne T, Chambers D, Harden M, et al. Behaviour change interventions to promote prescribing of generic drugs: a rapid evidence synthesis and systematic review. BMJ Open 2014; 4(5): e004623.

Munshi KD, Shih YC, Brown LM, et al. Disparity implications of the Medicare medication therapy management eligibility criteria: a literature review. Expert Rev Pharmacoecon Outcomes Res 2013; 13(2): 201-16.

Page TF, Woodward RS. Cost–effectiveness of Medicare's coverage of immunosuppression medications for kidney transplant recipients. Expert Rev Pharmacoecon Outcomes Res 2009; 9(5): 435-44.

Pasma A, van't Spijker A, van Busschbach J, et al. What will determine adherence to pharmaceutical treatment for rheumatoid arthritis? a systematic review. Arthritis Rheumatol 2012; 64.

Piette JD, Heisler M, Horne R, et al. A conceptually based approach to understanding chronically ill patients' responses to medication cost pressures. Soc Sci Med 2006; 62(4): 846-57.

Pinto SL, Gangan N, Gangal N, et al. Tools used to improve medication adherence: a systematic review. Value Health 2013; 16(3): A42-3.

Pont L, Jansen K, Schaufel MA, et al. Drug utilization and medication costs at the end of life. Expert Rev Pharmacoecon Outcomes Res 2016; 16(2): 237-43.

Puig-Junoy J, Moreno-Torres I. Impact of pharmaceutical prior authorisation policies : a systematic review of the literature. Pharmacoeconomics 2007; 25(8): 637-48.

Roy S, Madhavan SS. Making a case for employing a societal perspective in the evaluation of Medicaid prescription drug interventions. Pharmacoeconomics 2008; 26(4): 281-96.

Thorpe CT, Lassila HC, O'Neil CK, et al. Reconsideration of key articles regarding medication related problems in older adults from 2011. Am J Geriatr Pharmacother 2012; 10(1): 2-13.

Ungar WJ, Ariely R. Health insurance, access to prescription medicines and health outcomes in children. Expert Rev Pharmacoecon Outcomes Res 2005; 5(2): 215-25.

Viswanathan M, Golin CE, Jones CD, et al. Interventions to improve adherence to self-administered medications for chronic diseases in the United States: a systematic review. Ann Intern Med 2012; 157(11): 785-95.

Wolfe D, Carrieri MP, Shepard D. Treatment and care for injecting drug users with HIV infection: a review of barriers and ways forward. Lancet 2010; 376(9738): 355-66.

Wood E, Kerr T, Tyndall MW, et al. A review of barriers and facilitators of HIV treatment among injection drug users. AIDS 2008; 22(11): 1247-56.

Ai AL, Carretta H, Beitsch LM, Watson L, et al. Medication therapy management programs: promises and pitfalls. J Manag Care Spec Pharm 2014; 20(12): 1162-82.

Babar ZU, Kan SW, Scahill S. Interventions promoting the acceptance and uptake of generic medicines: a narrative review of the literature. Health policy 2014; 117(3): 285-96.

Regenstein M, Andres E. Reducing hospital readmissions among medicaid patients: a review of the literature. Qual Manag Health Care 2014; 23(1): 20-42.

Keyhani S, Falk R, Howell EA, et al. Overuse and systems of care: a systematic review. Med Car 2013; 51(6): 503-8.

Menzin J, Caon C, Nichols C, et al. Narrative review of the literature on adherence to disease-modifying therapies among patients with multiple sclerosis. J Manag Care Spec Pharm 2013; 19(1 Suppl): S24-40.

Dylst P, Vulto A, Simoens S. The impact of reference-pricing systems in Europe: a literature review and case studies. Expert Rev Pharmacoecon Outcomes Res 2011; 11(6): 729-37.

Althaus F, Paroz S, Hugli O, et al. Effectiveness of interventions targeting frequent users of emergency departments: a systematic review. Ann Emerg Med 2011; 58(1): 41-52.

Ovsag K, Hydery S, Mousa SA. Preferred drug lists: potential impact on healthcare economics. Vasc Health Risk Manag 2008; 4(2): 403-13.

Sturm H, Austvoll-Dahlgren A, Aaserud M, et al. Pharmaceutical policies: effects of financial incentives for prescribers. Cochrane Database Syst Rev 2007; (3): CD006731.

Pedersen KM, Christiansen T, Bech M. The Danish health care system: evolution-not revolution-in a decentralized system. Health Econ 2005; 14(S1): S41-57.

O'Neill C, Hughes CM, Jamison J, et al. Cost of pharmacological care of the elderly: implications for healthcare resources. Drugs Aging 2003; 20(4): 253-61.

Lexchin J. Effects of restrictive formularies in the ambulatory care setting. Am J Manag Care 2002; 8(1): 69-76.

Ratanawijitrasin S, Soumerai SB, Weerasuriya K. Do national medicinal drug policies and essential drug programs improve drug use?: a review of experiences in developing countries. Soc Sci Med 2001; 53(7): 831-44.

Chaix-Couturier C, Durand-Zaleski I, Jolly D, et al. Effects of financial incentives on medical practice: results from a systematic review of the literature and methodological issues. Int J Qual Health Care 2000; 12(2): 133-42.

Dolovich LR, Holbrook AM, Woodruff M. The impact of reference pricing of cardiovascular drugs on health care costs and health outcomes: evidence from British Columbia--volume III: ACE and CCB literature review. McMaster University, 2002.

Aaserud M, Austvoll-Dahlgren A, Kösters JP, et al. Pharmaceutical policies: effects of reference pricing, other pricing, and purchasing policies. Cochrane Database Syst Rev 2006; (2): CD005979.

Hanlon JT, Lindblad CI, Gray SL. Can clinical pharmacy services have a positive impact on drug-related problems and health outcomes in community-based older adults?. Am J Geriatr Pharmacother 2004; 2(1): 3-13.

Lapane KL, Hughes CM. Optimising drug utilisation in long term care. Pharmacoeconomics 2002; 20(3): 143-52.

Lindström E, Bingefors K. Patient compliance with drug therapy in schizophrenia. Pharmacoeconomics 2000; 18(2): 105-24.

Voyer P, Cohen D, Lauzon S, et al. Factors associated with psychotropic drug use among community-dwelling older persons: a review of empirical studies. BMC Nurs 2004; 3(1): 1-3.

Williams A, Manias E, Walker R. Interventions to improve medication adherence in people with multiple chronic conditions: a systematic review. J Adv Nurs 2008; 63(2): 132-43.

No outcome measures relating to drug use, health service utilization and/or health outcome

Azores KF. Catastrophic drug coverage in Canada. Healthy Dialogue 2013; 2(1): 1-9.

Grootendorst P. Beneficiary cost sharing under Canadian provincial prescription drug benefit programs: history and assessment. Can J Clin Pharmacol 2002; 9(2): 79-99.

Atherly A. Supplemental insurance: Medicare's accidental stepchild. Med Care Res Rev 2001; 58(2): 131-61.

Lau DT, Briesacher BA, Touchette DR, et al. Medicare Part D and quality of prescription medication use in older adults. Drugs Aging 2011; 28(10): 797-807.

Palumbo FB, Simoni-Wastila L, Lavallee DC, et al. Access to pharmaceuticals in the post-Medicare Part D era. J Pharm Health Serv Res 2010; 1(1): 9-14.

Reissman D. An Independent Strategy for Managing Drug Utilization. Drug Benefit Trends 2006; 18(3): 180.

Solberg LI. Impact of Insurance Coverage on the Use an Effects of Smoking Cessation Medications. Disease Management Health Outcomes 2005; 13(3): 151-8.

Triki N, Pliskin JS, Greenberg D. Can linking co-payment for drugs to evidence on treatment value improve health outcomes and contain healthcare costs?. Harefuah 2010; 149(8): 524-8.

Virgo KS, Burkhardt EA, Cokkinides VE, et al. Impact of health care reform legislation on uninsured and medicaid-insured cancer patients. Cancer J 2010; 16(6): 577-83.

Balfour DC 3rd, Evans S, Januska J, et al. Medicare Part D-a roundtable discussion of current issues and trends. J Manag Care Pharm 2009; 15(1 Suppl): 3-9.

Owens G, Emons MF, Christian-Herman J, et al. Current trends in pharmacy benefit designs: a threat to disease management in chronic complex diseases. Dis Manag 2007; 10(2): 74-82.

Harris KM, Thomas C. Naltrexone and pharmacy benefit management. J Addict Dis 2004; 23(4): 11-29.

Laupacis A. Hard decisions about fundamental values. Healthc Pap 2004; 4(3): 60-6.

Olson BM. Approaches to pharmacy benefit management and the impact of consumer cost sharing. Clin Ther 2003; 25(1): 250-72.

Cohen J, Cairns C, Paquette C, et al. Comparing patient access to pharmaceuticals in the UK and US. Appl Health Econ Health Policy 2006; 5(3): 177-87.

Kelton CM, Rebelein RP, Heaton PC, et al. Differences in the cost of antidepressants across state Medicaid programs. J Ment Health Policy Econ 2008; 11(1): 33-47.

Ortun V, Puig-Junoy J, Callejón M. Drug innovation, prices and health. UPF Economics and Business Working Paper No. 807, 2005.

Ess SM, Schneeweiss S, Szucs TD. European healthcare policies for controlling drug expenditure. Pharmacoeconomics 2003; 21(2): 89-103.

– No data on effects of cost-sharing and/or prescription drug insurance and no outcome measures relating to drug use, health service utilization and/or health outcome

Call KT, Blewett LA. Revisiting crowd-out update. The Synthesis project. Research Synthesis Report 2007; (12 Suppl 1): 329-349.

Kiil A. What characterises the privately insured in universal health care systems? a review of the empirical evidence. Health Policy 2012; 106(1): 60-75.

Saloner B, Hochhalter S, Sabik L. Medicaid and CHIP premiums and access to care: a systematic review. Pediatrics, 2016; 137(3): e20152440.

Bitler MP, Zavodny M. Medicaid: a review of the literature. NBER, 2014.

Coile CC. Economic determinants of workers' retirement decisions. J Econ Surv 2015; 29(4): 830-53.

Limpa-Amara S, Merrill A, Rosenbach ML. SCHIP at 10: a Synthesis of the evidence on Ssbstitution of SCHIP for other coverage. Mathematica Policy Research, 2007.

Fendrick AM, Martin JJ, Weiss AE. Value-based insurance design: more health at any price. Health Serv Res 2012; 47(1pt2): 404-13.

Ekman B. Community-based health insurance in low-income countries: a systematic review of the evidence. Health Policy Plan 2004; 19(5): 249-70.

Thomson S, Schang L, Chernew ME. Value-based cost sharing in the United States and elsewhere can increase patients' use of high-value goods and services. Health Aff 2013; 32(4): 704-12.

Marchildon G. Canada: health system review. Health Syst Transit 2013; 15(1): 1-179.

Hurley J, Johnson M. A review of evidence regarding parallel systems of public and private finance. CHEPA McMaster University, 2014.

Hofmarcher MM, Quentin W. Austria: health system review. Health Syst Transit 2013; 15(7): 1-292.

Hyry HI, Cox TM, Roos JC. Saving orphan drug legislations: misconceptions and clarifications. Expert Rev Pharmacoecon Outcomes Res 2016; 16(1): 111-7

Janzen RW, Ludwig WD. Off-label therapy: current problems from the perspective of the Pharmaceutical Commission of the German Medical Profession.

Z Rheumatol 2012; 71(2):108.

Knapp M, Kanavos P, King D, et al. Economic issues in access to medications: schizophrenia treatment in England. Int J Law Psychiatry 2005; 28(5): 514-31.

Lloyd KB, Berger BA. Talking with patients about generic medications. US Pharm 2007; 32(6): 26.

Lopes Gde L Jr, de Souza JA, Barrios C. Access to cancer medications in low- and middle-income countries. Nat Rev Clin Oncol 2013; 10(6): 314-22.

Maclure M, Nakagawa RS, Carleton BC. Applying research to the policy cycle: implementing and evaluating evidence-based drug policies in British Columbia. Japanese Pharmacology and Therapeutics 2003; 31(8): 697-717.

Mathers BM, Degenhardt L, Ali H, et al. HIV prevention, treatment, and care services for people who inject drugs: a systematic review of global, regional, and national coverage. Lancet 2010; 375(9719): 1014-28.

Moran M. A breakthrough in R&D for neglected diseases: new ways to get the drugs we need. PLoS Med 2005; 2(9): e302.

Mossialos E, Walley T, Rudisill C. Provider incentives and prescribing behavior in Europe. Expert Rev Pharmacoecon Outcomes Res 2005; 5(1): 81-93.

Olveda DU, McManus DP, Ross AG. Mass drug administration and the global control of schistosomiasis: successes, limitations and clinical outcomes. Curr Opin Infect Dis 2016; 29(6): 595-608.

Pauwels K, Huys I, Casteels M, et al. Market access of cancer drugs in European countries: improving resource allocation. Target Oncol 2014; 9(2): 95-110.

Perry AE, Woodhouse R, Neilson M, et al. Are non-pharmacological interventions effective in reducing drug use and criminality? a systematic and meta-analytical review with an economic appraisal of these interventions. Int J Environ Res Public Health 2016; 13(10): 966

Roll K, Stargardt T, Schreyögg J. Authorization and reimbursement of orphan drugs in an international comparison (Zulassung Und Erstattung Von Orphan Drugs Im Internationalen Vergleich). Gesundheitswesen 2011; 73(8-9): 504-14.

Rollet P, Lemoine A, Dunoyer M. Sustainable rare diseases business and drug access: no time for misconceptions. Orphanet J Rare Dis 2013; 8: 109.

Sambamoorthi U, Akincigil A, Wei W, et al. National trends in out-of-pocket prescription drug spending among elderly medicare beneficiaries. Expert Rev Pharmacoecon Outcomes Res 2005; 5(3): 297-315.

Atre S. Addressing policy needs for prevention and control of type 2 diabetes in India. Perspect Public Health 2015; 135(5): 257-63.

Wirtz VJ, Kaplan WA, Kwan GF, et al. Access to medications for cardiovascular diseases in low- and middle-income countries. Circulation 2016 May; 133(21): 2076-85.

Lassi ZS, Musavi NB, Maliqi B, et al. Systematic review on human resources for health interventions to improve maternal health outcomes: evidence from low- and middle-income countries. Hum Resour Health 2016; 14: 10.

Menon D, Clark D, Stafinski T. Reimbursement of drugs for rare diseases through the public healthcare system in Canada: where are we now? Healthc Policy 2015; 11(1): 15-32.

Barua S, Greenwald R, Grebely J, et al. Restrictions for Medicaid reimbursement of Sofosbuvir for the treatment of Hepatitis C Virus infection in the United States. Ann Intern Med 2015; 163(3): 215-23.

Winkler MF, Smith CE. Clinical, social, and economic impacts of home parenteral nutrition dependence in short bowel syndrome. JPEN J Parenter Enteral Nutr 2014; 38(1 Suppl): 32S-37S.

Antonanzas F. The impact of the economic downturn on healthcare in Spain: consequences and alternatives. Expert Rev Pharmacoecon Outcomes Res 2013; 13(4): 433-9.

Hellander I. The deepening crisis in U.S. health care: a review of data. Int J Health Serv 2011; 41(3): 575-86.

Sanghvi TG, Harvey PW, Wainwright E. Maternal iron-folic acid supplementation programs: evidence of impact and implementation. Food Nutr Bull 2010; 31(2 Suppl): S100-7

Stuart B, Briesacher B. Medication decisions--right and wrong. Med Care Res Rev 2002; 59(2): 123-45.

Bowen A, Palasanthiran P, Sohn AH. Global challenges in the development and delivery of paediatric antiretrovirals. Drug Discov Today 2008; 13(11-12): 530-5.

Cherubini A, Corsonello A, Lattanzio F. Underprescription of beneficial medicines in older people: causes, consequences and prevention. Drugs Aging 2012; 29(6): 463-75.

Brady M. The National Drug Strategy and Indigenous Australians: missed opportunities and future challenges. Drug Alcohol Rev 2012; 31(6): 747-53.

Sagan A, Panteli D, Borkowski W, et al. Poland: Health system review. WHO 2011.

Sterling S, Weisner C, Hinman A, et al. Access to treatment for adolescents with substance use and co-occurring disorders: challenges and opportunities. J Am Acad Child Adolesc Psychiatry 2010; 49(7): 637-46.

Cruess A, Maberley D, Wong D, et al. The treatment of wet AMD in Canada: access to therapy (policy review). Can J Ophthalmol 2009; 44(5): 548-56.

Jacobsen R, Liubarskiene Z, Møldrup C, et al. Barriers to cancer pain management: a review of empirical research. Medicina (Kaunas) 2009; 45(6): 427-33.

Whitty CJ, Chandler C, Ansah E, et al. Deployment of ACT antimalarials for treatment of malaria: challenges and opportunities. Malar J 2008; 7(1): 1-7.

Size M, Soyannwo OA, Justins DM. Pain management in developing countries. Anaesthesia 2007; 62: 38-43.

Crystal S, Akincigil A, Bilder S, et al. Studying prescription drug use and outcomes with medicaid claims data: strengths, limitations, and strategies. Med Care 2007; 45(10 Supl 2): S58-65.

Sultan A, Thuan JF, Avignon A. Primary prevention of cardiovascular events and type 2 diabetes: should we prioritize our interventions? Diabetes Metab 2006; 32(6): 559-67.

Lewin Group. Medication therapy management services: a critical review. J Am Pharm Assoc 2005; 45(5): 580-7.

Honsinger RW. Complying with federal guidelines: Medicare Prescription Drug, Improvement, and Modernization Act of 2003: amendments to Social Security Act. Allergy Asthma Proc 2005; 26(1): 29-34.

Chequer P, Cuchí P, Mazin R, et al. Access to antiretroviral treatment in Latin American countries and the Caribbean. AIDS 2002; 16: S50-7.

Carroll NV. How effectively do managed care organizations influence prescribing and dispensing decisions? Am J Manag Care 2002; 8(12): 1041-54.

Rice N, Smith PC. Capitation and risk adjustment in health care financing: an international progress report. Milbank Q 2001; 79(1): 81-113.

McLellan AT, Lewis DC, O'Brien CP, et al. Drug dependence, a chronic medical illness: implications for treatment, insurance, and outcomes evaluation. JAMA 2000; 284(13): 1689-95.

Manga P. Economic case for the integration of chiropractic services into the health care system. J Manipulative Physiol Ther 2000; 23(2): 118-22.

Goldsmith L, Hurley JE, Hutchison BG. Economic evaluation across the four faces of prevention: a Canadian perspective. McMaster University Centre for Health Economics and Policy Analysis, 2004.

Blanchard JC, Haywood YC, Scott C. Racial and ethnic disparities in health: an emergency medicine perspective. Acad Emerg Med 2003; 10(11): 1289-93.

Chauhan D, Mason A. Factors affecting the uptake of new medicines in secondary care - a literature review. J Clin Pharm Ther 2008; 33(4): 339-48.

Chisholm M. Increasing medication access to transplant recipients. Clin Transplant 2004; 18(1): 39-48.

Diaby V, Laurier C, Lachaine J. A proposed framework for formulary listing in low-income countries. Pharmaceut Med 2011; 25(2): 71-82.

Flugsrud-Breckenridge MR, Gevirtz C, Paul D, et al. Medications of abuse in pain management. Curr Opin Anaesthesiol 2007; 20(4): 319-24.

Gulbins H, Vogel B, Reichenspurner H. Gender effects on health care costs in cardiovascular medicine-a black box? Thorac Cardiovasc Surg. 2013; 61(1): 74-8.

Hess GP, Fonseca E, Scott R, et al. Pharmacogenomic and pharmacogenetic-guided therapy as a tool in precision medicine: current state and factors impacting acceptance by stakeholders. Genet Res (Camb) 2015; 97: e13.

Ma YT, Palmer DH. Impact of restricting access to high-cost medications for hepatocellular carcinoma. Expert Rev Pharmacoecon Outcomes Res 2012; 12(4): 465-73.

Mennito SH, Clark JK. Transition medicine: a review of current theory and practice. South Med J 2010; 103(4): 339-42.

Santaguida PL, Gross A, Busse J, et al. Complementary and alternative medicine in back pain utilization report. Evid Rep Technol Assess (Full Rep) 2009; (177): 1-221.

Denburg AE, Ungar WJ, Greenberg M. Public drug policy for children in Canada. CMAJ 2017; 189(30): E990-E994.

– Not a review and no outcome measures on drug use, health services use, and/or health outcome

Smart M, Stabile M. Tax credits, insurance, and the use of medical care. Can J Econ 2005; 38(2): 345-65.

Dhalla IA, Guyatt GH, Stabile M, et al. Broadening the base of publicly funded health care. CMAJ 2011; 183(5): E296-305.

Canadian Institute for Health Information. Prescribed drug spending in canada: a focus on public drug programs. Canadian Institute for Health Information, 2012.

Law MR, Kratzer J, Dhalla IA. The increasing inefficiency of private health insurance in Canada. CMAJ 2014; 186(12): E470-4.

Paris V, Docteur É. Pharmaceutical pricing and reimbursement policies in Canada. OECD Health Working Papers No. 24, 2007

Gruber J, McKnight R. Why did employee health insurance contributions rise?. J Health Econ 2003; 22(6): 1085-104.

Mossialos E, Thomson S, World Health Organization. Voluntary health insurance in the European Union. Copenhagen: WHO Regional Office for Europe, 2004.

Thomson S, Mossialos E. Private health insurance in the European Union. European Commission, 2009: 320-3.

Grignon, Michel. Access and health insurance. 2014: 13-18.

Cohen JP. PBMs and a Medicare prescription drug benefit. Food Drug Law J 2000; 55(3): 311-20.

Gruber J. The impacts of the Affordable Care Act: how reasonable are the projections?. NBER, 2011.

Innovation U. Excellent healthcare for Canada. Report of the advisory panel on healthcare Innovation. Ministry of Health, Canada, 2015: 5.

Stabile M. Private financing outside the publicly funded system. M. Lu et E. Jonsson (dir. pub.), Financing Health Care: New Ideas for a Changing Society, 2008.

Jeske K, Kitao S. US tax policy and health insurance demand: can a regressive policy improve welfare?. J Monet Econ 2009; 56(2): 210-21.

Jaspersen JG, Richter A. The wealth effects of premium subsidies on moral hazard in insurance markets. Eur Econ Rev 2015; 77: 139-53.

García JL, Mardones HV, Escobar L, et al. The medicines situation in Chile: a critical appraisal from the academy. Pharmaceuticals Policy and Law 2014; 16(3, 4): 339-48.

Morgan SG, Martin D, Gagnon MA, et al. Pharmacare 2020: the future of drug coverage in Canada, 2016; 29.

Hågå A, Sverre JM. Pricing and reimbursement of pharmaceuticals in Norway. Eur J Health Econ 2002; 3(3): 215-20.

Hogerzeil HV. The concept of essential medicines: lessons for rich countries. BMJ 2004; 329(7475): 1169-72.

Huskamp HA, Keating NL. The new medicare drug benefit: formularies and their potential effects on access to medications. J Gen Intern Med 2005; 20(7): 662-5.

Hyde R, Dobrovolny D. Orphan drug pricing and payer management in the United States: are we approaching the tipping point?. Am Health Drug Benefits 2010; 3(1): 15-23.

Iglehart JK. The new Medicare prescription-drug benefit--a pure power play. N Engl J Med 2004; 350(8): 826-33.

U. Kohler. Prescription of lymphological remedies - security in relation to insurance companies and medical associations. Lymphologie in Forschung Und Praxis, 2007.

Landtblom AM, Ertzgaard P. The condition--not the drug--is decisive when it comes to subsidizing. Increased objectivity in the debate on subsidized "quality of life drugs" is required. Lakartidningen 2000; 97(21): 2612-4.

Larmour I, Thomson WB, Tsui MK, et al. Introduction of pharmaceutical benefits scheme reforms at three Victorian public health services. Journal of Pharmacy Practice and Research 2003; 33(3): 204-7.

Mao W, Tang S, Chen W. Does perverse economic incentive lead to the irrational uses of medicines? Expert Rev Pharmacoecon Outcomes Res 2013; 13(6): 693-6.

Maynard A, Cookson R. Money or your life? The health-wealth trade-off in pharmaceutical regulation. J Health Serv Res Policy 2001; 6(3): 186-9.

Miller JE. Restricting access to medications hurts patients, their families, and their communities. Drug Benefit Trends 2003; 15: 30-5.

Montaner JS, Wood E, Kerr T, et al. Expanded highly active antiretroviral therapy coverage among HIV-positive drug users to improve individual and public health outcomes. J Acquir Immune Defic Syndr 2010; 55: S5-9.

Morgan S, Bassett K, Mintzes B. Outcomes-based drug coverage in British Columbia. Health Aff (Millwood) 2004; 23(3): 269-76.

Murray R. Prescribing issues for Aboriginal people. Aust Prescr 2003; 26: 106-9.

Nash DB. Benefit Based Co-Pays: Fewer Tears. P and T, 2005.

Russi A, Serena M, Palozzo AC. Is the price of cancer drugs related to the cost of develo-pment and production or to the economic value of their clincal efficacy?. Recenti Prog Med 2016; 107(4): 181-5.

Ruxin J, Paluzzi JE, Wilson PA, et al. Emerging consensus in HIV/AIDS, malaria, tuberculosis, and access to essential medicines. Lancet 2005; 365(9459): 618-21

Schafheutle E. Is limiting rather than abolishing prescription charges the answer?. Pharmaceutical Journal 2006; 276(7394): 388-9.

Schulman KA, Balu S, Reed SD. Specialty pharmaceuticals for hyperlipidemia--impact on insurance premiums. N Engl J Med 2015; 373(17): 1591-3.

Schwieterman P. Navigating financial assistance options for patients receiving specialty medications. Am J Health Syst Pharm 2015; 72(24): 2190-5.

Siegel J, O'neal B. Code N: multidisciplinary approach to proactive drug diversion prevention. Hospital Pharmacy 2007; 42(3): 244-8.

Sketris I, Bowles S, Manuel R. Canadian public policies and practices related to drug prices, utilizatioin and expenditures. Journal of Pharmaceutical Finance, Economics and Policy 2004; 12(1): 23-54.

Wechsler J. Pharmaceutical pricing and healthcare access: Lead policy agenda. Pharm Technol 2004; 28(3): 26-34.

Vedanthan R, Kamano JH, Bloomfield GS, et al. Engaging the entire care cascade in western Kenya: a model to achieve the cardiovascular disease secondary prevention roadmap goals. Glob Heart 2015; 10(4): 313-7.

Simen-Kapeu A, Seale AC, Wall S, et al. Treatment of neonatal infections: a multi-country analysis of health system bottlenecks and potential solutions. BMC Pregnancy Childbirth 2015; 15 (Suppl 2): S6.

de Ridder L, Waterman M, Turner D, et al. Use of biosimilars in paediatric inflammatory bowel disease: a position statement of the ESPGHAN Paediatric IBD Porto Group. J Pediatr Gastroenterol Nutr 2015; 61(4): 503-8.

Ferré F, de Belvis AG, Valerio L, et al. Italy: health system review. WHO, 2014.

Agyepong IA, Aryeetey GC, Nonvignon J, et al. Advancing the application of systems thinking in health: provider payment and service supply behaviour and incentives in the Ghana National Health Insurance Scheme--a systems approach. Health Res Policy Syst 2014; 12: 35.

Mohr PE, Tunis SR. Medical and pharmacy coverage decision making at the population level. J Manag Care Spec Pharm 2014; 20(6): 547-54.

Cruz TM. Assessing access to care for transgender and gender nonconforming people: a consideration of diversity in combating discrimination. Soc Sci Med 2014; 110: 65-73.

Kazi DS, Mark DB. The economics of heart failure. Heart Fail Clin 2013; 9(1): 93-106.

Frank E. Aging and the market in the United States. Int J Health Serv 2001; 31(1): 133-46.

van Beek I. Maybe not perfect—but surely good enough?. Int J Drug Policy 2012; 2(23): 108.

Choudhry NK. Improving the pathway from cardiovascular medication prescribing to longer-term adherence: new results about old issues. Circ Cardiovasc Qual Outcomes 2010; 3(3): 223-5.

do Lago RF, Costa Ndo R. Antiretroviral manufacturers and the challenge of universal access to drugs through the Brazilian National STD/AIDS Program. Cad Saude Publica 2009; 25(10): 2273-84.

Rowett D, Ravenscroft PJ, Hardy J, et al. Using national health policies to improve access to palliative care medications in the community. J Pain Symptom Manage 2009; 37(3): 395-402.

McGivney MS, Meyer SM, Duncan-Hewitt W, et al. Medication therapy management: its relationship to patient counseling, disease management, and pharmaceutical care. J Am Pharm Assoc 2007; 47(5): 620-8.

Herrera E, Rocafort J, De Lima L, et al. Regional palliative care program in Extremadura: an effective public health care model in a sparsely populated region. J Pain Symptom Manage 2007; 33(5): 591-8.

Haylock PJ, Mitchell SA, Cox T, et al. The cancer survivor's prescription for living. Am J Nurs 2007; 107(4): 58-70.

Zabinski RA. Evidence based health benefits management. J Manag Care Pharm 2006; 12(7 Supp B): S12-6.

Witten B. Medicare prescription drug coverage (part D): challenges and opportunities. Nephrol News Issues 2006; 20(9): 14.

Bodenheimer T. High and rising health care costs. Part 3: the role of health care providers. Ann Intern Med 2005; 142(12 Pt 1): 996-1002.

Møldrup C. No cure, no pay. BMJ 2005; 330(7502): 1262-4.

Schein OD, Bressler NM, Price P. Photodynamic therapy with verteporfin: observations on the introduction of a new treatment into clinical practice. Arch Ophthalmol 2005; 123(1): 58-63.

Locock L, Bucknall T, Titler MG. Prescribing organizational change--what works for whom?. Worldviews Evid Based Nurs 2004; 1(4).

Mardones F. Inequality of health care for the elderly in Chile. Rev Med Chil 2004; 132(7): 865-72.

Taverne B. Free dispensing of antiretroviral treatments in Africa. Bull Soc Pathol Exot 2003; 96(3): 241-4.

Mossé PR, Takeuchi M. The Japanese health system: lasting reform is impossible. Med Sci (Paris) 2003; 19(2): 223-30.

Gray J. Drug policy: an oxymoron? Healthc Pap 2002; 3(1): 56-62; discussion 87-94.

Coleman CI, Reddy P, Quercia RA, et al. A medication assistance program to increase access to health-care. Conn Med 2001; 65(12): 711-3.

Levine S, Campen D, Millares M, et al. Kaiser Permanente's prescription drug benefit. Health Aff (Millwood) 2000; 19(2): 185-90.

Chambers JD. Do changes in drug coverage policy point to an increased role for cost-effectiveness analysis in the USA? Pharmacoeconomics 2014; 32(8): 729-33.

Chorniy A. Essays on the health economics of pharmaceuticals. All Dissertions, 2005.

Willis M, Persson U, Zoellner Y, et al. Reducing uncertainty in value-based pricing using evidence development agreements: the case of continuous intraduodenal infusion of levodopa/carbidopa (Duodopa®) in Sweden. Appl Health Econ Health Policy 2010; 8(6): 377-86.

Timmins LL. Three essays in health economics: determinants of individual health, medical care use, and treatment. PhD diss University of British Columbia, 2015.

de Gier H. Financing pharmaceutical care in the Dutch health system. Journal of Pharmaceutical Finance, Economics & Policy 2004; 12: 181-188.

Cheung FM, Woo J, Law CK, et al. Health systems: challenges, visions, and reforms from a comparative global perspaective. Chinese University of Hong Kong, 2013.

Wagstaff A, Yu S. Do health sector reforms have their intended impacts? The World Bank's Health VIII project in Gansu province, China. J Health Econ 2007; 26(3): 505-35.

Cetta MG, Asplin BR, Fields WW, et al. Emergency medicine and the debate over the uninsured: a report from the task force on health care and the uninsured. Ann Emerg Med 2000; 36(3): 243-6.

Mehta SH, Thomas DL, Sulkowski MS, et al. A framework for understanding factors that affect access and utilization of treatment for hepatitis C virus infection among HCV-mono-infected and HIV/HCV-co-infected injection drug users. AIDS 2005; 19 Suppl 3: S179-89.

Shrank WH, Joseph GJ, Choudhry NK, et al. Physicians' perceptions of relevant prescription drug costs: do costs to the individual patient or to the population matter most? Am J Manag Care 2006; 12(9): 545-51.

Stefanacci RG. Generic drugs... just what the MMA rrdered. P AND T 2005; 30(8): 462.

Powell HB, Adamson AS. Medicare Part D payments for brand and generic drugs prescribed by dermatologists. J Am Acad Dermatol 2018; 79(3): 575-577.

Lemire F. Pharmacare 2020?. Can Fam Physician 2019; 65(4): 304-.

Rawson NS, Adams J. Access to new drugs for rare disorders in Canada. CMAJ 2018; 190(27): E840-.

Naci H, Kesselheim AS. Specialty drugs - a distinctly American phenomenon. N Engl J Med 2020; 382(23): 2179-2181.

– Not a review and no data on effect of cost-sharing and/or prescription drug insurance

Luffman, J. Out-of-pocket spending on prescription drugs. Statistics Canada, 2005; 17(4): 5-13

Lo Sasso AT, Lyons JS. The effects of copayments on substance abuse treatment expenditures and treatment reoccurrence. Psychiatr Serv 2002; 53(12): 1605-11.

Kwan J, Razzaq A, Leiter LA, et al. Low socioeconomic status and absence of supplemental health insurance as barriers to diabetes care access and utilization. Can J Diabetes 2008; 32(3): 174-81.

Cohen J, Chee J. Pharmacy benefit managers and Medicare beneficiary access to prescription drugs. Drug information journal 2001; 35(2): 569-76.

Schoen C, Osborn R, Squires D, et al. How health insurance design affects access to care and costs, by income, in eleven countries. Health Aff 2010; 29(12): 2323-34.

Simon SR, Gurwitz JH. Drug therapy in the elderly: improving quality and access. Clin Pharmacol Ther 2003; 73(5): 387-93.

Werba JP, Khandelwal S, Nambiar L, et al. Health system barriers and facilitators to medication adherence for the secondary prevention of cardiovascular disease: a systematic review. Nutr Metab Cardiovasc Dis 2017; 27(1): e42-3.

Zillich AJ, Jaynes HA, Snyder ME, et al. Evaluation of specialized medication packaging combined with medication therapy management: adherence, outcomes, and costs among Medicaid patients. Med Care 2012; 50(6): 485-93.

Makinen M, Waters H, Rauch M, et al. Inequalities in health care use and expenditures: empirical data from eight developing countries and countries in transition. Bull World Health Organ 2000; 78(1): 55-65.

Yang Z, Norton E. How much would a medicare prescription drug benefit cost?. Journal of Pharmaceutical Finance, Economics & Policy 2006; 15(2): 97.

Wang C. Three Essays in Health Economics. 2013.

Grootendorst PV, Dolovich LR, Holbrook AM, et al. The impact of reference pricing of cardiovascular drugs on health care costs and health outcomes: evidence from British Columbia -- Volume II: Technical Report, Quantitative Studies in Economics and Population Research Reports 370, McMaster University, 2002.

Value-based Insurance Design

Look KA. Value-based insurance design and medication adherence: opportunities and challenges. Am J Manag Care 2015; 21: e78-90

Lee JL, Maciejewski M, Raju S, et al. Value-based insurance design: quality improvement but no cost savings. Health Aff 2013; 32: 1251-1257. DOI: 10.1377/hlthaff.2012.0902

Tang KL, Barnieh L, Mann B, et al. A systematic review of value-based insurance design in chronic diseases. Am J Manag Care 2014; 20: e229-241

Fairman KA, Curtiss FR. What do we really know about VBID? Quality of the evidence and ethical considerations for health plan sponsors. J Manag Care Pharm 2011; 17(2): 156-74

Fendrick AM, Martin JJ, Weiss AE. Value-based insurance design: more health at any price. Health Serv Res 2012; 47(1 Pt 2): 404-13

Lee JL, Maciejewski M, Raju S, Shrank WH, Choudhry NK. Value-based insurance design: quality improvement but no cost savings. Health Aff 2013; 32(7): 1251-7

Thomson S, Schang L, Chernew ME. Value-based cost sharing in the United States and elsewhere can increase patients' use of high-value goods and services. Health Aff (Millwood) 2013; 32(4): 704-12

Choudhry NK, Fischer MA, Smith BF, et al. Five features of value-based insurance design plans were associated with higher rates of medication adherence. Health Aff (Millwood) 2014; 33(3): 493-501

Blecker E. Achieving medication adherence through value-based insurance design. Find Brief 2015; 42(6): 1-2

Gibson TB, Maclean RJ, Chernew ME, Fendrick AM, Baigel C. Value-based insurance design: benefits beyond cost and utilization. Am J Manag Care 2015; 21(1): 32-5

Ogbechie OA, Hsu J. Systematic review of benefit designs with differential cost sharing for prescription drugs. Am J Manag Care 2015; 21(5): e338-48

Agarwal R, Gupta A, Fendrick AM. Value-Based Insurance Design Improves Medication Adherence Without An Increase In Total Health Care Spending. Health Aff (Millwood) 2018; 37(7): 1057-64

Krack G. How to make value-based health insurance designs more effective? A systematic review and meta-analysis. The European journal of health economics 2019; 20: 841-856. 2019/03/30. DOI: 10.1007/s10198-019-01046-1

Managed-care setting

Page RL, Barton P, and Nair K. Effect of cost-sharing for prescription medications on health outcomes in older adults: a critical review of the literature and potential implications for managed care Consult Pharm 2008; 23(1), 44-54

Older version of a relevant review

Austvoll-Dahlgren A, Aaserud M, Vist G, et al. Pharmaceutical Policies: Effects of Cap and Co-Payment on Rational Drug use Cochrane Database of Systematic Reviews 2008; 23(1): CD007017.

Appendix E. List of Canadian studies included in reviews

Greenlick MR, Darsky BJ. A comparison of general drug utilization in a metropolitan community with utlization under a drug prepayment plan. Am J Public Health 1968; 58(11).

Turner J, Wright E, Mendella L, Anthonisen N. Predictors of patient adherence to long-term home nebulizer therapy for COPD. The IPPB Study Group. Intermittent Positive Pressure Breathing. Chest 1995; 108(2): 394-400.

Grootendorst PV. Health care policy evaluation using longitudinal insurance claims data: an application of the panel Tobit estimator. Health Econ 1997; 6(4): 365-82.

Grootendorst PV, Feeny DH, Furlong W. Does it matter whom and how you ask? inter- and intra-rater agreement in the Ontario Health Survey. J Clin Epidemiol 1997; 50(2): 127-35.

Grootendorst PV, O'Brien BJ, Anderson GM. On becoming 65 in Ontario. Effects of drug plan eligibility on use of prescription medicines. Med Care 1997; 35(4): 386-98.

Poirier S, LeLorier J, Page V, Lacour A. The effect of a $2 co-payment on prescription refill rates of Quebec elderly and its relationship to socio-economic status. Canadian Pharmacy Journal 1998; 131(1): 30-4.

Blais L, Castilloux A, Couture J, LeLorier J. Impact of the Quebec cost sharing drug plan on asthmatic patients receiving social assistance. Canadian Journal of Clinical Pharmacology 1999; 6(1): 42.

Hux J, Naylor C, Fielding D. The Ontario Drug Benefit Program Copayment: its impact on access for Ontario seniors and charges to the program. Toronto: Institute for Clinical Evaluative Science 1999.

Narine L, Senathirajah M, Smith T. Evaluating reference-based pricing: initial findings and prospects. CMAJ 1999; 161(3): 286-8.

Bursey F, Crowley M, Janes C, Turner CJ. Cost analysis of a provincial drug program to guide the treatment of upper gastrointestinal disorders. CMAJ 2000; 162(6): 817-23.

Fassbender K, Pickard S. A policy impact analysis of pharmaceutical cost containment strategies in Alberta. Working Paper 00-12: Institute of Health Economics, 2000.

Blais L, Boucher JM, Couture J, Rahme E, LeLorier J. Impact of a cost-sharing drug insurance plan on drug utilization among older people. J Am Geriatr Soc 2001; 49(4): 410-4.

Grootendorst P, Dolovich LR, Holbrook AM, Levy AR, O'Brien BJ. The Impact of Reference Pricing of Cardiovascular Drugs on Health Care Costs and Health Outcomes: Evidence from British Columbia. Ottawa: Canadian Health Services Research Foundation, 2001.

Kozyrskyj AL, Mustard CA, Cheang MS, Simons FE. Income-Based Drug Benefit Policy: Impact on Receipt of Inhaled Corticosteroid Prescriptions by Manitoba Children with Asthma. Can Med Assoc J 2001; 165(7): 897-902.

Kozyrskyj AL, Mustard CA, Simons FE. Socioeconomic status, drug insurance benefits, and new prescriptions for inhaled corticosteroids in schoolchildren with asthma. Arch Pediatr Adolesc Med 2001; 155(11): 1219-24.

MacCara ME, Sketris IS, Comeau DG, Weerasinghe SD. Impact of a limited fluoroquinolone reimbursement policy on antimicrobial prescription claims. Ann Pharmacother 2001; 35(7-8): 852-8.

Narine L, Senathirajah M, Smith T. An Assessment of the Impact of Reference-Based Pricing Policies on the H2 Antagonist Market in British Columbia, Canada. Journal of Research in Pharmaceutical Economics 2001; 11(1): 63-78.

Tamblyn R, Laprise R, Hanley JA, et al. Adverse events associated with prescription drug cost-sharing among poor and elderly persons. JAMA 2001; 285(4): 421-9.

Alan S, Crossley TF, Grootendorst P, Veall MR. The effects of drug subsidies on out-of-pocket prescription drug expenditures by seniors: regional evidence from Canada. J Health Econ 2002; 21(5): 805-26.

Grootendorst P, Levine M. Do drug plans matter? Effects of Drug Plan Eligibility on Drug Use among the Elderly, Social Assistance Recipients and the General Population. Hamilton: McMaster University, 2002.

Hazlet TK, Blough DK. Health services utilization with reference drug pricing of histamine(2) receptor antagonists in British Columbia elderly. Med Care 2002; 40(8): 640-9.

Marshall JK, Grootendorst PV, O'Brien BJ, Dolovich LR, Holbrook AM, Levy AR. Impact of reference-based pricing for histamine-2 receptor antagonists and restricted access for proton pump inhibitors in British Columbia. CMAJ 2002; 166(13): 1655-62.

Pilote L, Beck C, Richard H, Eisenberg MJ. The effects of cost-sharing on essential drug prescriptions, utilization of medical care and outcomes after acute myocardial infarction in elderly patients. CMAJ 2002; 167(3): 246-52.

Schneeweiss S, Maclure M, Soumerai SB. Prescription duration after drug copay changes in older people: methodological aspects. J Am Geriatr Soc 2002; 50(3): 521-5.

Schneeweiss S, Soumerai SB, Glynn RJ, Maclure M, Dormuth C, Walker AM. Impact of reference-based pricing for angiotensin-converting enzyme inhibitors on drug utilization. CMAJ 2002; 166(6): 737-45.

Schneeweiss S, Walker AM, Glynn RJ, Maclure M, Dormuth C, Soumerai SB. Outcomes of reference pricing for angiotensin-converting-enzyme inhibitors. N Engl J Med 2002; 346(11): 822-9.

Suissa S, Ernst P, Kezouh A. Regular use of inhaled corticosteroids and the long term prevention of hospitalisation for asthma. Thorax 2002; 57(10): 880-4.

Alan S, Crossley TF, Grootendorst PV, Veall MR. Out-of-pocket Prescription Drug Expenditures and Public Prescription Drug Programs: Provincial Evidence from Canada. Hamilton, ON: McMaster University, 2003.

Blais L, Couture J, Rahme E, LeLorier J. Impact of a cost sharing drug insurance plan on drug utilization among individuals receiving social assistance. Health Policy 2003; 64(2): 163-72.

Campbell CA, Cooke CA, Weerasinghe SDS, Sketris IS, McLean-Veysey PR, Skedgel CD. Topical corticosteroid prescribing patterns following changes in drug benefit status. Ann Pharmacother 2003; 37(6): 787-93.

Schneeweiss S, Soumerai SB, Maclure M, Dormuth C, Walker AM, Glynn RJ. Clinical and economic consequences of reference pricing for dihydropyridine calcium channel blockers. Clin Pharmacol Ther 2003; 74(4): 388-400.

Livingstone T, Lix L, McNutt M, Morris E. An Investigation of the Impact of Supplementary Health Benefits for Low-income Families in Saskatchewan. Canadian Journal of Public Health 2004; 95(1): 74-8.

Schneeweiss S, Dormuth C, Grootendorst P, Soumerai SB, Maclure M. Net health plan savings from reference pricing for angiotensin-converting enzyme inhibitors in elderly British Columbia residents. Med Care 2004; 42(7): 653-60.

Schneeweiss S, Maclure M, Carleton B, Glynn RJ, Avorn J. Clinical and economic consequences of a reimbursement restriction of nebulised respiratory therapy in adults: direct comparison of randomised and observational evaluations. BMJ 2004; 328(7439): 560.

Alan S, Crossley TF, Grootendorst P, Veall MR. Distributional effects of 'general population' prescription drug programs in Canada. Canadian Journal of Economics 2005; 38(1): 128-48.

Anis AH, Guh DP, Lacaille D, et al. When patients have to pay a share of drug costs: effects on frequency of physician visits, hospital admissions and filling of prescriptions. Can Med Assoc J 2005; 173(11): 1335-40.

Blackburn DF, Dobson RT, Blackburn JL, Wilson TW. Cardiovascular morbidity associated with nonadherence to statin therapy. Pharmacotherapy 2005; 25(8): 1035-43.

Contoyannis P, Hurley J, Grootendorst P, Jeon S-H, Tamblyn R. Estimating the price elasticity of expenditure for prescription drugs in the presence of non-linear price schedules: an illustration from Quebec, Canada. Health Econ 2005; 14(9): 909-23.

Grootendorst PV, Marshall JK, Holbrook AM, Dolovich LR, O'Brien BJ, Levy AR. The impact of reference pricing of nonsteroidal anti-inflammatory agents on the use and costs of analgesic drugs. Health Serv Res 2005; 40(5 Pt 1): 1297-317.

Kephart G, Sketris IS, Bowles SK, Richard ME, Cooke CA. Impact of a criteria-based reimbursement policy on the use of respiratory drugs delivered by nebulizer and health care services utilization in Nova Scotia, Canada. Pharmacotherapy 2005; 25(9): 1248-57.

Smart M, Stabile M. Tax credits, insurance, and the use of medical care. Canadian Journal of Economics 2005; 38(2): 345-65.

Ackman ML, Graham MM, Hui C, Tsuyuki RT. Effect of a prior authorization process on antiplatelet therapy and outcomes in patients prescribed clopidogrel following coronary stenting. Can J Cardiol 2006; 22(14): 1205-8.

Caetano PA, Raymond CB, Morgan S, Yan L. Income-based drug coverage in British Columbia: the impact on access to medicines. Healthc Policy 2006; 2(2): e154-69.

Dormuth CR, Glynn RJ, Neumann P, Maclure M, Brookhart AM, Schneeweiss S. Impact of two sequential drug cost-sharing policies on the use of inhaled medications in older patients with chronic obstructive pulmonary disease or asthma. Clin Ther 2006; 28(6): 964-78; discussion 2-3.

Grootendorst P, Stewart D. A re-examination of the impact of reference pricing on anti-hypertensive drug plan expenditures in British Columbia. Health Econ 2006; 15(7): 735-42.

Mabasa VH, Ma J. Effect of a therapeutic maximum allowable cost (MAC) program on the cost and utilization of proton pump inhibitors in an employer-sponsored drug plan in Canada. J Manag Care Pharm 2006; 12(5): 371-6.

Marshall D, Gough J, Grootendorst P, et al. Impact of administrative restrictions on antibiotic use and expenditure in Ontario: time series analysis. J Health Serv Res Policy 2006; 11(1): 13-20.

Schneeweiss S, Maclure M, Dormuth CR, Glynn RJ, Canning C, Avorn J. A therapeutic substitution policy for proton pump inhibitors: clinical and economic consequences. Clin Pharmacol Ther 2006; 79(4): 379-88.

Bouchard M-H, Dragomir A, Blais L, Berard A, Pilon D, Perreault S. Impact of adherence to statins on coronary artery disease in primary prevention. Br J Clin Pharmacol 2007; 63(6): 698-708.

Li X, Guh D, Lacaille D, Esdaile J, Anis AH. The impact of cost sharing of prescription drug expenditures on health care utilization by the elderly: Own- and cross-price elasticities. Health Policy 2007; 82(3): 340-7.

Marshall DA, Willison DJ, Grootendorst P, et al. The effects of coxib formulary restrictions on analgesic use and cost: regional evidence from Canada. Health Policy 2007; 84(1): 1-13.

Rasmussen JN, Chong A, Alter DA. Relationship between adherence to evidence-based pharmacotherapy and long-term mortality after acute myocardial infarction. JAMA 2007; 297(2): 177-86.

Schneeweiss S, Patrick AR, Maclure M, Dormuth CR, Glynn RJ. Adherence to beta-blocker therapy under drug cost-sharing in patients with and without acute myocardial infarction. Am J Manag Care 2007; 13(8): 445-52.

Schneeweiss S, Patrick AR, Maclure M, Dormuth CR, Glynn RJ. Adherence to statin therapy under drug cost sharing in patients with and without acute myocardial infarction: a population-based natural experiment. Circulation 2007; 115(16): 2128-35.

Zhong H. Equity in Pharmaceutical Utilization in Ontario: A Cross-Section and Over Time Analysis. Canadian Public Policy 2007; 33(4): 487-507.

Dormuth CR, Maclure M, Glynn RJ, Neumann P, Brookhart AM, Schneeweiss S. Emergency hospital admissions after income-based deductibles and prescription copayments in older users of inhaled medications. Clin Ther 2008; 30 Spec No: 1038-50.

Jackevicius CA, Tu JV, Demers V, et al. Cardiovascular outcomes after a change in prescription policy for clopidogrel. N Engl J Med 2008; 359(17): 1802-10.

Ungar WJ, Kozyrskyj A, Paterson M, Ahmad F. Effect of cost-sharing on use of asthma medication in children. Arch Pediatr Adolesc Med 2008; 162(2): 104-10.

Wang PS, Patrick AR, Dormuth CR, et al. The impact of cost sharing on antidepressant use among older adults in British Columbia. Psychiatr Serv 2008; 59(4): 377-83.

Dormuth CR, Neumann P, Maclure M, Glynn RJ, Schneeweiss S. Effects of prescription coinsurance and income-based deductibles on net health plan spending for older users of inhaled medications. Med Care 2009; 47(5): 508-16.

Wang PS, Patrick AR, Dormuth C, et al. Impact of drug cost sharing on service use and adverse clinical outcomes in elderly receiving antidepressants. J Ment Health Policy Econ 2010; 13(1): 37-44.

Law MR, Cheng L, Dhalla IA, et al. The effect of cost on adherence to prescription medications in Canada. CMAJ 2012; 184: 297-302. DOI: 10.1503/cmaj.111270.

Zheng B, Poulose A, Fulford M, et al. A pilot study on cost-related medication nonadherence in Ontario. J Popul Ther Clin Pharmacol 2012; 19: e239-247. 2012/07/19.

Appendix F. List of reviews that focused specifically on value-based cost-sharing/insurance design.

Fairman KA, Curtiss FR. What do we really know about VBID? Quality of the evidence and ethical considerations for health plan sponsors. J Manag Care Pharm 2011; 17(2): 156-74.

Fendrick AM, Martin JJ, Weiss AE. Value-based insurance design: more health at any price. Health Serv Res 2012; 47(1 Pt 2): 404-13.

Lee JL, Maciejewski M, Raju S, Shrank WH, Choudhry NK. Value-based insurance design: quality improvement but no cost savings. Health Aff 2013; 32(7): 1251-7.

Thomson S, Schang L, Chernew ME. Value-based cost sharing in the United States and elsewhere can increase patients' use of high-value goods and services. Health Aff 2013; 32(4): 704-12.

Choudhry NK, Fischer MA, Smith BF, et al. Five features of value-based insurance design plans were associated with higher rates of medication adherence. Health Aff 2014; 33(3): 493-501.

Tang KL, Barnieh L, Mann B, et al. A systematic review of value-based insurance design in chronic diseases. Am J Manag Care 2014; 20(6): e229-41.

Gibson TB, Maclean RJ, Chernew ME, Fendrick AM, Baigel C. Value-based insurance design: benefits beyond cost and utilization. Am J Manag Care 2015; 21(1): 32-5.

Look KA. Value-based insurance design and medication adherence: opportunities and challenges. Am J Manag Care 2015; 21(1): e78-90.

Ogbechie OA, Hsu J. Systematic review of benefit designs with differential cost sharing for prescription drugs. Am J Manag Care 2015; 21(5): e338-48.

Agarwal R, Gupta A, Fendrick AM. Value-Based Insurance Design Improves Medication Adherence Without An Increase In Total Health Care Spending. Health Aff 2018; 37(7): 1057-64.

Krack G. How to make value-based health insurance designs more effective? A systematic review and meta-analysis. The European journal of health economics 2019; 20(6): 841-56.
